# Ligand-Induced Opening of a Cryptic Pocket in METTL14

**DOI:** 10.1021/acsbiomedchemau.5c00184

**Published:** 2026-03-10

**Authors:** Ivan Corbeski, Rajiv Kumar Bedi, Christian M. Matter, Fiona Stamm, Elena Bochenkova, Marcin Herok, Michael J. Hartshorn, Amedeo Caflisch

**Affiliations:** Department of Biochemistry, 27217 University of Zurich, Zurich 8057, Switzerland

**Keywords:** RNA-methyltransferase, m^6^A-RNA, epitranscriptomics, exosite ligands, protein crystallography, molecular dynamics

## Abstract

The complex of methyltransferase-like proteins 3 and 14 (METTL3–14) is the main human enzyme that deposits the most abundant internal mRNA modification, N^6^-methyladenosine (m^6^A). In the heterodimeric complex, METTL3 acts as a catalytic subunit while METTL14 is involved in mRNA binding and complex stabilization. Here, we present the discovery of small-molecule ligands that bind to a cryptic pocket in METTL14 by protein crystallography. A comparative analysis of crystal structures revealed that the METTL14 cryptic pocket is closed in the apo structure of METTL3–14, and in the structures of METTL3–14 in the complex with the cosubstrate *S*-adenosyl-methionine (SAM) and a large number of SAM-competitive inhibitors. We first discovered compounds **1** and **2** that bind to both the SAM pocket in METTL3 and the cryptic pocket in METTL14. With this structural information, we designed compound **3** that binds only to the METTL14 cryptic pocket. Compound **3** does not inhibit the catalytic activity of METTL3–14 but can be used as an anchor for heterobifunctional molecules. We propose a route for its further development into heterobifunctional ligands, e.g., proteolysis targeting chimeras (PROTACs).

## Graphical abstract

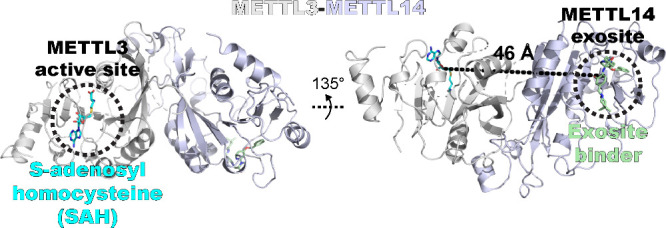

## Introduction

### Epitranscriptomics

RNA is at the heart of more than 170 chemical modifications grouped into the epitranscriptome.^1–3^ These modifications represent a layer of post-transcriptional gene expression control and are involved in a wide array of biological processes.^4–18^ N^6^-methyladenosine (m^6^A) is the most frequent internal modification of messenger RNA (mRNA) within the consensus sequence GGACU that is enriched near stop codons and in 3′ untranslated regions.^2,19–21^ m^6^A affects most aspects of mRNA regulation, i.e., alternative polyadenylation, splicing, nuclear export, stability, and translation initiation.^1,2,22–25^ m^6^A is also found in other RNA species, including lncRNAs, rRNAs, and snRNAs.^26–29^ The dynamic and reversible nature of m^6^A is regulated by its methyltransferase (writer), demethylase (eraser), and recognition (reader) proteins.^30,31^ While the latter two recognize or remove m^6^A modifications, the former are the starting point by installing the methyl mark.^32^ The complex of methyltransferase-like protein 3 (METTL3) and METTL14 (abbreviated as METTL3–14 in the following) is the main m^6^A-RNA methyltransferase (MTase).^33^

### m^6^A and Disease

The m^6^A modification on mRNA influences various physiological processes, and any misregulation could drive development of disease.^1,33–38^ Thus, proteins involved in epitranscriptomic regulation have been attracting attention as targets in drug discovery efforts.^8,39–44^ The biological roles of METTL3 and METTL14 are wide; for instance, METTL3 promotes translation and regulates cell proliferation, cell migration, and inflammatory response.^8,45,46^ The METTL3–14 heterodimer is involved in a wide variety of diseases including type 2 diabetes, viral infections, and several types of cancer.^47–49^ Numerous studies have shown that METTL3 is expressed at aberrantly high levels in several cancer types, suggesting a prominent role in cancer progression, and has been shown to positively regulate cancer development by promoting metastasis and tumor growth in malignancies such as acute myeloid leukemia (AML), lymphomas, and other types of cancer, e.g., bladder, lung, ovarian, colorectal, bone, liver, and gastric.^8,38,45,50–64^ METTL3-mediated m^6^A deposition is directly involved in the development of AML by promoting the translation of genes involved in cell growth, differentiation, and apoptosis.^51,63^ While it has been well established that METTL3–14 dysregulation is related to cancer development, the role of METTL3–14 varies in different cancer types, i.e., it can act as oncogene or tumor suppressor.^65^ Furthermore, other biological mechanisms may be influenced by m^6^A dysfunctional levels like the circadian clock, stem cell differentiation, or even viral gene expression.^35,66^ Hence, inhibiting the MTase function of METTL3–14 is a promising therapeutic strategy for several diseases.^67,68^

### METTL3 Inhibitors

METTL3–14 is the catalytic complex that transfers the methyl group from *S*-adenosylmethionine (SAM) to the substrate adenosine in RNA (Figure 1).^69–72^ METTL3 comprises a low-complexity region at the N-terminus, a zinc finger responsible for substrate binding, and the catalytic MTase domain at the C-terminus (Figure 1A).^73^ The METTL3 MTase domain has the catalytically active SAM binding site and adopts a Rossmann fold that is characteristic of Class I SAM-dependent MTases. METTL14 has an MTase domain, too, however, with a redundant active site of hitherto unknown function, and so-called RGG repeats at its C-terminus important for RNA and protein binding.^72,74^ METTL14 plays a structural role for complex stabilization and RNA binding. It forms a positively charged groove at the interface with METTL3, which is predicted to be the RNA binding site (Figure 1B).

**Figure 1:**
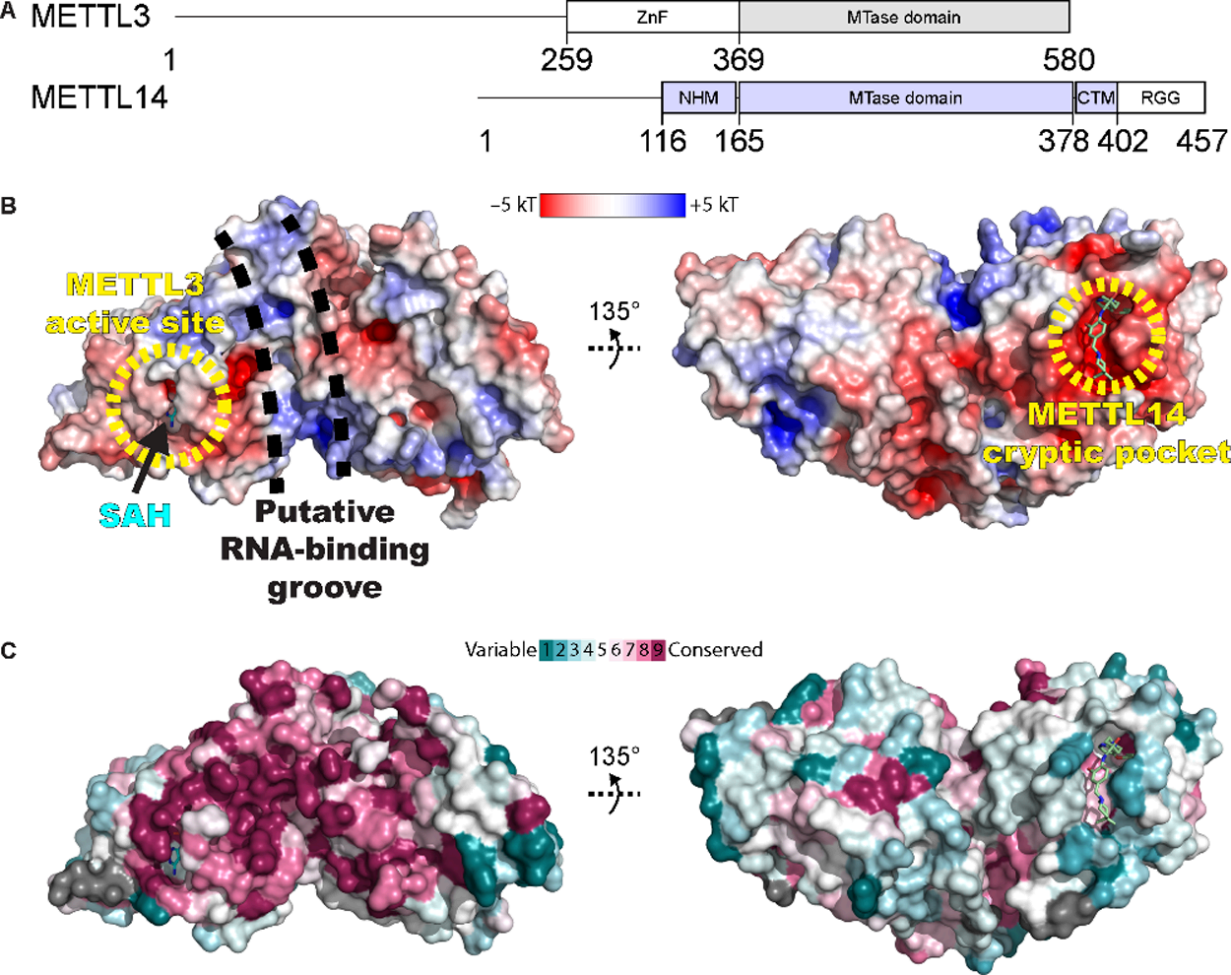
METTL3–14 domain architecture, electrostatic surface potential, binding pockets, and sequence conservation. (A) Domain architecture of METTL3 and METTL14. ZnF = zinc finger, NHM = N-terminal α-helical motif, CTM = C-terminal motif, RGG = arginine-glycine-glycine motif. (B) Crystal structure (PDB code 9RS3) of the MTase domains of METTL3–14 with SAH and compound **3** (shown as sticks) in the METTL3 SAM pocket and the METTL14 exosite (cryptic pocket), respectively. The surface rendering is colored according to the electrostatic potential. (C) Same as (B), with the surface rendering colored according to the sequence conservation score obtained from Protein Data Bank (PDB) ID 5IL0 using Consurf-DB;^75,76^ gray: insufficient data. *Panel A and portions of panel B are adopted from* Corbeski, I.; Vargas-Rosales, P. A.; Bedi, R. K.; Deng, J.; Coelho, D.; Braud, E.; Iannazzo, L.; Li, Y.; Huang, D.; Ethève-Quelquejeu, M.; Cui, Q.; Caflisch, A. The Catalytic Mechanism of the RNA Methyltransferase METTL3.*eLife* **2024**, 13, e92537, 10.7554/eLife.92537.3. *Used under the Creative Commons Attribution 4.0 International License (CC BY 4.0).*

The METTL3–14 complex is an attractive target for inhibition by small molecules.^67,68,77–80^ The decrease in the m^6^A level causes apoptosis and reduces the invasiveness of cancer cells, suggesting that METTL3–14 is a therapeutic target for treating various cancers in which it acts as oncogene.^65,79,81,82^ SAM-competitive small-molecule inhibitors of METTL3 have displayed high potency against AML.^67,68,80^ Importantly, it was shown that small-molecule inhibition of METTL3 is sufficient to induce apoptosis and differentiation in AML cells and in a mouse model of the disease but not in normal nonleukemic hematopoietic cells.^68,77^

As an alternative to small molecules, proteolysis targeting chimeras (PROTACs) offer a versatile and potentially powerful approach for targeted inhibition through protein degradation.^83^ Several PROTACs have been developed for the targeted degradation of METTL3–14 and shown to be effective in different cancer cell lines.^84–88^ One limitation of SAM-competitive METTL3 binders, both small molecules and PROTACs, is that they inhibit the methyltransferase activity of METTL3–14. This makes it impossible to determine effects of these binders independent of METTL3–14 inhibition.

Other than small molecules and PROTACs as METTL3 inhibitors, peptides have also been developed that interfere with METTL3 function.^89,90^ These peptides are based on an α-helix in METTL3 that interacts with METTL14 in the heterodimeric complex. They thereby disrupt the METTL3–14 complex and lead to its cellular degradation. Furthermore, allosteric inhibitors of METTL3 have been described; however, their mechanisms of action remain mostly elusive due to the lack of structural information on their binding mechanism.^91,92^

In this work, we present small-molecule ligands of a cryptic pocket of METTL14, which originate from structure-based design campaigns for SAM-competitive METTL3 inhibitors. The serendipitous discovery of two METTL3–14 inhibitors that occupy both the SAM pocket in METTL3 and a cryptic pocket in METTL14 was followed by the design of a compound that binds only to the METTL14 cryptic pocket. These binders represent starting points for functionalization into heterobifunctional molecules that target the noncatalytic subunit of METTL3–14 and thus do not inhibit the methyltransferase function of METTL3 in the cell.

## Results and Discussion

### Discovery of Binders in a Cryptic Pocket of METTL14

We solved several crystal structures of METTL3–14 in the complex with SAM-competitive small molecules during a previous optimization campaign by medicinal chemistry that culminated in the single-digit nanomolar inhibitor UZH2.^67^ Surprisingly, the crystal structure of the submicromolar inhibitor **1** (Table 1; it is compound **11** in ref 67) revealed electron density in a pocket of METTL14, which is occluded in all the previously disclosed apo and holo structures of METTL3–14 (Figure 2A, Figures S1–S4, and Table S1). We call this cryptic pocket in METTL14 the exosite as it is located at approximately 46 Å from the catalytic SAM pocket in METTL3. The exosite consists of residues that are mostly variable based on sequence conservation analysis (Figure 1C). In the open state, it shows a negative electrostatic potential (Figure 1B). Since the putative SAM binding site in METTL14 is blocked by a different conformation of its active site loop, the exosite binders cannot bind there, but instead to the cryptic pocket adjacent to it (Figure S1).

**Table 1:**
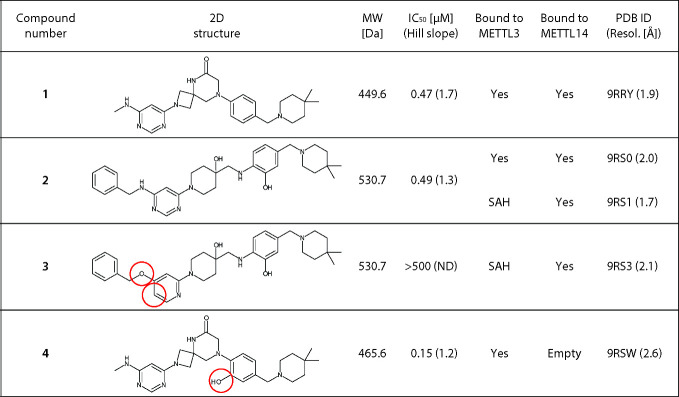
Summary of the Compounds, IC_50_ Values, and Crystallography^a^

a The inhibitory activity was measured by the reader-based HTRF assay (see Materials and Methods and Supplementary Figure S5).^94^ The crystal structures were obtained by soaking the compounds into crystals of the METTL3-14 complex that had SAH bound in the METTL3 active site. Modifications of compounds **3** and **4** compared to their parent compounds **2** and **1**, respectively, are emphasized (red circles). ND = not determined.

**Figure 2:**
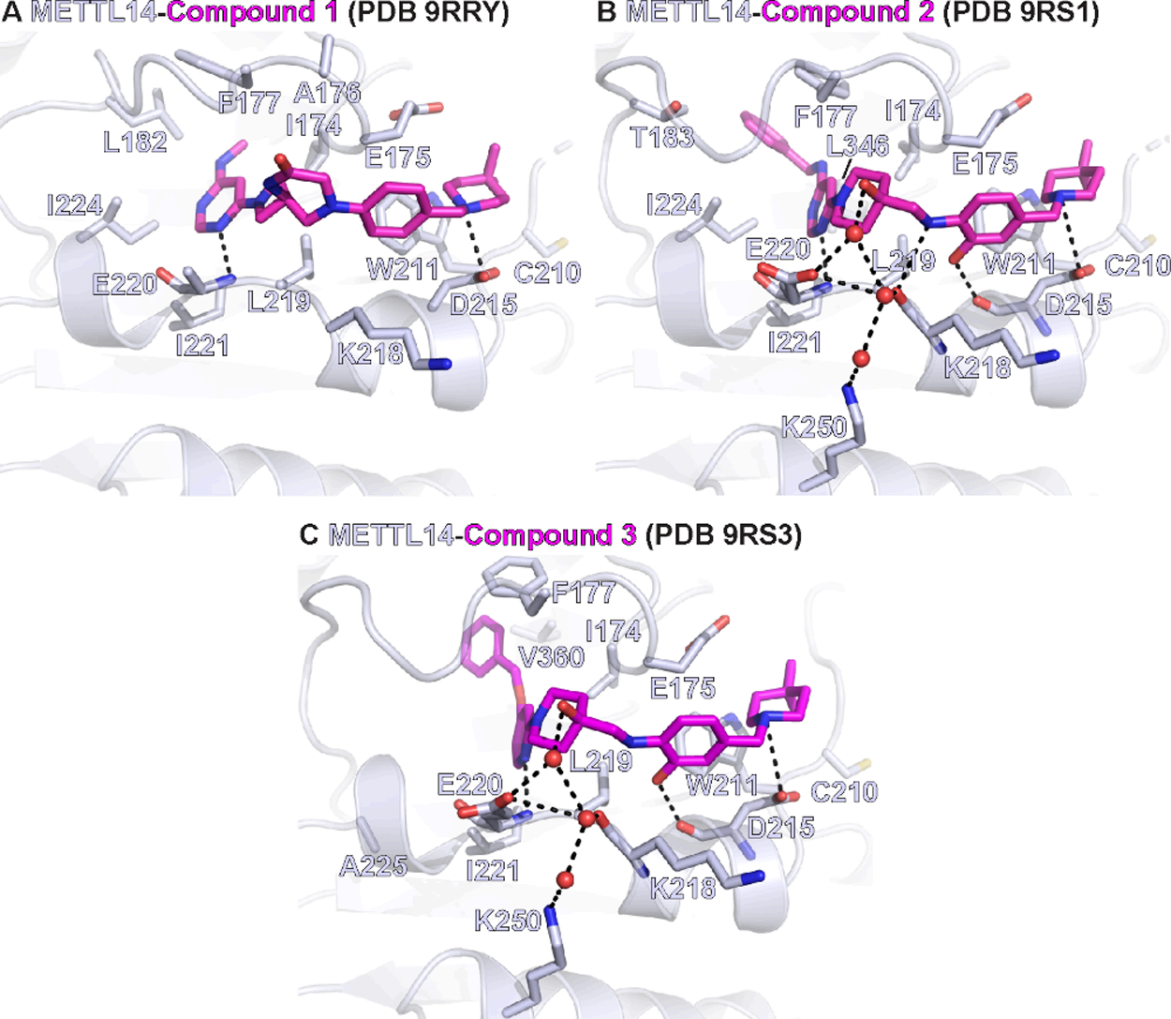
Interactions of exosite binders and METTL14. Close-up views of compounds **1** (A), **2** (B), and **3** (C) (shown as sticks) in the METTL14 exosite (backbone shown in cartoon representation, residues forming the binding pocket environment as sticks, waters as red spheres). Black dashes indicate polar contacts in the crystal structure.

The tertiary amine in the piperidine ring of compound **1** is positively charged at physiological pH and forms a salt bridge with the side chain of D215 in METTL14 (Figure 2A). Additional favorable interactions include a hydrogen bond between one of the two nitrogen atoms of the pyrimidine ring and the backbone NH of Glu220, while the amide group in the spiro ring points toward solvent. Comparison of the METTL3–14 complex in its apo state and when bound with compound **1** shows a significant displacement of METTL14 segments 174–179 and unfolding of its one-turn 3_10_-helix (178–181) (Figure 3A). The side chain of Ile179 moves toward solvent by 7.9 Å (measured at the Cβ atom) for accommodating the pyrimidine ring of compound **1**.

**Figure 3:**
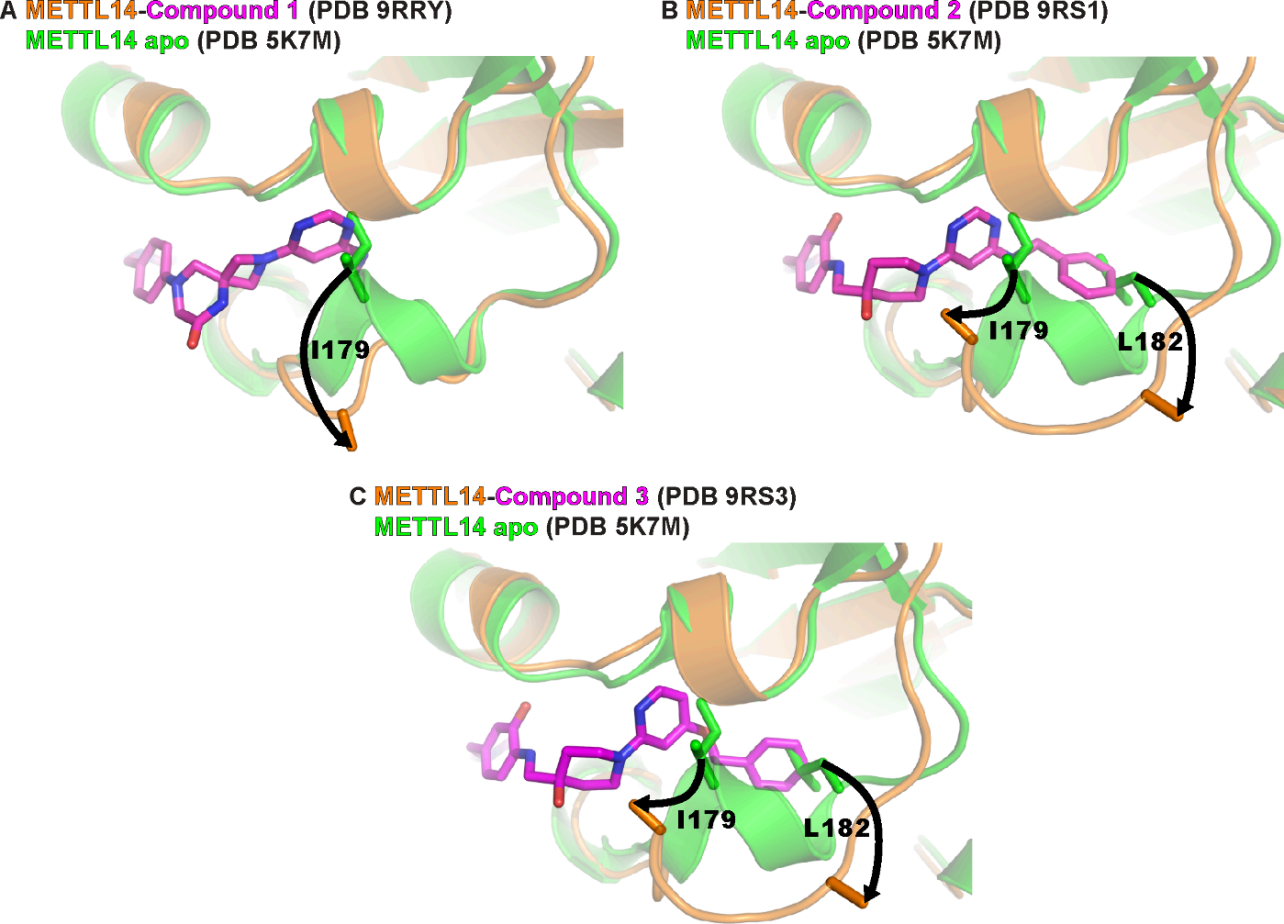
Displacement of the segment with residues 174–183 of METTL14 for accommodating the exosite binders in the cryptic pocket. Structural overlay of METTL3–14 (backbone shown in cartoon representation, indicated residues as sticks) in the apo state and in the complexes with compounds **1** (A), **2** (B), and **3** (C) (shown as sticks). Color coding is indicated at the top of each panel. Black arrows emphasize the displacements of Ile179 and Leu182. Note that of the side chains of Ile179 and Leu182, only the Cβ atoms are resolved in the crystal structures with the compounds due to lack of electron density, probably due to flexibility.

During another optimization campaign started from a different hit compound, we discovered that the submicromolar METTL3–14 inhibitor **2** occupies the same cryptic pocket in METTL14 as compound **1** (Table 1 and Figure 2B).^93^ In two soaking experiments with inhibitor **2** at 200 and 50 mM, we obtained two different crystal structures of METTL3–14, respectively. In the structure obtained at higher concentrations, inhibitor **2** occupies both the SAM pocket in METTL3 and the METTL14 exosite (PDB 9RS0), while in the other structure, it is found only in the latter (PDB 9RS1). In both crystal structures, the bulkier phenyl group of inhibitor **2** (2D structure in Table 1) requires a substantial displacement of a larger segment (residues 174–183) of METTL14 compared to the smaller amino-methyl group of compound **1**. Besides the shift of Ile179, there is also a rotation of the Leu182 side chain toward solvent resulting in a distance of 7.2 Å (measured at Cβ) with respect to its position in the apo structure (Figure 3B). Note that the side chain of Leu182 is not displaced in the complex with compound **1** as its amino-methyl group does not reach the pocket occupied by the Leu182 side chain.

### Design of an Exosite Binder that Does Not Occupy the SAM Pocket in METTL3

With the crystal structures of two exosite binders in hand (compounds **1** and **2**), we decided to design compounds that would bind only to the cryptic pocket of METTL14 and not to the SAM pocket of METTL3. For this purpose, compound **2** was modified (Table 1 and Table S2). While the majority of these compounds had weakened METTL3 binding as indicated by their weaker inhibitory potencies, they were also not bound to METTL14 in crystals of METTL3–14 (Table S2). However, compound **3**, which resulted by changing only two atoms of compound **2**, had no inhibitory effect on METTL3–14 catalytic activity and was bound only to METTL14 in crystals of METTL3–14 (Table 1). These two modifications were inspired by hydrogen bonds between two nitrogen atoms in compound **2** and the SAM pocket of METTL3 (Figure S2). One nitrogen in the pyrimidine ring was exchanged with a CH, and the amine nitrogen next to the pyrimidine was replaced by an oxygen atom. Compound **3** did not show any inhibitory potency in the METTL3–14 enzymatic assay (Figure S5), confirming the lack of binding to METTL3. Upon soaking into METTL3–14 crystals, compound **3** was only bound to METTL14 and did not displace the SAH bound to METTL3. Compound **3** binds to METTL14 in a very similar manner as compound **2** (see Figures 2 and 3). Importantly, the interactions of compound **3** with the exosite pocket and conformational changes as observed with the inhibitors **1** and **2** can serve as guiding principles for further development of these binders (Figure 4). The pyridine ring of compound **3** (similar to the pyrimidine rings of compounds **1** and **2**) displaces the side-chain Ile179 and replaces its hydrophobic contacts with Ile221 and Ile224 (Figure 4A, see also Figure 2). The benzyl ring present in compounds **2** and **3** reaches deep into the METTL14 cryptic pocket where it displaces the side chain of Leu182 and replaces its hydrophobic contacts with Phe177 and Ile179 (Figure 4B). Notably, the dimethylpiperidine groups of compounds **1**–**3** displace the side chain of Lys209 and replace its salt bridge with Asp215 (Figure 4C). Furthermore, the dimethylpiperidine group forms hydrophobic contacts with the side chain of Trp211.

The lack of inhibitory activity of compound **3** suggests that binding to the exosite pocket on METTL14 does not induce an allosteric effect on catalysis. Hence, in contrast to ligands that occupy the SAM pocket of METTL3, the exosite binders do not inhibit the methyltransferase activity of METTL3. Thus, development of heterobifunctional molecules such as PROTACs could take advantage of METTL14 exosite binders without disturbing the methyltransferase activity of METTL3. More specifically, one could consider extending compound **3** by a linker at the hydroxyl group of the central piperidine, which is exposed to solvent (Figure 4A,B). To further characterize compound **3**, we carried out several cellular assays (Figures S6–S8).

Compounds **2** and **3** showed a reduction of cell viability in the AML cell line THP-1 with similar GI_50_ values as the well-characterized METTL3 inhibitor UZH2 (Figure S6). However, high Hill slopes of the dose response curves for compounds **2** and **3** indicate that their effect could be unspecific. In the prostate cancer cell line PC-3, compounds **2** and **3** show higher antiproliferative activity compared to UZH2 with an acceptable Hill slope. However, both compounds also markedly decrease the cell viability of the noncancerous cell line MRC-5. While the antiproliferative activity in PC-3 could originate from a specific mechanism, the fact that compounds **2** and **3** lead also to reduced cell viability in a noncancerous cell line indicates that there might be also unspecific effects. In cellular thermal shift assays (CETSAs), we could show a small stabilization of both METTL3 and METTL14 when compounds **2** and **3** were added to PC-3 cells (Figure S7). Finally, we tested the ability of compounds **2** and **3** to reduce m^6^A/A levels in the AML cell line MOLM-13 (Figure S8). While compound **2** showed some inhibitory effect as expected from its in vitro potency and binding capability to METTL3, compound **3** did not lead to any reduction. This is as expected as compound **3** does not inhibit METTL3–14 in the biochemical assay.

We further tested the ability of compound **3** to bind METTL3–14 in vitro (Figures S9–S11 and Table S3). We conducted thermal shift assays where we titrated the METTL3–14 complex with compounds (Figure S9). There, we could detect a dose-dependent stabilization of the complex with compound **3** up to 125 μM while higher concentrations led to destabilization, possibly due to aggregates formed by the compound. Furthermore, we conducted a crystallization experiment where we added different concentrations of compounds **1**, **2**, and **3** to crystals of METTL3–14/SAH (Figures S10 and S11 and Table S3). We titrated the crystals with substantially lower concentrations than for the initial structure determination where 50–200 mM of compound was used. At 1–25 mM, compound **1** occupied the SAM pocket in METTL3 but it was not present in the cryptic pocket of METTL14 (Table S3). Compound **2** was also present in the SAM pocket of METTL3 and showed only partial density in the METTL14 cryptic pocket with minimal loop rearrangement at 25 mM and weaker density with no loop rearrangement at 5 mM (Figure S10). Compound **3** showed complete density in the METTL14 cryptic pocket with complete loop rearrangement at 25 and 5 mM, and weaker density with minimal loop rearrangement at 1 mM (Figure S11). At all concentrations of compound **3** (i.e., from 1 to 200 mM), the SAM pocket of METTL3 was occupied by SAH (Table S3). These results indicate that compound **3** is the strongest METTL14 binder with an affinity in the low millimolar range.

**Figure 4:**
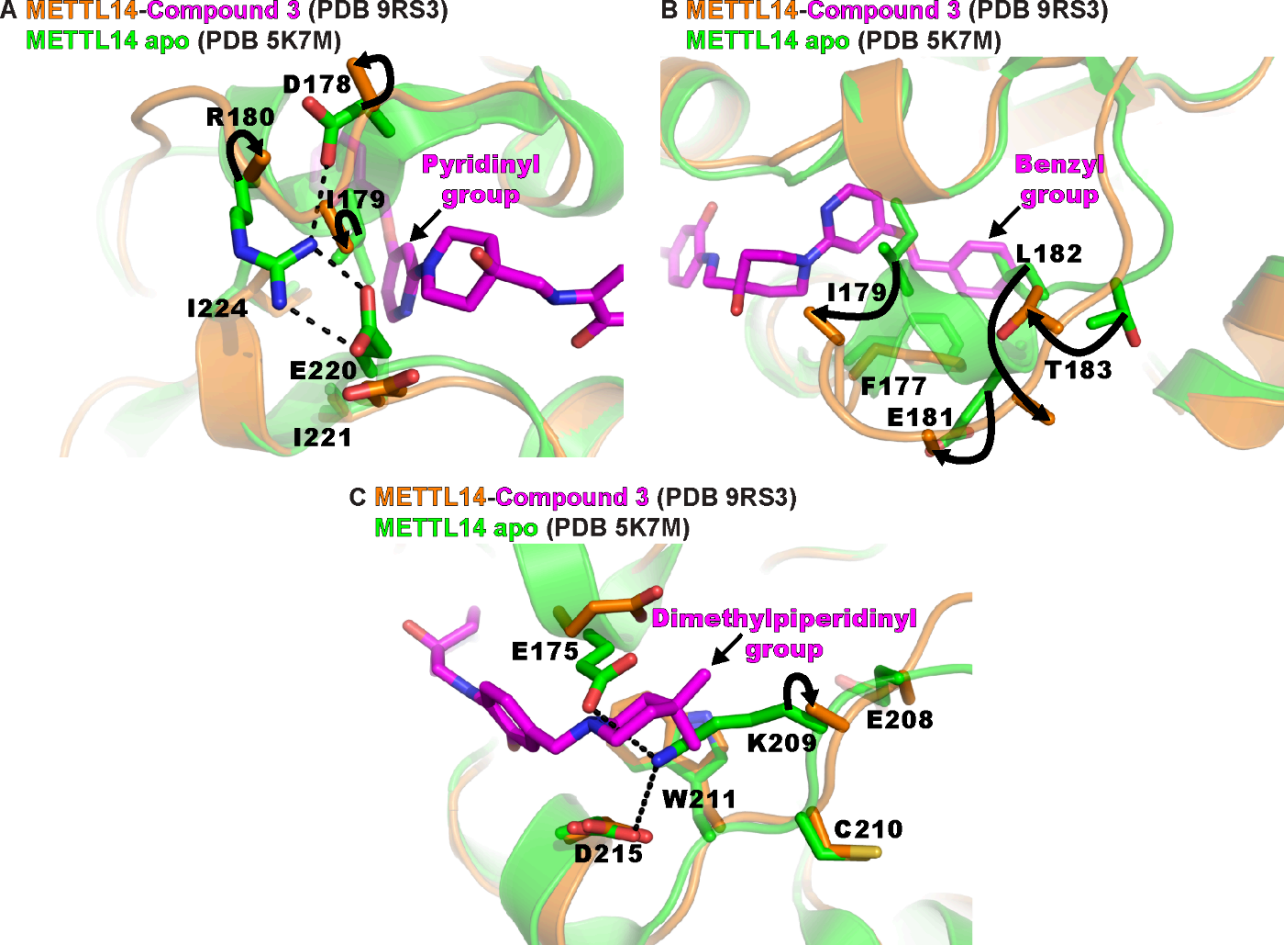
Displacement of METTL14 exosite residues by compound **3**. (A) The structural overlay of METTL14 (backbone shown in cartoon representation, indicated residues as sticks) in the apo state and in the complex with compound **3** (shown as sticks) illustrates the displacement of the Ile179 side chain by the pyridinyl ring of compound **3** and associated conformational changes (black curved arrows). For several METTL14 residues, only the Cβ atoms are resolved in the crystal structure with compound **3** due to lack of electron density, probably due to flexibility. (B) As in (A), for the displacement of Leu182 by the benzyl ring of compound **3**. (C) As in (A), for the displacement of Lys209 by the dimethylpiperidinyl ring of compound **3**. Color coding is indicated at the top of each panel. The moieties of compound **3** are labeled and indicated (straight black arrows). Polar contacts in the crystal structure of apo METTL3–14 are indicated (black dashed lines).

Finally, inhibitor **4** differs from compound **1** by a hydroxyl group in the phenyl ring (Table 1). Its design was inspired by the phenolic OH of compounds **2** and **3**, which acts as hydrogen bond donor for the backbone carbonyl of Asp215 in the METTL14 cryptic pocket (see Figure 2 B,C and Figure S1). Surprisingly, inhibitor **4** binds only to the SAM pocket of METTL3 thanks to an additional hydrogen bond with the backbone carbonyl of Asp395 (Figure S2), while the cryptic pocket of METTL14 is closed. The additional hydrogen bond results in an improved IC_50_ value of 0.15 μM for inhibitor **4** with respect to the IC_50_ value of 0.47 μM for compound **1** (Table 1 and Figure S5). These results indicate that the design of compounds that target only the METTL14 cryptic pocket should take into consideration potential additional interactions not only in the METTL14 exosite but also in the METTL3 SAM pocket.

### Machine Learning and Molecular Dynamics Simulations Are Not Able to Predict the Opening of the Cryptic Pocket of METTL14

Since the METTL14 cryptic pocket is closed in all structures of METTL3–14 published thus far (Figure S1 and Table S1), we decided to assess if Alphafold 3 (AF3) is able to predict its opening (Figures S12–S17 and Table S4).^95^ We also reanalyzed previously published explicit solvent molecular dynamics (MD) simulations of apo METTL3–14 at constant temperature (300 K). The total sampling was 5 μs, i.e., five independent runs of 1 μs each (Figure 5).^93^

**Figure 5:**
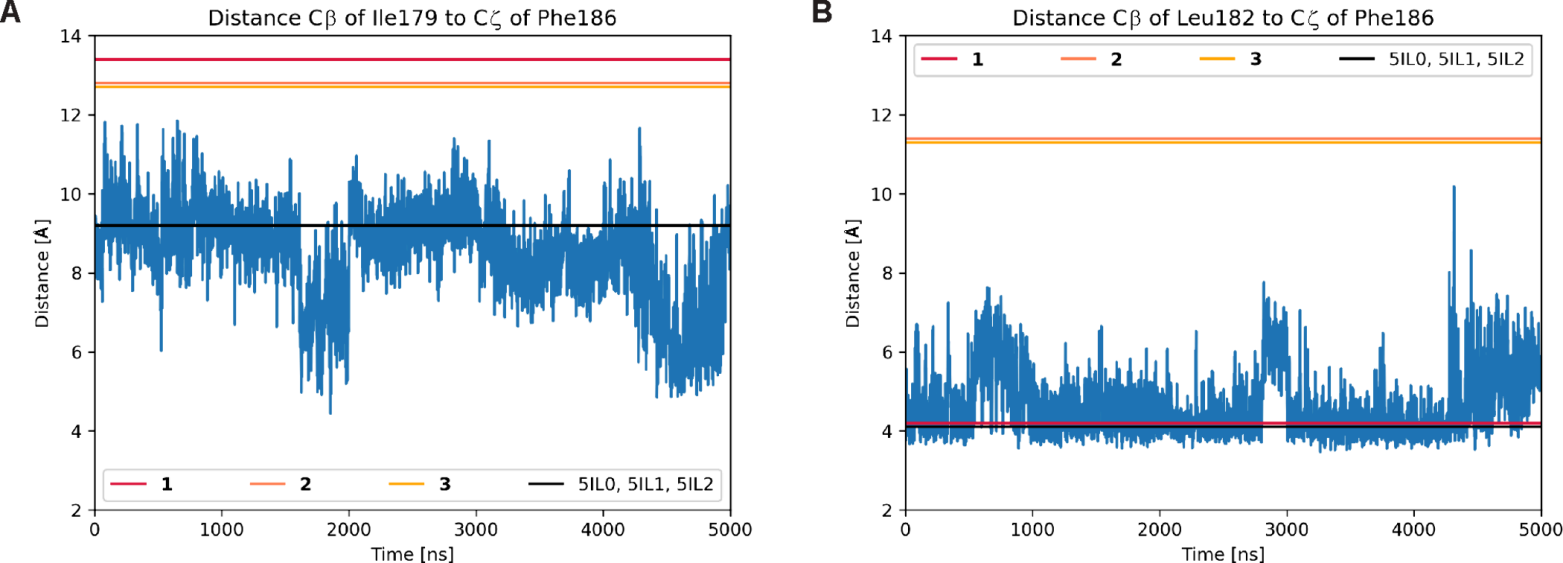
Rigidity of METTL14 residues Ile179 and Leu182 and associated loop during molecular dynamics (MD) simulations of apo METTL3–14. The plots show the time series of two key inter-residue distances (blue line) along five MD trajectories of 1 μs each. The distances between the Phe186 Cζ and the Ile179 Cβ (A) or the Leu182 Cβ (B) report on the orientation of the side chains and the flexibility of METTL14 segment 174–183. The horizontal lines indicate the distance in the crystal structures of the apo and SAM/SAH-bound structures (black) or with the exosite binders (colors with legend in each panel).

In AF3 predictions with only METTL14, all compounds were docked at the side of METTL14 that forms the interface with METTL3 and were thus far away from the exosite. In AF3 predictions with only METTL3 or the METTL3–14 complex and one copy of the compound, all compounds were bound in the METTL3 SAM pocket in similar conformations as in their crystal structures bound to METTL3. This includes even compound **3**, which based on our biochemical and structural analysis does not inhibit nor bind to METTL3 but only binds to the METTL14 cryptic pocket (Figure S16). Hence, the AF3 predictions are likely to be biased by the 57 crystal structures (as of July 29, 2025) that are available of METTL3–14 with inhibitors bound in the METTL3 SAM pocket, all of which have a closed METTL14 exosite (Table S1).

We then decided to further evaluate AF3 by predictions of the METTL3–14 complex with two copies of the exosite binders. One copy of compound **1** was predicted to occupy the METTL3 SAM pocket as expected, and the other copy was bound to the METTL14 exosite, however, without displacing Ile179 and without opening up loop 174–179 (Figure S14). Instead, only the dimethylpiperidine moiety was correctly placed, but the exosite loop was closed and thus the aminopyrimidine moiety pointed toward the solvent. Interestingly, running AF3 with two copies of the potent METTL3 inhibitor UZH2 as a control resulted in similar predictions as for compound **1**, although we have never obtained a crystal structure of UZH2 bound to METTL14 (Figure S13).^67^ Compounds **2** and **3** were not predicted to bind to METTL14 in these AF3 predictions (Figures S15 and S16). Instead, both copies of these compounds were predicted to occupy the SAM pocket in METTL3. The first copy of each of compounds **2** and **3** bound similar to compound **2** in the crystal structure, while the second copy bound close to the first copy. Lastly, compound **4** bound with one copy to METTL3 as expected, while the second copy bound to METTL14, however, not to the exosite, but a site adjacent to it (Figure S17).

The above AF3 predictions were all run using multiple sequence alignment (MSA). We also ran AF3 predictions without MSA, and again AF3 did not predict the opening of the cryptic pocket of METTL14 (data not shown). These results are in line with a recent study that investigated the ability of AlphaFold to predict cryptic pockets.^96^ There, AlphaFold was shown to predict known cryptic pockets in three out of five examples among proteins that were in the training data set. However, AlphaFold predicted only partial openings for proteins whose cryptic pockets were deposited after AlphaFold’s training data were extracted from the PDB.

We also analyzed the distances between the Cβ of Leu182 or Ile179 to the Cζ of Phe186 in previously published MD simulations of apo METTL3–14 (Figure 5).^93^ As mentioned above, Leu182 and Ile179 are in the METTL14 segment that is displaced in the crystal structures with the exosite binders, while Phe186 is on the opposite side of the loop and is rigid. The MD simulations never reached distances as in the crystal structures of the exosite binders; therefore, the METTL14 loop does not open on time scales of 1 μs in MD simulations (Figure 5). This confirms the rigidity of this loop as indicated by its stable conformation throughout the 66 already deposited METTL3–14 crystal structures (as of July 29, 2025, see Figure S1 and Table S1).

## Conclusions

During the optimization of SAM-competitive small-molecule inhibitors of METTL3–14 by medicinal chemistry,^67,93^ protein crystallography unveiled the presence of the inhibitors **1** and **2** in a cryptic pocket of METTL14 in addition to the METTL3 SAM pocket. The opening of the cryptic pocket requires the unfolding of a one-turn 3_10_-helix and substantial displacement of METTL14 segment 174–183 toward solvent. The detailed analysis of crystal structures revealed that the METTL14 cryptic pocket is closed in the apo structure of METTL3–14, and in the structures of METTL3–14 in the complex with SAH, SAM, and 57 SAM-competitive inhibitors. Interestingly, opening of the cryptic pocket was not observed in AlphaFold3 predictions of the complex of METTL3–14 with compounds **1**–**3**, and in multiple 1 μs molecular dynamics simulations of apo METTL3–14. With the structural information on inhibitor **2**, we designed compound **3** that binds only to the METTL14 cryptic pocket while it does not inhibit the catalytic activity of METTL3–14. The lack of allosteric effects from the binding of the exosite ligands to METTL14 is consistent with the distance of about 46 Å between the METTL14 cryptic pocket and the SAM pocket in METTL3. However, whether this cryptic pocket is involved in protein–protein interactions in the cell and whether the METTL14 cryptic pocket binders that we describe here could modulate specific cellular processes require further investigation. Finally, we propose a route for the further development of these compounds into heterobifunctional ligands that, in contrast to SAM-competitive binders, do not inhibit the methyltransferase activity of METTL3 in the cell.

## Materials and Methods

### Chemical Synthesis and Purity

The synthesis of compound **1** is described in ref 67. Compound **4** was synthesized by BioDuro. All other compounds were synthesized by WuXi AppTec. All compounds had purity larger than 95%, except for compound **6** (91.4%). NMR and LC-MS spectra as well as the full synthesis schemes are included in the Supporting Information for all compounds.

### Compound **2** Synthesis

To a solution of 4-iodo-3-methoxy-benzoic acid (5 g, 17.98 mmol, 1 equiv) in DMF (50 mL) were added HATU (10.26 g, 26.97 mmol, 1.5 equiv) and DIEA (4.65 g, 35.97 mmol, 6.26 mL, 2 equiv), and the mixture was stirred at 20 °C for 10 min. Then, 4,4-dimethylpiperidine (2.44 g, 21.58 mmol, 1.2 equiv) was added to the mixture. The mixture was stirred at 20 °C for 1 h. The reaction mixture was diluted with H_2_O (100 mL) and extracted with ethyl acetate (300 mL (100 mL × 3)). The combined organic layers were dried over Na_2_SO_4_, filtered, and concentrated under reduced pressure to give a residue. The residue was purified by column chromatography (SiO_2_, dichloromethane/methanol = 1/0 to 10/1). Compound (4,4-dimethyl-1-piperidyl)-(4-iodo-3-methoxy-phenyl)­met-anone (6 g, 16.08 mmol, 89.40% yield) was obtained as yellow oil.

To a solution of (4,4-dimethyl-1-piperidyl)-(4-iodo-3-methoxyphenyl) methanone (6 g, 16.08 mmol, 1 equiv) in THF (50 mL) was added BH_3_-Me_2_S (10 M, 4.82 mL, 3 equiv) at 0 °C. The mixture was stirred at 70 °C for 2 h. The reaction mixture was quenched by MeOH at 0 °C and stirred at 70 °C for 12 h and then diluted with H_2_O (100 mL) and extracted with ethyl acetate (300 mL (100 mL × 3)). The combined organic layers were washed with brine (200 mL × 2), dried over Na_2_SO_4_, filtered, and concentrated under reduced pressure to give a residue. The residue was purified by column chromatography (SiO_2_, petroleum ether/ethyl acetate = 1/0 to 3/1). Compound 1-[(4-iodo-3-methoxy-phenyl)­methyl]-4,4-dimethyl-piperidine (5.3 g, 14.75 mmol, 91.77% yield) was obtained as yellow oil.

To a solution of 1-[(4-iodo-3-methoxyphenyl)­methyl]-4,4-dimethylpiperidine (2 g, 1 equiv) in 1,4-dioxane (30 mL) were added *tert*-butyl 4-(aminomethyl)-4-hydroxypiperidine-1-carboxylate (1.54 g, 1.2 equiv) and Cs_2_CO_3_ (3.63 g, 2 equiv) and 2-dicyclohexylphosphino-2,6-diisopropoxy-1,1-biphenyl (520 mg, 0.2 equiv) and ruphospalladacyclegen4 (473 mg, 0.1 equiv). The mixture was stirred at 120 °C for 12 h. The reaction mixture was diluted with H_2_O (50 mL) and extracted with ethyl acetate (150 mL (50 mL × 3)). The combined organic layers were washed with brine (200 mL), dried over Na_2_SO_4_, filtered, and concentrated under reduced pressure to give a residue. The residue was purified by column chromatography (SiO_2_, dichloromethane/methanol = 1/0 to 10/1). Compound *tert*-butyl 4-[({4-[(4,4-dimethylpiperidin-1-yl)­methyl]-2-methoxyphenyl}­amino)­methyl]-4-hydroxypiperidine-1-carboxylate (0.8 g) was obtained as yellow oil.

To a solution of *tert*-butyl 4-[({4-[(4,4-dimethylpiperidin-1-yl)­methyl]-2-methoxyphenyl}­amino)­methyl]-4-hydroxypiperidine-1-carboxylate (0.2 g, 1 equiv) in dichloromethane (2.5 mL) was added trifluoroacetic acid (0.5 mL). The mixture was stirred at 20 °C for 12 h. The mixture was concentrated under reduced pressure to give a residue. Compound 4-[({4-[(4,4-dimethylpiperidin-1-yl)­methyl]-2-methoxyphenyl}­amino)­methyl]­piperidin-4-ol was obtained as yellow oil.

To a solution of 4-[({4-[(4,4-dimethylpiperidin-1-yl)­methyl]-2-methoxyphenyl}­amino)­methyl]­piperidin-4-ol (0.2 g, 1 equiv) in MeCN (5 mL) were added *N*-benzyl-6-fluoropyrimidin-4-amine (135 mg, 1.2 equiv) and Na_2_CO_3_ (117 mg, 2 equiv). The mixture was stirred at 70 °C for 12 h. The reaction mixture was diluted with H_2_O (30 mL) and extracted with ethyl acetate (90 mL (30 mL × 3)). The combined organic layers were washed with brine (100 mL), dried over Na_2_SO_4_, filtered, and concentrated under reduced pressure to give a residue. The residue was purified by prep-TLC (SiO_2_, dichloromethane:methanol = 10:1). Compound 1-[6-(benzylamino)­pyrimidin-4-yl]-4-[({4-[(4,4-dimethylpiperidin-1-yl)­methyl]-2-methoxyphenyl}­amino)­methyl]­piperidin-4-ol was obtained as yellow oil.

To a solution of 1-[6-(benzylamino)­pyrimidin-4-yl]-4-[({4-[(4,4-dimethylpiperidin-1-yl)­methyl]-2-methoxyphenyl}­amino)­methyl]­piperidin-4-ol (110 mg, 1 equiv) in dichloromethane (2 mL) was added BBr_3_ (129 mg, 3 equiv) at 0 °C. The mixture was stirred at 20 °C for 12 h. The reaction mixture was quenched by MeOH at 0 °C and concentrated under reduced pressure to give an oil. The oil was purified by prep-HPLC (column: Phenomenex Luna C18 150 × 25 mm × 10 μm; mobile phase: [water­(FA)-ACN]; gradient: 4–34% B over 8 min) and lyophilized. Compound 1-[6-(benzylamino)­pyrimidin-4-yl]-4-[({4-[(4,4-dimethylpiperidin-1-yl)­methyl]-2-hydroxyphenyl}­amino)­methyl]­piperidin-4-ol (18 mg, 95.11% purity, FA salt) was obtained as white solid. Confirmed by HPLC, LC-MS, and H NMR.

### Intermediate **2f** Synthesis

To a solution of 4,6-difluoropyrimidine (1 g, 8.62 mmol, 1 equiv) in i-PrOH (10 mL) were added DIEA (2.23 g, 17.23 mmol, 3.00 mL, 2 equiv) and phenylmethanamine (1.11 g, 10.34 mmol, 1.13 mL, 1.2 equiv). The mixture was stirred at 25 °C for 2 h. The reaction mixture was concentrated under reduced pressure. The crude product was triturated with THF/PE (1:5) at 25 °C for 10 min. Compound *N*-benzyl-6-fluoro-pyrimidin-4-amine (1.5 g, 7.31 mmol, 84.82% yield, 99% purity) was obtained as a white solid.

### Compound **3** Synthesis

To a solution of 4-(benzyloxy)-2-chloropyridine (1 g, 1 equiv) in NMP (5 mL) were added 4-piperidinol (553 mg, 1.2 equiv) and DIEA (1.18 mg, 2 equiv). The mixture was stirred at 120 °C for 12 h. The residue was purified by column chromatography (SiO_2_, petroleum ether/ethyl acetate = 1/0 to 0/1). Compound 1-[4-(benzyloxy)-2-pyridyl]-4-piperidinol (0.8 g, 98% purity) was obtained as yellow oil.

To a solution of 1-[4-(benzyloxy)-2-pyridyl]-4-piperidinol (0.8 g, 1 equiv) in dicholoromethane (8 mL) was added DMP (1.43 g, 1.2 equiv). The mixture was stirred at 20 °C for 2 h. The reaction solution was poured into saturated Na_2_SO_3_, diluted with NaHCO_3_, and extracted with dichloromethane. The combined organic layers were washed with brine, dried over Na_2_SO_4_, filtered, and concentrated under reduced pressure to give product. The residue was purified by column chromatography (SiO_2_, petroleum ether/ethyl acetate = 1/0 to 0/1). Compound 1-[4-(benzyloxy)-2-pyridyl]-4-piperidinone (0.3 g, 92.5% purity) was obtained as white solid.

To a solution of 1-[4-(benzyloxy)-2-pyridyl]-4-piperidinone (250 mg, 1 equiv) in tetrahydrofuran (3 mL) was added magnesium bromide ethenide (581 mg, 5 equiv) at 0 °C. The mixture was stirred at 20 °C for 12 h. The reaction mixture was diluted with H_2_O (20 mL) and extracted with ethyl acetate (60 mL (20 mL × 3)). The combined organic layers were dried over Na_2_SO_4_, filtered, and concentrated under reduced pressure to give a residue. The residue was purified by column chromatography (SiO_2_, petroleum ether/ethyl acetate = 1/0 to 0/1). Compound 1-[4-(benzyloxy)-2-pyridyl]-4-vinyl-4-piperidinol (190 mg, 94.8% purity) was obtained as colorless solid.

To a solution of 1-[4-(benzyloxy)-2-pyridyl]-4-vinyl-4-piperidinol (200 mg, 1 equiv) in dioxane (5 mL) and H_2_O (1 mL) was added OsO_4_ (32.8 mg, 0.2 equiv) at 0 °C, and then NaIO_4_ (686 mg, 5 equiv) was added. The mixture was stirred at 25 °C for 24 h under N_2_ atmosphere. The reaction mixture was quenched by addition of Na_2_SO_3_ aq (10 mL) at 25 °C and then extracted with EtOAc (20 mL (10 mL × 2)). The combined organic layers were washed with H_2_O (5 mL), dried over Na_2_SO_4_, filtered, and concentrated under reduced pressure to give a residue. The crude product was used in the next step without further purification. Compound 1-[4-(benzyloxy)-2-pyridyl]-4-hydroxy-4-piperidinecarbaldehyde (120 mg, crude) was obtained as a yellow oil.

To a solution of 1-[4-(benzyloxy)-2-pyridyl]-4-hydroxy-4-piperidinecarbaldehyde (120 mg, 1 equiv) and 2-amino-5-[(4,4-dimethyl-1-piperidyl)­methyl]­phenol (90 mg, 1 equiv) in MeOH (3 mL) were added acetic acid (2.3 mg, 0.1 equiv) and then sodium boranuidcarbonitrile (48.3 mg, 2 equiv). The mixture was stirred at 25 °C for 12 h. The reaction mixture was quenched by addition of 1 N HCl (3 mL) at 25 °C and then extracted with EtOAc (10 mL (5 mL × 2)). The combined organic layers were washed with H_2_O (5 mL), dried over Na_2_SO_4_, filtered, and concentrated under reduced pressure to give a residue. The residue was purified by prep-HPLC (column: Phenomenex C18 150 × 25 mm × 10 μm; mobile phase: [water­(TFA)-ACN]; gradient: 48–66% B over 10 min) to give compound 1-[4-(benzyloxy)-2-pyridyl]-4-({4-[(4,4-dimethyl-1-piperidyl)­methyl]-2-hydroxyphenylamino}­methyl)-4-piperidinol (76% purity). Then, the desired product (DP) (76% purity) was repurified by prep-HPLC (column: Phenomenex C18 150 × 25 mm × 10 μm; mobile phase: [water­(FA)-ACN]; gradient: 55–72% B over 10 min) to give DP (99.6% purity) and DP (86% purity). Compound 1-[4-(benzyloxy)-2-pyridyl]-4-({4-[(4,4-dimethyl-1-piperidyl)­methyl]-2-hydroxyphenylamino}­methyl)-4-piperidinol (3.6 mg, 99.6% purity) was obtained as a yellow gum and confirmed by LC-MS, HPLC, and H NMR. Then, the DP (86% purity) was repurified by prep-HPLC (column: Phenomenex C18 150 × 25 mm × 10 μm; mobile phase: [water­(FA)-ACN]; gradient: 53–70% B over 10 min). Compound 1-[4-(benzyloxy)-2-pyridyl]-4-({4-[(4,4-dimethyl-1-piperidyl)­methyl]-2-hydroxyphenylamino}­methyl)-4-piperidinol (3.7 mg, 96.7% purity) was obtained as a yellow gum and confirmed by LC-MS, HPLC, and H NMR.

### Intermediate **3f** Synthesis

To a solution of 3-hydroxy-4-nitrobenzaldehyde (0.8 g, 1 equiv) in MeOH (8 mL) were added 4,4-dimethylpiperidine (650 mg, 1.2 equiv) and AcOH (28.7 mg, 0.1 equiv). The mixture was stirred at 20 °C for 30 min. Then, NaBH_3_CN (451 mg, 1.5 equiv) was added into the mixture, and the mixture was stirred at 20 °C for 12 h. The reaction mixture was diluted with H_2_O (30 mL) and extracted with ethyl acetate (90 mL (30 mL × 3)). The combined organic layers were dried over Na_2_SO_4_, filtered, and concentrated under reduced pressure to give a residue. The residue was purified by column chromatography (SiO_2_, petroleum ether/ethyl acetate = 1/0 to 3/1). Compound 5-[(4,4-dimethylpiperidin-1-yl)­methyl]-2-nitrophenol (550 mg, 98.8% purity) was obtained as yellow oil.

To a solution of 5-[(4,4-dimethylpiperidin-1-yl)­methyl]-2-nitrophenol (430 mg, 1 equiv) in ethanol (5 mL) was added palladium (17.3 mg, 0.1 equiv). The mixture was stirred at 20 °C for 4 h. The reaction mixture was diluted with ethanol (20 mL) and concentrated under reduced pressure to give a crude product. Compound 2-amino-5-[(4,4-dimethylpiperidin-1-yl)­methyl]­phenol (0.4 g, 88% purity) was obtained as black oil.

### Compound **4** Synthesis

To a solution of methyl 4-bromo-3-hydroxybenzoate (3.5 g, 15.1 mmol, 1 equiv) in dimethylformamide (50 mL) was added sodium hydride (727 mg, 18.2 mmol, 1.2 equiv) at 0 °C. The mixture was stirred at 0 °C for 0.5 h, and then bromomethoxymethane (2.27 g, 18.2 mmol, 1.2 equiv) was added. The mixture was stirred at 20 °C for 2 h. The mixture was quenched with sat. NH_4_Cl (50 mL) and extracted with EtOAc (100 mL). The organic layer was washed with brine (150 mL × 2), dried, and evaporated to dryness to afford the desired product (4.0 g, 95.98% yield) as colorless oil.

To a solution of methyl 4-bromo-3-methoxyanisate (4 g, 14.5 mmol, 1 equiv) in tetrahydrofuran (60 mL) was added lithium alumanuide (552 mg, 14.5 mmol, 1 equiv) at 0 °C in portions, and the mixture was stirred at 20 °C for 2 h. Then, the mixture was quenched with Na_2_SO_4_·10H_2_O (10 g) and filtered. The filtrate was evaporated to dryness to afford the desired product (3.5 g, ∼65%) as colorless oil.

To a solution of (4-bromo-3-methoxymethoxyphenyl)­methanol (1 g, 2.63 mmol, 1 equiv) and *N*-ethylbis­(isopropyl)­amine (1.36 g, 10.5 mmol, 4 equiv) in dichloromethane (52 mL) was added (mesyloxysulfonyl)­methane (917 mg, 5.26 mmol, 2 equiv). The mixture was stirred at 20 °C for 2 h. Then, the mixture was washed with water (20 mL), dried, and evaporated to dryness to afford the desired product (1.1 g, ∼65%) as colorless oil.

To a solution of 1-bromo-4-[(mesyloxy)­methyl]-2-methoxymethoxybenzene (1.1 g, ∼65%) and 4,4-dimethylpiperidine-hydrogen chloride (329 mg, 2.2 mmol, CAS: 38646-68-3) in acetonitrile (10 mL) was added dipotassium carbonate (1.8 g, 6.6 mmol, 3 equiv), and the mixture was stirred at 20 °C for 2 h. Then, the mixture was filtered, the filtrate was evaporated to dryness, and the residue was purified by flash column (30% EA/PE) to afford the desired product (920 mg, ∼70%) as yellow solid.

To a solution of 1-[(4-bromo-3-methoxymethoxyphenyl)­methyl]-4,4-dimethylpiperidine (1 g, ∼70%), *tert*-butyl 6-oxo-2,5,8-triaza-2-spiro[3.5]­nonanecarboxylate (355 mg, 1.47 mmol), palladium-(1*E*,4*E*)-1,5-diphenyl-1,4-pentadien-3-one (2/3) (67.4 mg, 0.07 mmol, 0.05 equiv), and 2,2′-bis­(diphenylphosphino)-1,1′-binaphthyl (91.7 mg, 0.15 mmol, 0.1 equiv) in toluene (50 mL) was added sodium 2-methyl-2-propanolate (212 mg, 2.21 mmol, 1.5 equiv), and the mixture was stirred under N_2_ at 125 °C for 16 h. Then, the mixture was filtered, and the filtrate was purified by flash column (5% MeOH/DCM) to afford the desired product (250 mg, 32.09% yield) as white solid.

A solution of *tert*-butyl 8-{4-[(4,4-dimethyl-1-piperidyl)­methyl]-2-methoxymethoxyphenyl}-6-oxo-2,5,8-triaza-2-spiro[3.5]­nonanecarboxylate (250 mg, 0.50 mmol) in 4 N HCl/dioxane (6 mL, 4 M) was stirred at 20 °C for 16 h. Then, the mixture was evaporated to dryness to afford the desired product (210 mg, 88.09% yield) as HCl salt.

To a solution of 8-{4-[(4,4-dimethyl-1-piperidyl)­methyl]-2-hydroxyphenyl}-2,5,8-triaza-6-spiro[3.5]­nonanone-hydrogen chloride (60 mg, 0.14 mmol, 1 equiv) and 4,6-difluoropyrimidine (16.1 mg, 0.14 mmol, 1 equiv) in dichloromethane (5 mL) was added TEA (56 mg, 0.56 mmol, 4 equiv) and stirred at 20 °C for 2 h. Then, the mixture was evaporated to dryness to afford the crude product, which was used for the next step.

To a solution of 8-{4-[(4,4-dimethyl-1-piperidyl)­methyl]-2-hydroxyphenyl}-2-(6-fluoro-4-pyrimidinyl)-2,5,8-triaza-6-spiro[3.5]­nonanone (90 mg, 0.14 mmol, 1 equiv) and dicaesium carbonate (174 mg, 0.54 mmol, 3.8 equiv) in NMP (3 mL) was added hydrogen chloride-methylamine (1/1) (60.2 mg, 0.89 mmol, 6.3 equiv), and the mixture was stirred at 140 °C in a sealed tube for 2 h. Then, the mixture was filtered and the filtrate was purified by prep-HPLC to afford the desired product (9 mg, yield: 13.82% yield) as white solid as FA salt.

### Intermediate **4f** Synthesis

To a solution of *tert*-butyl 3-oxoazetidine-1-carboxylate (10 g, 0.06 mol, CAS: 398489-26-4, 1 equiv) and TEA (1.18 g, 0.01 mol, 0.2 equiv) in MeOH (200 mL) was added nitromethane (4.28 g, 0.07 mol, 1.20 equiv). The mixture was stirred at 25 °C for 16 h. Then, the mixture was evaporated to dryness to afford the desired product (11 g, yield: 69%) as yellow oil.

DAST was added (10.0 g, 0.06 mol, 1.2 equiv) dropwise to a stirred solution of *tert*-butyl 3-hydroxy-3-(nitromethyl)­azetidine-1-carboxylate (12 g, 0.05 mmol, 1 equiv) in dry DCM (200 mL) under a nitrogen atmosphere at −78 °C. The cooling bath was removed, and the reaction mixture was stirred for 4 h, cooled to 0 °C, and quenched slowly by the addition of a saturated aqueous NaHCO_3_ solution. The aqueous layer was extracted three times with DCM, washed once with brine, dried over MgSO_4_, filtered, and concentrated under reduced pressure to afford the product (8.8 g, yield: 75.44%) as brown oil.

A solution of *tert*-butyl 3-(nitromethylidene)­azetidine-1-carboxylate (10 g, 0.05 mol) in 7 N NH_3_/MeOH (100 mL) was stirred at 20 °C for 3 h. Then, the mixture was evaporated to dryness to afford the desired product (8.8 g, yield: 73.23%).

To a solution of *tert*-butyl 3-amino-3-(nitromethyl)­azetidine-1-carboxylate (10 g, 0.043 mmol, 1 equiv) and K_2_CO_3_ (11.92 g, 0.09 mol, 2 equiv) in 50% DCM/H_2_O (150 mL) was added dropwise of benzyl chloroformate (8.84 g, 0.05 mmol, 1.2 equiv) at 0 °C. The mixture was stirred at 20 °C for 16 h. The mixture was washed with brine (50 mL × 2). The DCM layer was dried and evaporated to dryness, and the residue was purified by flash column (20% EA/PE) to afford the desired product (7.1 g, yield: 48.33%) as colorless oil.

To a solution of *tert*-butyl 3-{[(benzyloxy)­carbonyl]­amino}-3-(nitromethyl)­azetidine-1-carboxylate (6 g, 16.4 mmol, 1 equiv) and NiCl_2_ (2.13 g, 16.4 mmol, 1 equiv) in MeOH (100 mL) was added NaBH_4_ (3.74 g, 98.4 mmol, 6 equiv) at 0 °C. The mixture was stirred at 20 °C for 2 h. The mixture was quenched with sat. aq. NaHCO_3_ (10 mL) and filtered through diatomite. The filtrate was diluted with EA (300 mL) and washed with brine (300 mL × 2). The EA layer was dried and evaporated to dryness to afford the desired product (4.6 g, yield: 79.27%) as white solid.

To a solution of *tert*-butyl 3-(aminomethyl)-3-{[(benzyloxy)­carbonyl]­amino}­azetidine-1-carboxylate (4.1 g, 12.2 mmol, 1 equiv) and TEA (3.70 g, 36.6 mmol, 3 equiv) in DCM (100 mL) was added dropwise methyl 2-bromoacetate (3.73 g, 24.4 mmol, 2 equiv). The mixture was stirred at 20 °C for 16 h. The mixture was washed with brine (100 mL). The DCM layer was dried and evaporated to dryness, and the residue was purified by flash column (40% EA/PE) to afford the desired product (2.3 g, yield: 44.26%) as colorless oil.

To a solution of *tert*-butyl 3-{[(benzyloxy)­carbonyl]­amino}-3-{[(2-methoxy-2-oxoethyl)­amino]­methyl}­azetidine-1-carboxylate (2.0 g, 4.9 mmol, 1 equiv) and HCOONH_4_ (1.85 g, 29.4 mmol, 5 equiv) in i-PrOH (40 mL) was added Pd/C (200 mg). The mixture was stirred at 75 °C for 4 h. The mixture was filtered, the filtrate was evaporated to dryness, and the residue was recrystallized (100% EA) to afford the desired product (840 mg, yield: 67.35%) as white solid.

### Compound **5** Synthesis

To a solution of 1-[(4-iodo-3-methoxy-phenyl)­methyl]-4,4-dimethyl-piperidine (1.89 g, 5.26 mmol, 1.2 equiv) in dioxane (20 mL) were added *tert*-butyl 4-(aminomethyl)-4-methyl-piperidine-1-carboxylate (1 g, 4.38 mmol, 1 equiv) and Cs_2_CO_3_ (2.85 g, 8.76 mmol, 2 equiv) and RuPhos (408.74 mg, 875.92 μmol, 0.2 equiv) and RuPhos Palladacycle Gen 4 (372.44 mg, 437.96 μmol, 0.1 equiv). The mixture was stirred at 120 °C for 12 h. The reaction mixture was diluted with H_2_O (30 mL) and extracted with ethyl acetate (90 mL (30 mL × 3)). The combined organic layers were dried over Na_2_SO_4_, filtered, and concentrated under reduced pressure to give a residue. The residue was purified by column chromatography (SiO_2_, dichloromethane/methanol = 1/0 to 10/1). Compound *tert*-butyl 4-[[4-[(4,4-dimethyl-1-piperidyl)­methyl]-2-methoxy-anilino]­methyl]-4-methyl-piperidine-1-carboxylate (500 mg, 1.09 mmol, 24.84% yield) was obtained as yellow oil.

To a solution of *tert*-butyl 4-[({4-[(4,4-dimethylpiperidin-1-yl)­methyl]-2-methoxyphenyl}­amino)­methyl]-4-methylpiperidine-1-carboxylate (200 mg, 1 equiv) in DCM (2.5 mL) was added trifluoroacetic acid (0.5 mL). The mixture was stirred at 20 °C for 2 h. The reaction mixture was concentrated under reduced pressure to give a residue. Compound 4-[(4,4-dimethylpiperidin-1-yl)­methyl]-2-methoxy-*N*-[(4-methylpiperidin-4-yl)­methyl]­aniline (0.2 g, 116% yield) was obtained as yellow oil (TFA salt).

To a solution of 4-[(4,4-dimethylpiperidin-1-yl)­methyl]-2-methoxy-*N*-[(4-methylpiperidin-4-yl)­methyl]­aniline (180 mg, 1 equiv) in MeCN (4 mL) were added *N*-benzyl-6-fluoropyrimidin-4-amine (122 mg, 1.2 equiv) and Na_2_CO_3_ (106 mg, 2 equiv). The mixture was stirred at 70 °C for 12 h. The reaction mixture was diluted with H_2_O (20 mL) and extracted with ethyl acetate (60 mL (20 mL × 3)). The combined organic layers were washed with brine (50 mL), dried over Na_2_SO_4_, filtered, and concentrated under reduced pressure to give a residue. The residue was purified by prep-TLC (SiO_2_, petroleum ether:ethyl acetate = 3:1). Compound *N*-benzyl-6-{4-[({4-[(4,4-dimethylpiperidin-1-yl)­methyl]-2-methoxyphenyl}­amino)­methyl]-4-methylpiperidin-1-yl}­pyrimidin-4-amine (0.2 g, 83% purity, 61.09% yield) was obtained as yellow oil.

To a solution of *N*-benzyl-6-{4-[({4-[(4,4-dimethylpiperidin-1-yl)­methyl]-2-methoxyphenyl}­amino)­methyl]-4-methylpiperidin-1-yl}­pyrimidin-4-amine (180 mg, 1 equiv) in dichloromethane (5 mL) was added BBr_3_ (249 mg, 3 equiv) at 0 °C. The mixture was stirred at 20 °C for 12 h. The reaction mixture was quenched by MeOH at 0 °C and concentrated under reduced pressure to give an oil. Half of the fraction was purified by prep-HPLC (column: Phenomenex Luna C18 150 × 25 mm × 10 μm; mobile phase: [water­(10 mM NH_4_HCO_3_)-ACN]; gradient: 39–69% B over 10 min) to yield the desired compound 2-[({1-[6-(benzylamino)­pyrimidin-4-yl]-4-methylpiperidin-4-yl}­methyl)­amino]-5-[(4,4-dimethylpiperidin-1-yl)­methyl]­phenol (20 mg, 87% purity) as yellow gum. Another half of the fraction was purified by prep-HPLC (column: Phenomenex Luna C18 150 × 25 mm × 10 μm; mobile phase: [water­(FA)-ACN]; gradient: 5–35% B over 8 min) to yield the desired compound 2-[({1-[6-(benzylamino)­pyrimidin-4-yl]-4-methylpiperidin-4-yl}­methyl)­amino]-5-[(4,4-dimethylpiperidin-1-yl)­methyl]­phenol (32.09 mg, 95.96% purity, FA salt) as yellow gum, confirmed by H NMR, HPLC, and LC-MS.

### Compound **6** Synthesis

To a solution of 4-(benzyloxy)-2-chloropyridine (0.5 g, 1 equiv) in NMP (5 mL) was added DIEA (588 mg, 2 equiv) and (4-methylpiperidin-4-yl)­methanol (353 mg, 1.2 equiv). The mixture was stirred at 120 °C for 36 h. The reaction mixture was diluted with H_2_O (30 mL) and extracted with ethyl acetate (90 mL (30 mL × 3)). The combined organic layers were washed with brine (100 mL), dried over Na_2_SO_4_, filtered, and concentrated under reduced pressure to give a residue. The residue was purified by column chromatography (SiO_2_, petroleum ether/ethyl acetate = 1/0 to 1/1). Compound {1-[4-(benzyloxy)­pyridin-2-yl]-4-methylpiperidin-4-yl}­methanol (530 mg, 97% purity) was obtained as yellow oil.

To a solution of {1-[4-(benzyloxy)­pyridin-2-yl]-4-methylpiperidin-4-yl}­methanol (0.2 g, 1 equiv) in dicholoromethane (6 mL) was added DMP (326 mg, 1.2 equiv). The mixture was stirred at 20 °C for 1 h. The reaction solution was poured into saturated Na_2_SO_3_, diluted with NaHCO_3_, and extracted with ethyl acetate. The combined organic layers were washed with brine, dried over Na_2_SO_4_, filtered, and concentrated under reduced pressure to give product. Compound 1-[4-(benzyloxy)­pyridin-2-yl]-4-methylpiperidine-4-carbaldehyde (80 mg, 82% purity) was obtained as yellow solid.

To a solution of 1-[4-(benzyloxy)­pyridin-2-yl]-4-methylpiperidine-4-carbaldehyde­(60 mg, 1 equiv) in methanol (2 mL) were added 2-amino-5-[(4,4-dimethylpiperidin-1-yl)­methyl]­phenol (60.4 mg,1 equiv) and acetic acid (7.74 mg, 0.5 equiv); the mixture was stirred at 20 °C for 30 min. After 30 min, boron­(3+) sodium iminomethanide trihydride (33.4 mg, 2 equiv) was added to the mixture. Then, the mixture was stirred at 20 °C for 12 h. The reaction mixture was diluted with H_2_O (10 mL) and extracted with ethyl acetate (20 mL (10 mL × 2)). The combined organic layers were dried over Na_2_SO_4_, filtered, and concentrated under reduced pressure to give a residue. The residue was purified by prep-HPLC (column: Phenomenex Luna C18 150 × 25 mm × 10 μm; mobile phase: [water­(FA)-ACN]; gradient: 14–44% B over 8 min) and lyophilized. 2-[({1-[4-(Benzyloxy)­pyridin-2-yl]-4-methylpiperidin-4-yl}­methyl)­amino]-5-[(4,4-dimethylpiperidin-1-yl)­methyl]­phenol (2.02 mg, 91.4% purity) was obtained as yellow solid. Confirmed by LC-MS, HPLC, and H NMR.

### Compound **7** Synthesis

To a solution of 4-iodo-3-anisic acid (5 g, 1 equiv) in DCM (50 mL) were added 1,1,3,3-tetramethyl-2-(3*H*-1,2,3,4-tetraazainden-3-yl)-3-isoureaium hexafluoridophosphate­(1-) (8.89 g, 1.3 equiv) and *N*-ethylbis­(isopropyl)­amine (4.65 g, 2 equiv). After addition, the mixture was stirred at 25 °C for 30 min, and then 4,4-dimethylpiperidine (2.24 g, 1.1 equiv) was added dropwise at 25 °C. The resulting mixture was stirred at 25 °C for 2 h. The residue was diluted with H_2_O (300 mL) and extracted with EA (300 mL). The combined organic layers were washed with brine (900 mL (300 mL × 3)), dried over Na_2_SO_4_, filtered, and concentrated under reduced pressure to give a residue. The residue was purified by column chromatography (SiO_2_, petroleum ether/ethyl acetate = 0/1 to 3/1). Compound (4,4-dimethyl-1-piperidyl)­(4-iodo-3-methoxyphenyl)­methanone (6.4 g, 80%) was obtained as a yellow solid.

To a solution of (4,4-dimethyl-1-piperidyl)­(4-iodo-3-methoxyphenyl)­methanone (6.4 g,1 equiv) in THF (80 mL) was added BH_3_•Me_2_S (2.87 g, 2.2 equiv) at 0 °C. The resulting mixture was stirred at 70 °C for 12 h. The residue was diluted with H_2_O (300 mL) and extracted with EA (300 mL). The combined organic layers were washed with brine (900 mL (300 mL × 3)), dried over Na_2_SO_4_, filtered, and concentrated under reduced pressure to give a residue. The residue was purified by column chromatography (SiO_2_, petroleum ether/ethyl acetate = 0/1 to 1/1). Compound 1-[(4-iodo-3-methoxyphenyl)­methyl]-4,4-dimethylpiperidine (5.2 g, 100%) was obtained as a yellow solid.

To a solution of 1-[(4-iodo-3-methoxyphenyl)­methyl]-4,4-dimethylpiperidine (5.2 g, 1 equiv) in dioxane (50 mL) were added *tert*-butyl 4-(aminomethyl)-4-hydroxy-1-piperidinecarboxylate (4 g,1.2 equiv), Ruphos Pd G4 (1.23 g, 0.1 equiv), dicyclohexyl­(2’,6’-diisopropoxy-2-biphenylyl)­phosphine (675 mg, 0.1 equiv), and dicaesium carbonate (9.43 g, 2 equiv) at 25 °C. The mixture was stirred at 100 °C for 12 h. The residue was diluted with H_2_O (300 mL) and extracted with EA (300 mL). The combined organic layers were washed with brine (900 mL (300 mL × 3)), dried over Na_2_SO_4_, filtered. and concentrated under reduced pressure to give a residue. The residue was purified by column chromatography (SiO_2_, petroleum ether/ethyl acetate = 0/1 to 2/1). Compound *tert*-butyl 4-({4-[(4,4-dimethyl-1-piperidyl)­methyl]-2-anisidino}­methyl)-4-hydroxy-1-piperidinecarboxylate (900 mg, 88%) was obtained as a yellow solid.

*tert*-Butyl 4-({4-[(4,4-dimethyl-1-piperidyl)­methyl]-2-anisidino}­methyl)-4-hydroxy-1-piperidinecarboxylate (500 mg, 1 equiv) in DCM (10 mL) was mixed with trifluoroacetic acid (2 mL). The mixture was stirred at 25 °C for 1 h. The reaction mixture was concentrated under reduced pressure to remove solvent. Compound 4-({4-[(4,4-dimethyl-1-piperidyl)­methyl]-2-anisidino}­methyl)-4-piperidinol (500 mg) was obtained as a yellow oil.

To a solution of 4-({4-[(4,4-dimethyl-1-piperidyl)­methyl]-2-anisidino}­methyl)-4-piperidinol (480 mg, 1 equiv) in MeCN (10 mL) were added (benzyl)­(4-chloro-1,3,5-triazin-2-yl)­amine (322 mg, 1.1 equiv) and dipotassium carbonate (367 mg, 2 equiv). The mixture was stirred at 70 °C for 12 h. The residue was diluted with H_2_O (30 mL) and extracted with EA (30 mL). The combined organic layers were washed with brine (150 mL (50 mL × 3)), dried over Na_2_SO_4_, filtered, and concentrated under reduced pressure to give a residue. The residue was purified by prep-HPLC (column: Waters xbridge 150 × 25 mm 10 μm; mobile phase: water­(NH_4_HCO_3_)-ACN; B%: 10–41%, 10 min). Compound 1-(6-benzylamino-1,3,5-triazin-2-yl)-4-({4-[(4,4-dimethyl-1-piperidyl)­methyl]-2-anisidino}­methyl)-4-piperidinol (198 mg, 80%) was obtained as a yellow solid confirmed by LC-MS, HPLC, and H NMR.

### Intermediate **7f** Synthesis

To a solution of 2,4-dichloro-1,3,5-triazine (1 g, 1 equiv) in NMP (10 mL) was benzylamine (857 mg, 1.2 equiv) and *N*-ethylbis­(isopropyl)­amine (1.72 g, 2 equiv). The mixture was stirred at 100 °C for 12 h. The residue was diluted with H_2_O (50 mL) and extracted with EA (50 mL). The combined organic layers were washed with brine (300 mL (100 mL × 3)), dried over Na_2_SO_4_, filtered, and concentrated under reduced pressure to give a residue. The residue was purified by column chromatography (SiO_2_, petroleum ether/ethyl acetate = 0/1 to 1/1). Compound (benzyl)­(4-chloro-1,3,5-triazin-2-yl)­amine (230 mg, 80%) was obtained as a yellow solid.

### Compound **8** Synthesis

To a solution of 1-(6-benzylamino-1,3,5-triazin-2-yl)-4-({4-[(4,4-dimethyl-1-piperidyl)­methyl]-2-anisidino}­methyl)-4-piperidinol (100 mg, 1 equiv) in DCM (5 mL) was added tribromoborane (138 mg,3 equiv) at 0 °C. The mixture was stirred at 25 °C for 12 h. The residue was diluted with H_2_O (30 mL) and extracted with EA (30 mL). The combined organic layers were washed with brine (150 mL (50 mL × 3)), dried over Na_2_SO_4_, filtered, and concentrated under reduced pressure to give a residue. The residue was purified by prep-HPLC (column: Waters XBridge 150 × 25 mm 10 μm; mobile phase: water­(NH_4_HCO_3_)-ACN; B%: 8–38%, 10 min). Compound 1-(6-benzylamino-1,3,5-triazin-2-yl)-4-({4-[(4,4-dimethyl-1-piperidyl)­methyl]-2-hydroxyphenylamino}­methyl)-4-piperidinol (5.14 mg, 97.5%) was obtained as a yellow solid confirmed by LC-MS, HPLC, and H NMR.

### METTL3–14 Expression and Purification

For determining the half maximal inhibitory concentration (IC_50_) with the full-length complex and for crystallization studies with the truncated complex METTL3^MTD^:METTL14^MTD^ containing just the methyltransferase domains (MTD) of METTL3 (residues 354–580) and METTL14 (residues 107–395), the recombinant complex constructs were expressed using the baculovirus/Sf9 insect cell expression system and purified as described previously.^97^ Briefly, proteins were expressed from the baculovirus vector pFastBacDual-StrepII-GFP-TEV-METTL3-His-TEV-METTL14 or pFastBacDual-METTL3(354–580)-His-TEV-METTL14(107–395). Recombinant baculoviruses were generated using the Bac-to-Bac system. For protein expression, suspension cultures of Sf9 cells in Sf-90 II SFM medium (Thermo Fisher) were infected at a density of 2 × 10^6^ mL^–1^. Cells were harvested 72 h post infection, resuspended in buffer A (50 mM Tris–HCl pH 8.0, 500 mM NaCl) supplemented with Protease Inhibitor Cocktail (Roche Diagnostics GmbH, Germany), phenylmethylsulfonyl fluoride, and Salt Active Nuclease (Merck), and lysed by sonication. The protein complex was purified by Ni-affinity chromatography on a 5 mL HisTrap HP column (Cytiva) equilibrated and washed with buffer A and eluted with 250 mM imidazole. The full-length complex was further purified by Strep-tag purification using a 5 mL StrepTrap XT column (Cytiva), equilibrated and washed with buffer A, and eluted with 50 mM biotin. The affinity tags were removed by digestion with TEV protease overnight at 4 °C, followed by further purification by size exclusion chromatography using a Superdex 200 Increase 10/300 GL column (Cytiva) in 20 mM Tris–HCl, pH 8.0, 200 mM KCl. The proteins were concentrated, flash-frozen in liquid nitrogen, and stored at −80 °C until further use.

### Protein Crystallization

The SAH (*S*-adenosyl-l-homocysteine)-bound protein crystals of METTL3^MTD^:METTL14^MTD^ were obtained using the hanging drop vapor diffusion method by mixing 1 μL of protein complex solution at ∼5 mg/mL with 1 μL of reservoir solution containing 20% PEG-3350 and 200–400 mM Mg-acetate (optimized for maximum crystal size). The exosite binders were dissolved to final concentrations of 1–200 mM in DMSO. Complex structures were solved by soaking the compounds into METTL3–14/SAH crystals. First, 1 μL of the exosite binder dissolved in DMSO was left overnight to evaporate the solvent at room temperature. The next day, 1 μL of cryo-protectant solution containing 30% PEG-3350 and 200–400 mM Mg-acetate (as above), with or without 100 mM Tris–HCl at pH 8.0, was added on top of the dried compound stamp. One METTL3-14/SAH crystal was then transferred into the mother liquor over the target compound stamp. After 16 h of incubation at 22 °C, the crystals were harvested and flash-frozen in liquid nitrogen.

### Data Collection and Structure Solution

Diffraction data were collected at the PXIII beamline at the Swiss Light Source (SLS) of the Paul Scherrer Institute (PSI, Villigen, Switzerland). Data were processed using XDS.^98^ The crystal structures were solved by molecular replacement by employing the Protein Data Bank (PDB) 5L6D structure as the search model in the Phaser program (Phenix package).^99^ Crystallographic models were constructed through iterative cycles of manual model building with COOT and refinement with Phenix.refine.^100–103^ Figures were made using PyMOL (The PyMOL Molecular Graphics System, Version 3.0 Schrödinger, LLC). Plots of electrostatic surface potential were generated using the APBS tool in PyMOL.^104^

### m^6^A-RNA Reader-Based HTRF Assay

The inhibitory potencies of the exosite binders for METTL3 were quantified by a homogeneous time-resolved fluorescence (HTRF)-based enzyme assay as previously described.^94^ Briefly, the level of m^6^A in an RNA substrate after the reaction catalyzed by METTL3–14 was quantified by measuring specific binding to the m^6^A reader domain of YTHDC1 (residues 345–509) by HTRF. Exosite binders that inhibit METTL3 decrease the m^6^A level and thus reduce the HTRF signal. Dose–response curves of titrations with the exosite binders were plotted in OriginLab 2018 and fitted with nonlinear regression “log­(inhibitor) vs. normalized response with variable slope” from which IC_50_ values were determined.

### DNA-Based Fluorescence Polarization Binding Assay for the m^6^A-RNA Reader YTHDC1

In a control experiment (see Supporting Information), we tested that the compounds do not bind to the m^6^A reader YTHDC1 which is used in the detection step of the HTRF assay. An in-house established fluorescence polarization (FP) assay was employed. It makes use of a fluorescently labeled 17-mer single-stranded DNA probe containing an N^6^-methyladenosine modification.^105^ The protein concentration corresponding to the EC_60_ value for YTHDC1 was selected. Compounds **1**–**4** were serially diluted 3-fold from 1000 to 0.04 μM and incubated with the protein–probe complex. The value of FP was measured after 1 h of incubation at 25 °C.

### Thermal Shift Assay

The METTL3–14 containing only the methyltransferase domains of each protein or the full-length complex was buffered in 25 mM HEPES (pH 7.4) or 20 mM Tris (pH 8.0), respectively, and assayed in a 96-well plate at a final concentration of 2 μM in a 20 μL volume. The SYPRO Orange dye was added as a fluorescence probe at a dilution of 1:1000. The compound concentrations tested were starting from 1000 μM and a 2-fold serial dilution thereof. The temperature was raised with a step of 0.6 °C/s starting from 20 to 85 °C, and fluorescence readings were taken at each interval in a LightCycler 480 (Roche). The reported values (Δ*T*_m_) were calculated as the difference between the melting temperatures of an individual sample and the average of the reference wells (containing the protein and DMSO only) in the same plate. The DMSO concentration was kept at 2% (v/v).

### Cell Viability Assay

Cells were seeded in white clear-bottom 96-well plates at a density of 6–20 × 10^3^ cells/well in 50 μL of the complete RPMI medium and treated with 50 μL of increasing concentrations of the indicated compounds dissolved in DMSO (final concentration of compounds 0.01–10 μM) or DMSO only as a negative control (0.01% (v/v)) and incubated for 72 h at 37 °C with 5% CO_2_. Cell viability was determined using a CellTiter-Glo luminescent cell viability assay (Promega) based on the detection of ATP according to the manufacturer’s instructions. 100 μL of the reagent was added to each well and incubated for 10 min at room temperature. The luminescence was recorded using a Tecan Infinite 3046 M1000 microplate reader from the top. The background luminescence value was obtained from wells containing the CellTiter-Glo reagent and medium without cells. The resulting data was analyzed in GraphPad Prism 9.

### Cellular Thermal Shift Assay

One million of PC-3 cells were suspended in 100 μL of PBS (10010023, Thermo Fisher Scientific) containing 2× protease inhibitor cocktail (11697498001, Roche), for each condition tested. Cells were incubated with compounds or DMSO control (1% (v/v)) for 1 h at 37 °C. They were then heat treated at the indicated temperatures in a thermoblock for 3 min, followed by cooling to room temperature (3 min). Next, samples were lysed by three freeze–thaw cycles in liquid nitrogen and centrifuged at 16,000*g* for 30 min, 4 °C. Equal volumes of control and tested samples (12 μL) were analyzed by Western blot. The changes in the amount of METTL3 and METTL14 protein (after normalization for β-actin and/or GAPDH) were monitored by performing densitometry in Image Studio Lite software and analyzed in GraphPad Prism 9.

### Quantification of m^6^A/A Ratio in Polyadenylated RNA by UPLC-MS/MS Analysis

UPLC-MS/MS was performed as previously described.^77^ Briefly, MOLM-13 cells were seeded into six-well plates at a density of 1 × 10^6^ cells/mL in 2 mL of complete RPMI medium. Cells were treated with the indicated concentrations of compounds or DMSO control (final concentration 0.5% (v/v)) for 16 h. Following the incubation, cells were collected by centrifugation and washed once with PBS, and total RNA was extracted using 0.5 mL of GENEzol (Geneaid) reagent according to the manufacturer’s instructions. The final volume of 50 μL of total RNA extract was subjected to two rounds of purification using 25 μL of Sera-Mag magnetic oligo­(dT) particles (Cytiva) per sample. The polyadenylated RNA was eluted with nuclease-free water in a final volume of 25 μL, and its concentration was determined using a NanoDrop 2000 spectrophotometer (Thermo Scientific). One hundred nanograms of mRNA was digested to nucleosides and dephosphorylated in a one-pot reaction using 0.5 μL of nucleoside digestion mix (M0649S, NEB) in 20 μL of total reaction volume for 16 h at 37 °C. The samples were used for UPLC-MS/MS analysis without further purification steps. The data were plotted by using GraphPad Prism 9.

## Supplementary Material



## Notes

Structural information, detailed illustrations of the interactions between the ligands and METTL3, electron density maps of the active site/exosite of METTL3/METTL14 bound to the ligands, dose–response curves of the METTL3–14 enzymatic TR-FRET assay with ligands, data from the cellular assays, in vitro thermal shift assay, crystal titrations with different compound concentrations, Alphafold3 (AF3) predictions of METTL3–14 with ligands, and chemical synthesis schemes and NMR and LC-MS analysis for all compounds; PDB IDs of all METTL3–14 structures without exosite binders published thus far (as of July 29, 2025); compounds **5**–**8**; results of the METTL3–14 crystal titrations with compounds **1**–**3**; interface predicted template modeling (ipTM) scores from the AF3 predictions; and crystallography data collection and refinement statistics of the new structures published within this article (PDF)

## †

Michael J. Hartshorn - Isohelio Limited, Great Chesterford, Essex, CB10 1PF, United Kingdom

## Abbreviations

METTL14: methyltransferase-like protein 14
METTL3: methyltransferase-like protein 3
m6A, N(6): methyladenosine
mRNA: messenger ribonucleic acid
