# Supplementary material for: Ligand-Induced Opening of a Cryptic Pocket in METTL14
Source: ACS Bio Med Chem Au. 2026 Mar 10;6(2):130–44. doi: 10.1021/acsbiomedchemau.5c00184 (PMC13087807; doi:10.1021/acsbiomedchemau.5c00184)

Supporting information for

Ligand-induced opening of a cryptic pocket in METTL14

Ivan Corbeski*, Rajiv Kumar Bedi, Christian M. Matter, Fiona Stamm, Elena Bochenkova, Marcin Herok, Michael J. Hartshorn†, Amedeo Caflisch*

Department of Biochemistry, University of Zurich, Zurich CH-8057, Switzerland

* Corresponding Authors

Amedeo Caflisch – **Email:** caflisch@bioc.uzh.ch

Ivan Corbeski – **Email:** i.corbeski@bioc.uzh.ch

† Current address

Isohelio Limited, Great Chesterford, Essex, CB10 1PF, United Kingdom.

Table of Contents

Supplementary Figures 3
Supplementary Tables.. 18
Chemistry: chemical synthesis schemes, NMR spectra, and LC-MS analysis of all compounds 22

Supplementary Figures

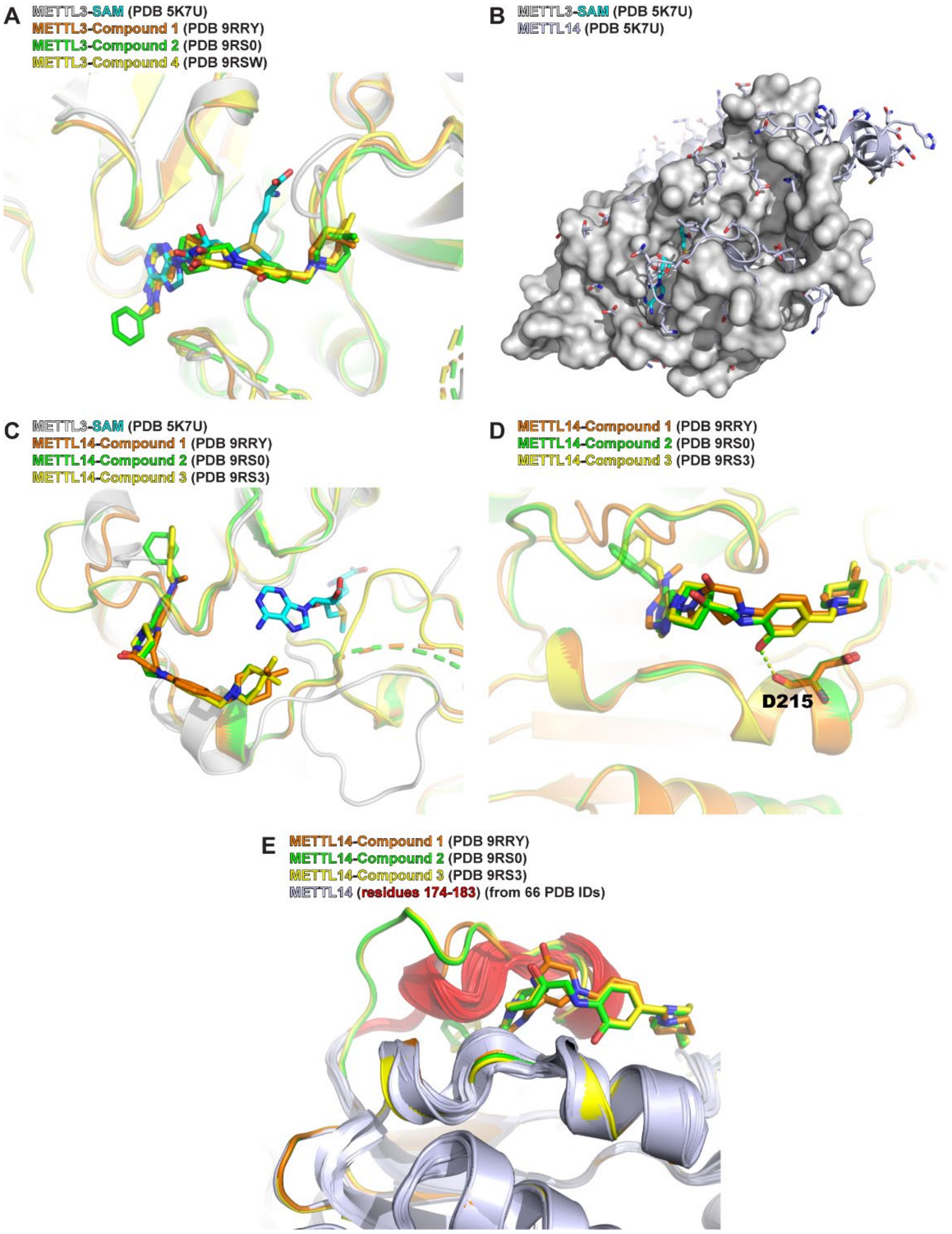

Figure S1. Compounds bind in the METTL3 SAM binding site and in a cryptic pocket of METTL14. (A) Overlay of compounds **1**, **2**, and **4** (shown as sticks) bound to METTL3 (backbone shown in cartoon representation) and its holo state bound to SAM (shown as sticks) showing the SAM-competitive binding mode of the compounds. (B) Superposition of METTL3 (shown as surface) in its holo state bound to SAM (shown as sticks) and METTL14 (backbone shown in cartoon representation, side chains shown as sticks) showing the blockage of the putative SAM binding site in METTL14. (C) Superposition of METTL14 and METTL3 (backbone shown in cartoon representation) with exosite binders and SAM (shown as sticks), respectively, showing that the exosite binders bind to a cryptic pocket adjacent to the putative SAM binding site of METTL14. (D) Overlay of METTL14 (backbone shown in cartoon representation, indicated residue as sticks) bound to compounds **1**, **2**, and **3** (shown as sticks) showing their similar binding mode. A hydrogen bond with compounds **2** and **3** is shown (dashed lines). (E) As in (D), with the three METTL14-exosite binder complexes overlayed with METTL14 from 66 crystal structures of METTL3-14 without exosite binders (Supplementary Table S1), illustrating the conformational stability and opening of the METTL14 segment 174-183 without and with exosite binders, respectively. Color coding is indicated at the top of each panel.

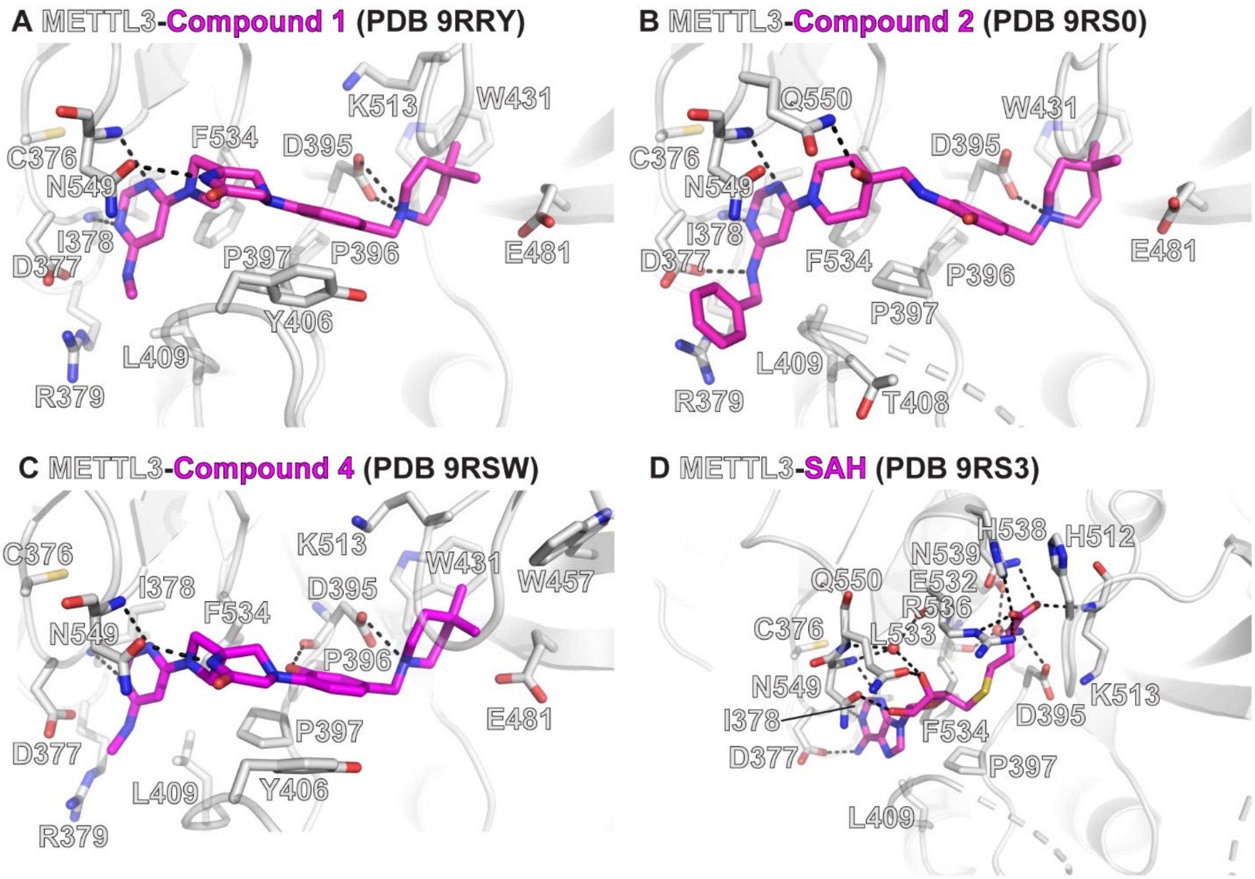

Figure S2. Crystal structures of METTL3-14 bound with compounds. Close-up view of the METTL3 active site (backbone shown in cartoon representation, residues forming the binding pocket environment as sticks, waters as red spheres) bound to compound **1** (A), compound **2** (B), compound **4** (C), and SAH from crystallization trials with compound **3** (D). Black dashes indicate polar contacts in the crystal structure.

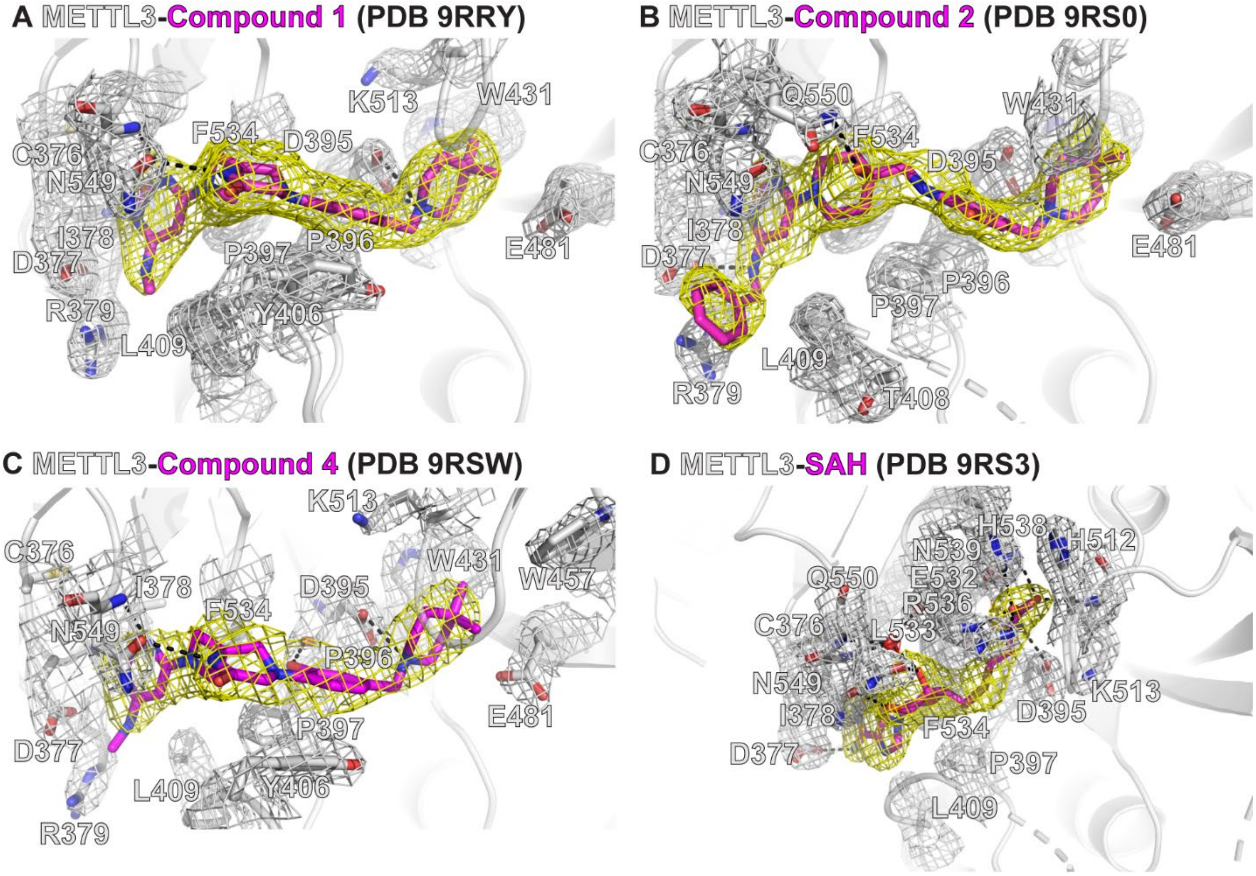

Figure S3. Electron density maps in the SAM-pocket of METTL3 of crystal structures of METTL3-14 bound with compounds. Panels as in Supplementary Figure S2, shown with electron densities for the compounds (yellow mesh, contoured at 0.7 sigma) and METTL3 side chains and waters (grey and red mesh, respectively, contoured at 1.0 sigma).

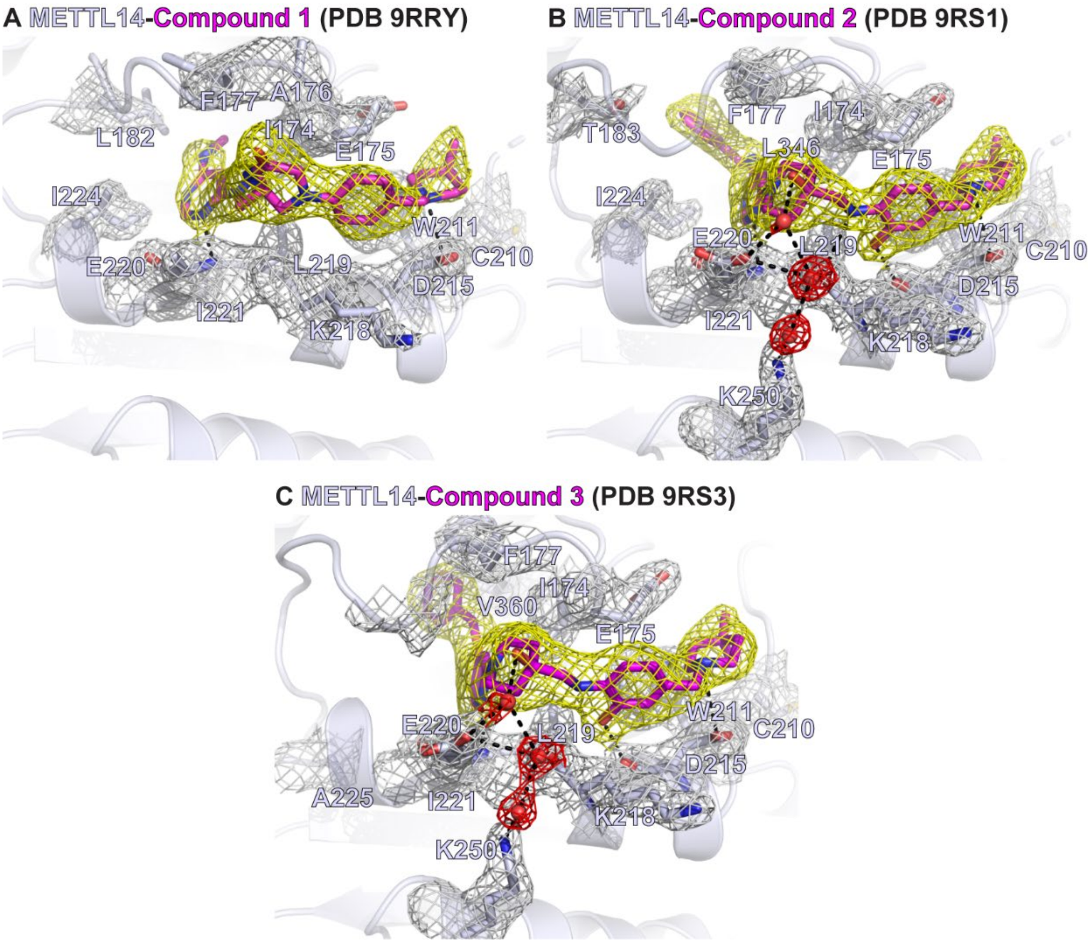

Figure S4. Electron density maps in the cryptic pocket of METTL14 of crystal structures of METTL3-14 bound with compounds. Panels as in main text Figure 2, shown with electron densities for the compounds (yellow mesh, contoured at 0.7 sigma) and METTL14 side chains and waters (grey and red mesh, respectively, contoured at 1.0 sigma).

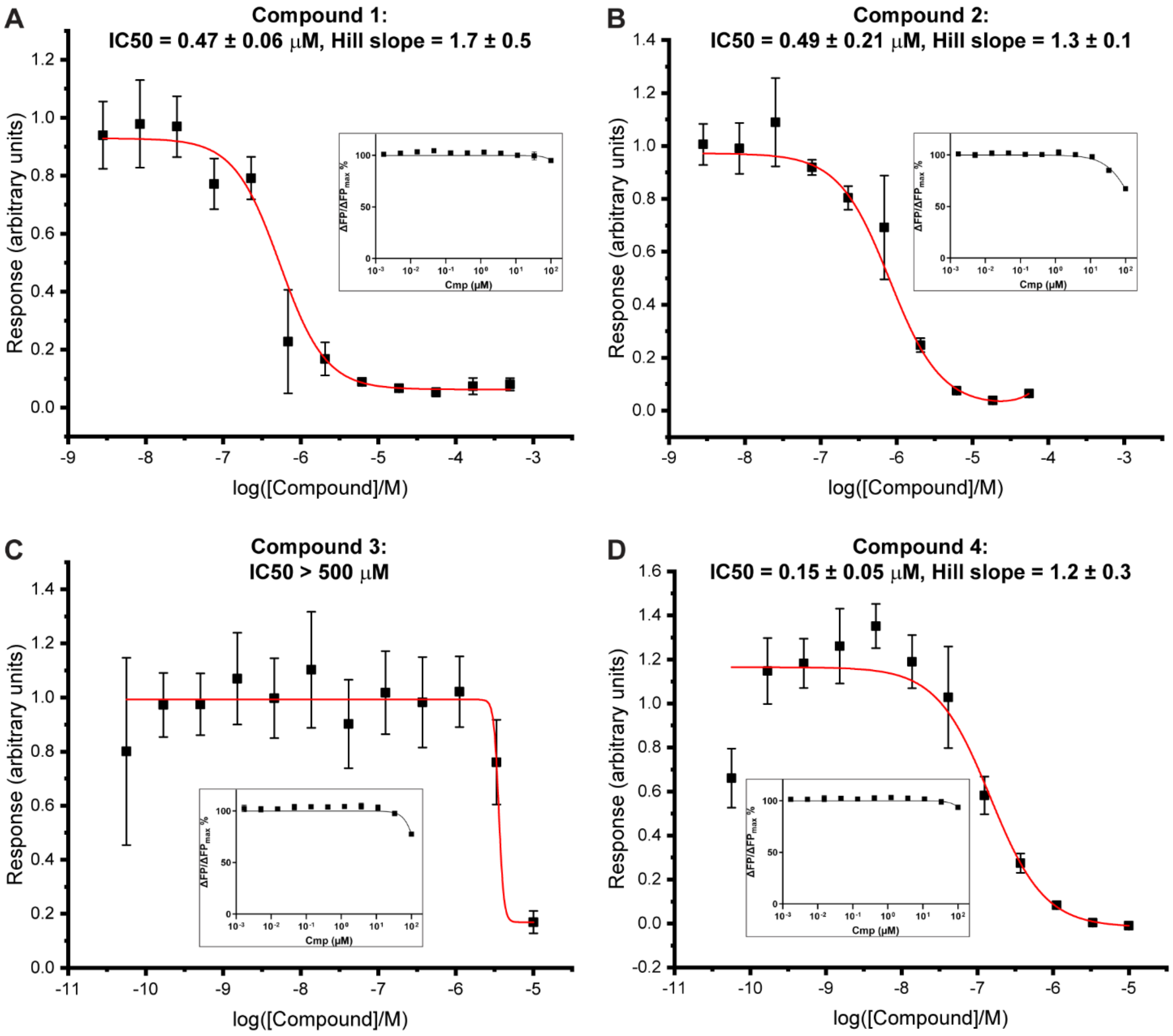

Figure S5. METTL14 exosite binders show dose-dependent inhibitory effects in a time resolved-Förster resonance energy transfer (TR-FRET)-based enzymatic assay of METTL3-14, except for the optimized compound **3**. Dose-response curves derived from the reader-based TR-FRET inhibition assay on METTL3-14 (mean ± standard deviation, n = 3 technical replicates) for METTL14 exosite binders: compound **1** (A), compound **2** (B), compound **3** (C), and compound **4** (D). IC_{50} and Hill slope values were obtained from fits with nonlinear regression 'log(inhibitor) vs. normalized response with variable slope' and are given at the top of each curve. The insets in each panel show the result of the control experiment: the compounds were tested in a fluorescence polarization (FP) binding assay with the m^6A reader YTHDC1 which is used in the detection step of the TR-FRET-based enzymatic assay of METTL3-14. The FP assay shows that the compounds do not bind to YTHDC1.

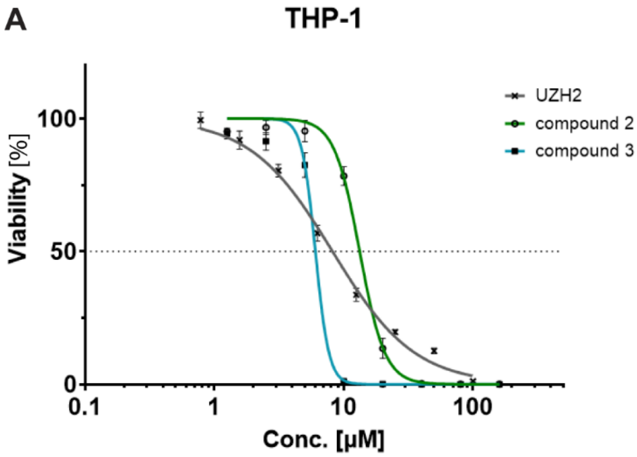

	UZH2	compound 2	compound 3
GI50	8.176	13.26	6.029
HillSlope	-1.369	-4.448	-8.259

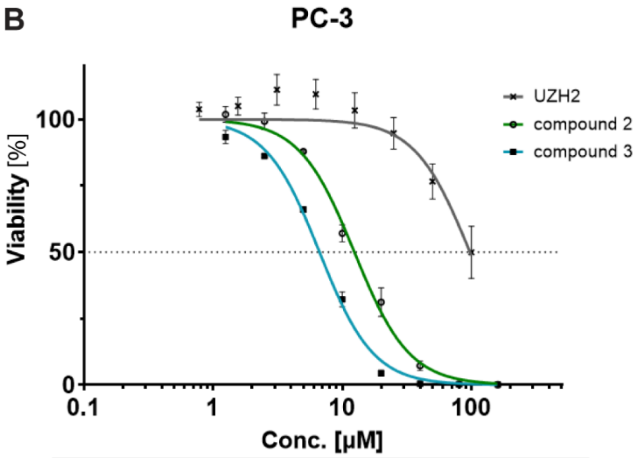

	UZH2	compound 2	compound 3
GI50	97.37	12.42	6.671
HillSlope	-2.040	-2.043	-2.090

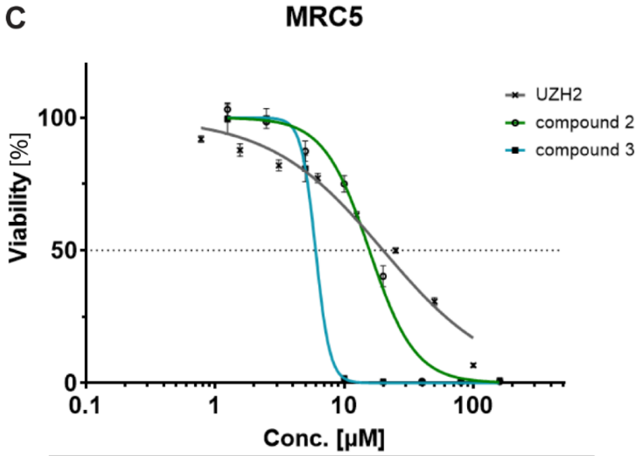

	UZH2	compound 2	compound 3
GI50	20.02	15.67	6.009
HillSlope	-0.9886	-2.458	-7.832

Figure S6. Cellular characterization of compounds **2** and **3** in cell viability assays. Compounds **2** and **3** and the METTL3 catalytic inhibitor UZH2 were tested in AML cell line THP-1 (A), prostate cancer cell line PC-3 (B), and non-cancerous cell line MRC-5 (C). The dashed line represents the 50% cell viability level. Data points/error bars represent averages/standard deviations from triplicate measurements. GI_{50} and Hill slope values were obtained from fits with nonlinear regression 'log(inhibitor) vs. normalized response with variable slope' and are given at the bottom of each panel.

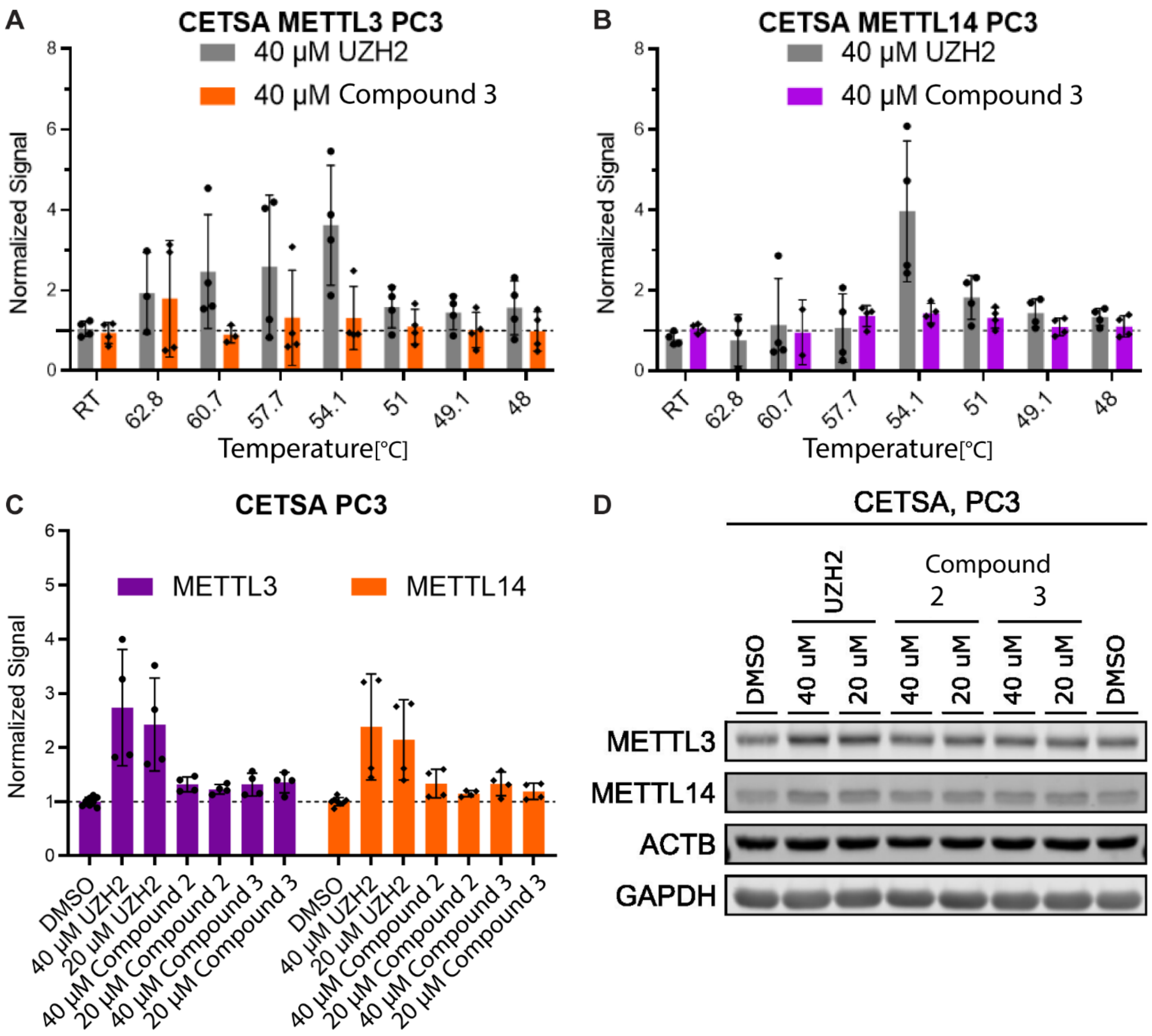

Figure S7. Characterization of compounds **2** and **3** by the cellular thermal shift assay (CETSA) in the prostate cancer cell line PC-3. The stabilization of METTL3 (A) and METTL14 (B) was first measured at different temperature values (x-axis). (C) Stabilization of METTL3 and METTL14 at 54°C with different concentrations of compounds **2** and **3**. The dashed line (y = 1) represents the protein level of the DMSO control which is used for normalization. Columns/error bars represent averages/standard deviations. (D) Representative Western Blot of a CETSA experiment that was used to quantify protein stabilization. The METTL3 catalytic inhibitor UZH2 was used as control.

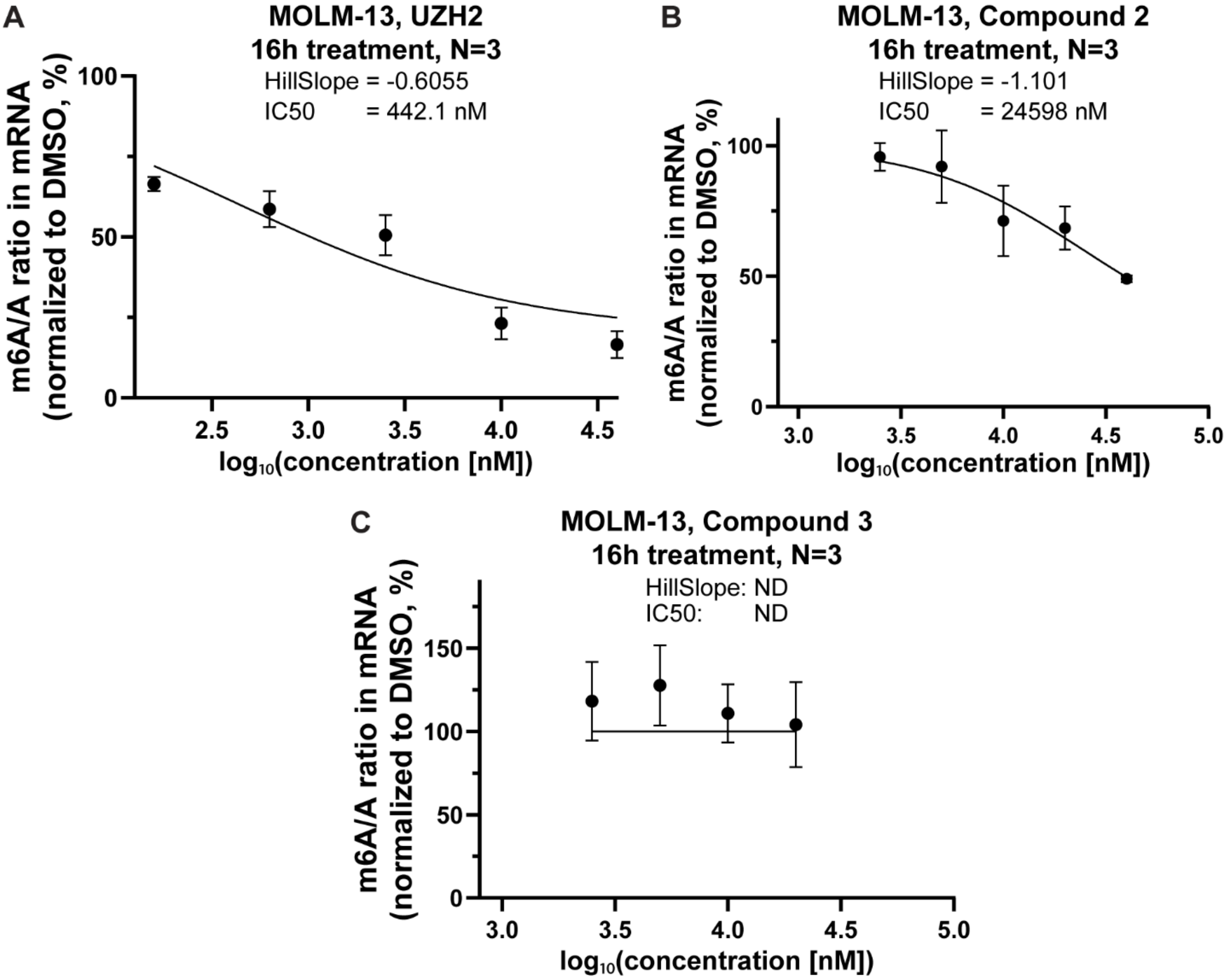

Figure S8. LC-MS measurements of the reduction of the m^6A/A ratio in mRNA. The METTL3 catalytic inhibitor UZH2 (A), compound **2** (B), and compound **3** (C) were tested in the AML cell line MOLM-13. Data points/error bars represent averages/standard deviations from triplicate measurements. IC_{50} and Hill slope values were obtained from fits with nonlinear regression 'log(inhibitor) vs. normalised response with variable slope' with the top and bottom fixed at 100% and 20%, respectively, and are given at the top of each panel.

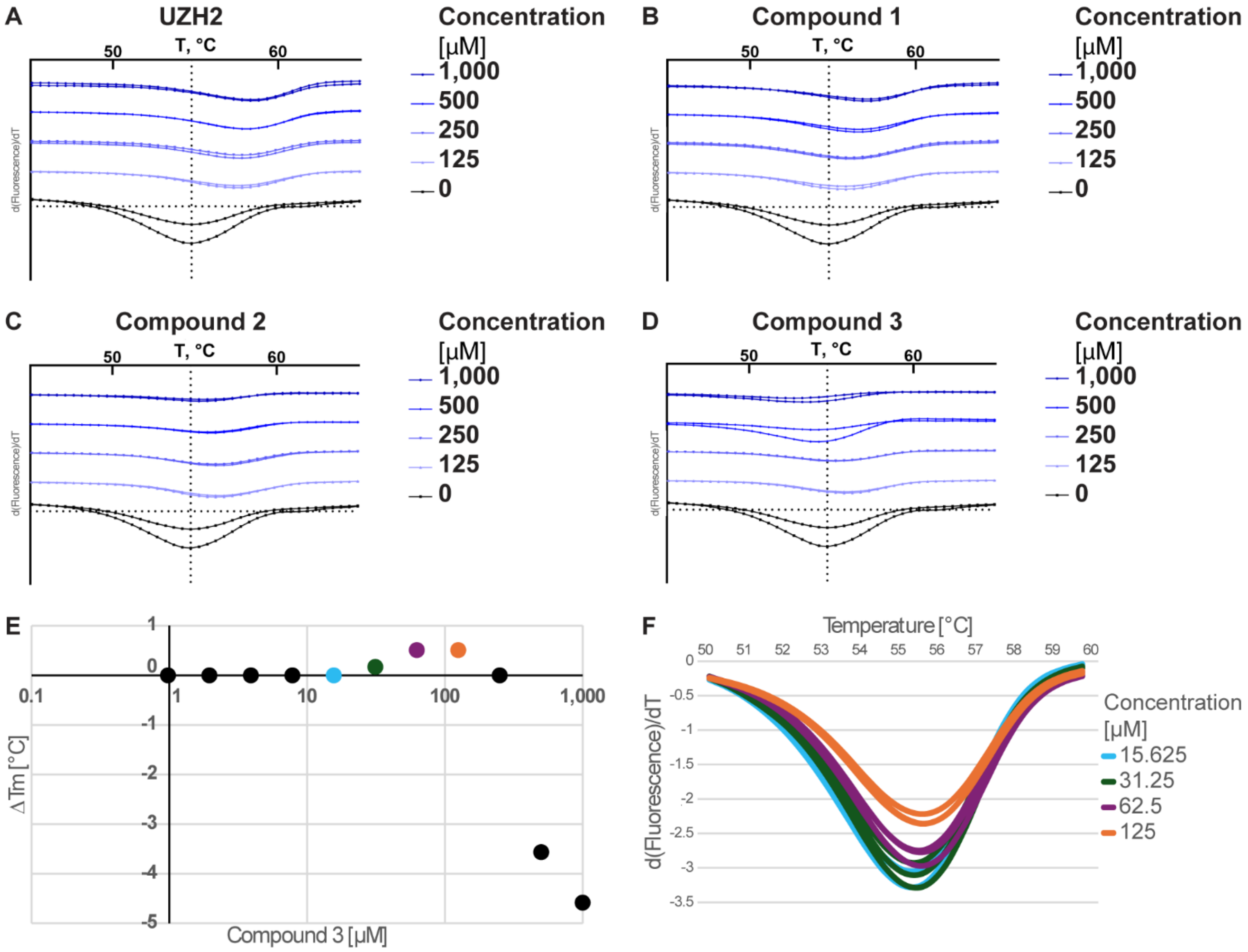

Figure S9. Thermal shift assay. (A-D) Shown are the first derivatives of the melting curves of METTL3(MTD)-14(MTD) (containing only the methyltransferase domains) in the presence of different concentrations of UZH2 (A), compound **1** (B), compound **2** (C), and compound **3** (D). Note that for compound **3** (D), there is a positive shift, indicating stabilization, at 125 µM, whereas the protein is destabilized at higher concentrations. (E) Dose response of the thermal shift (data points shown as circles) of METTL3-14 full-length at different concentrations of compound **3** showing stabilization in a narrow window of compound **3** concentrations and destabilization at very high compound **3** concentrations. Melting temperatures were determined as the temperatures at which the first derivatives of the melting curves reached their minimum. Thermal shifts were determined as the differences in melting temperatures to the DMSO control. (F) First derivatives of the melting curves indicated with colored circles in panel (E) showing the small but clear thermal shift at higher compound **3** concentrations.

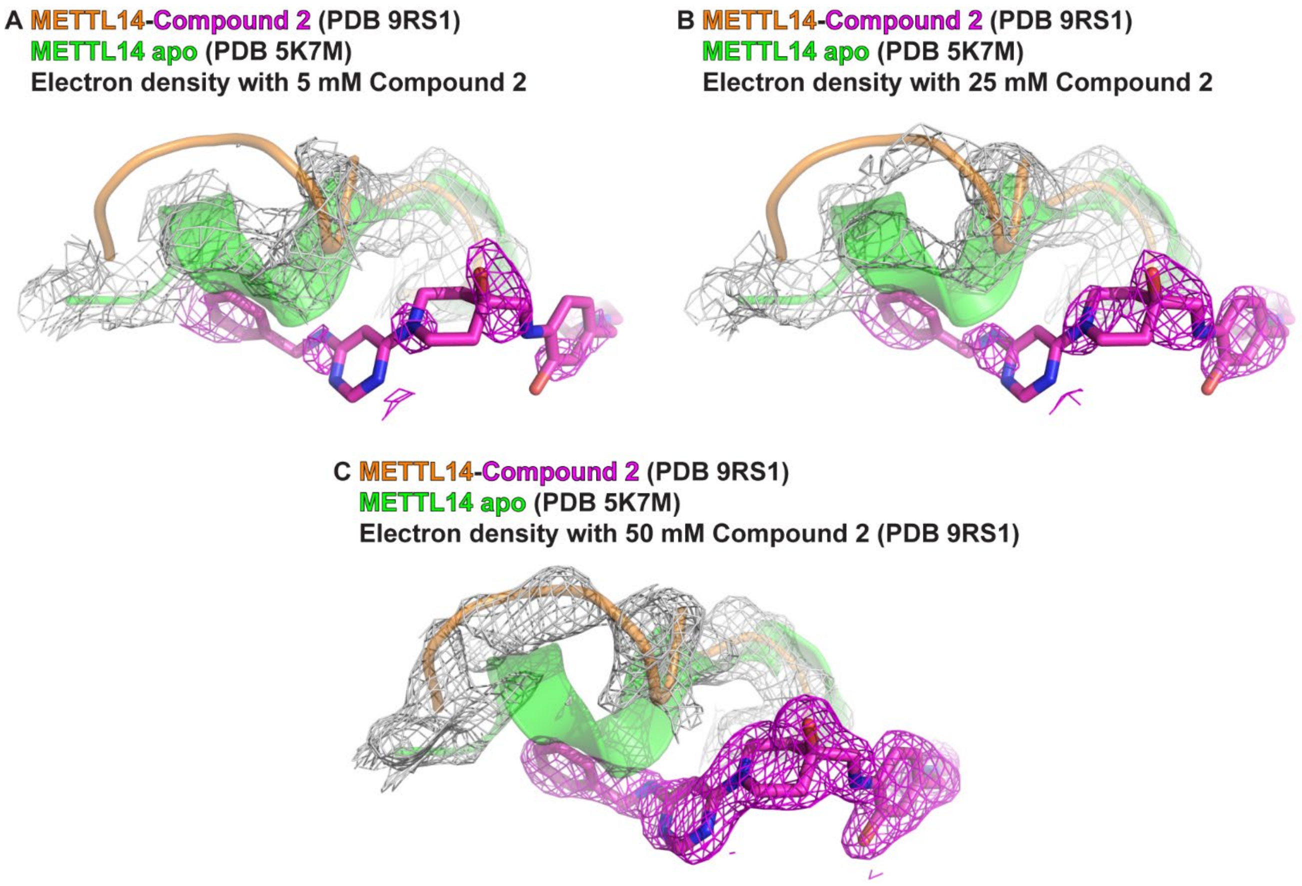

Figure S10. Analysis of electron density in the cryptic pocket of METTL14 in crystals of METTL3-14 soaked with different concentrations of compound **2**. Shown is an overlay of the METTL14-compound **2** structure with METTL14 apo. Mesh isosurfaces contoured at 1.0 sigma were generated from the electron density maps obtained from METTL3-14 crystals soaked with 5 mM (A), 25 mM (B), and 50 mM (C) of compound **2** showing partial compound **2** density and closed METT14 exosite (A), partial compound **2** density and partially open METT14 exosite (B), and complete compound **2** density and open METT14 exosite (C).

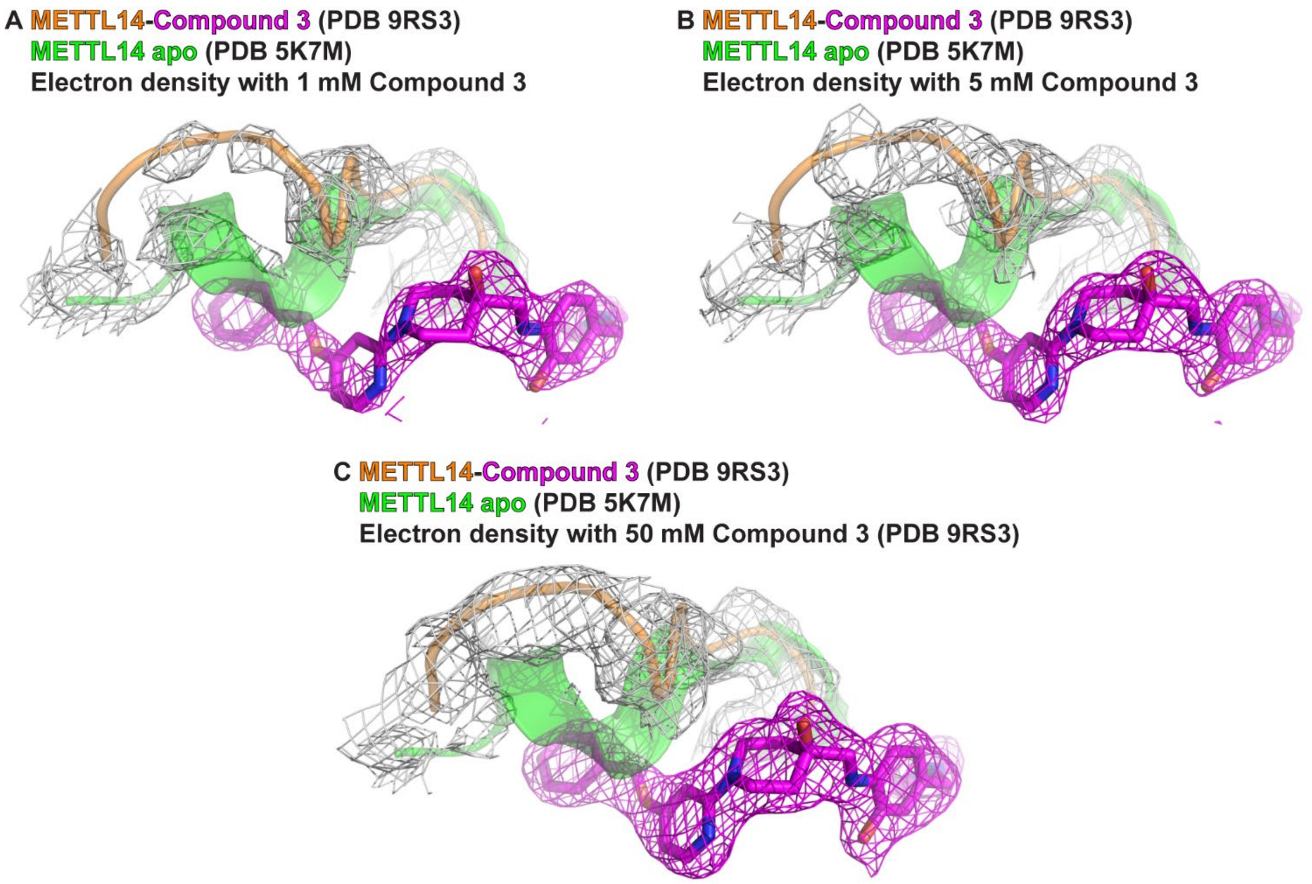

Figure S11. Same as Figure S10 for compound **3**. Mesh isosurfaces contoured at 1.0 sigma were generated from the electron density maps obtained from METTL3-14 crystals soaked with 1 mM (A), 5 mM (B), and 50 mM (C) of compound **3** showing weaker compound **3** density and partially open METT14 exosite (A,B), and complete compound **3** density and open METT14 exosite (C).

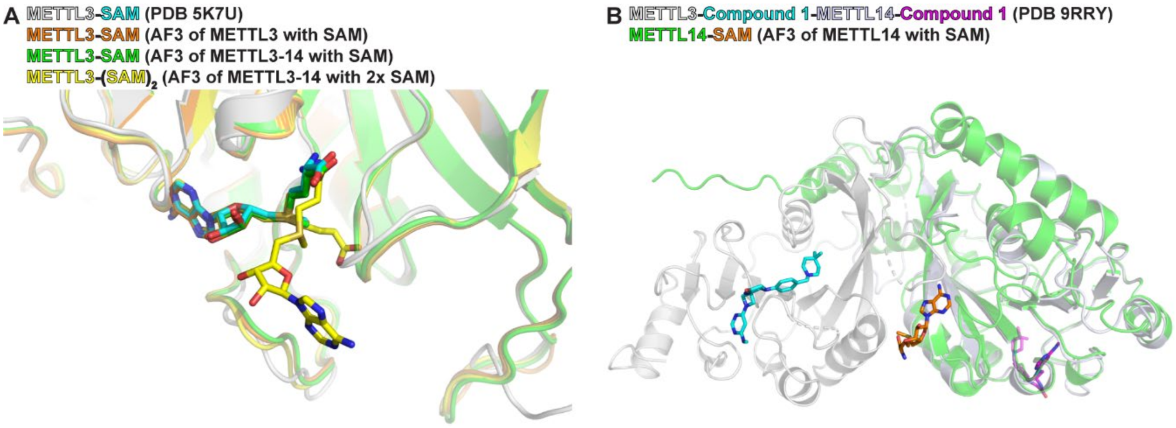

Figure S12. Alphafold3 (AF3) predictions of METTL3-14 with SAM. (A) Structural overlay of the crystal structure of holo METTL3 bound to SAM with different AF3 predictions involving METTL3 and SAM. (B) Structural overlay of the crystal structure of METTL3-14 bound to compound **1** with the AF3 prediction of METTL14 with SAM. METTL3-14 backbone is shown in cartoon representation, compounds are shown as sticks. Color coding is indicated at the top of each panel.

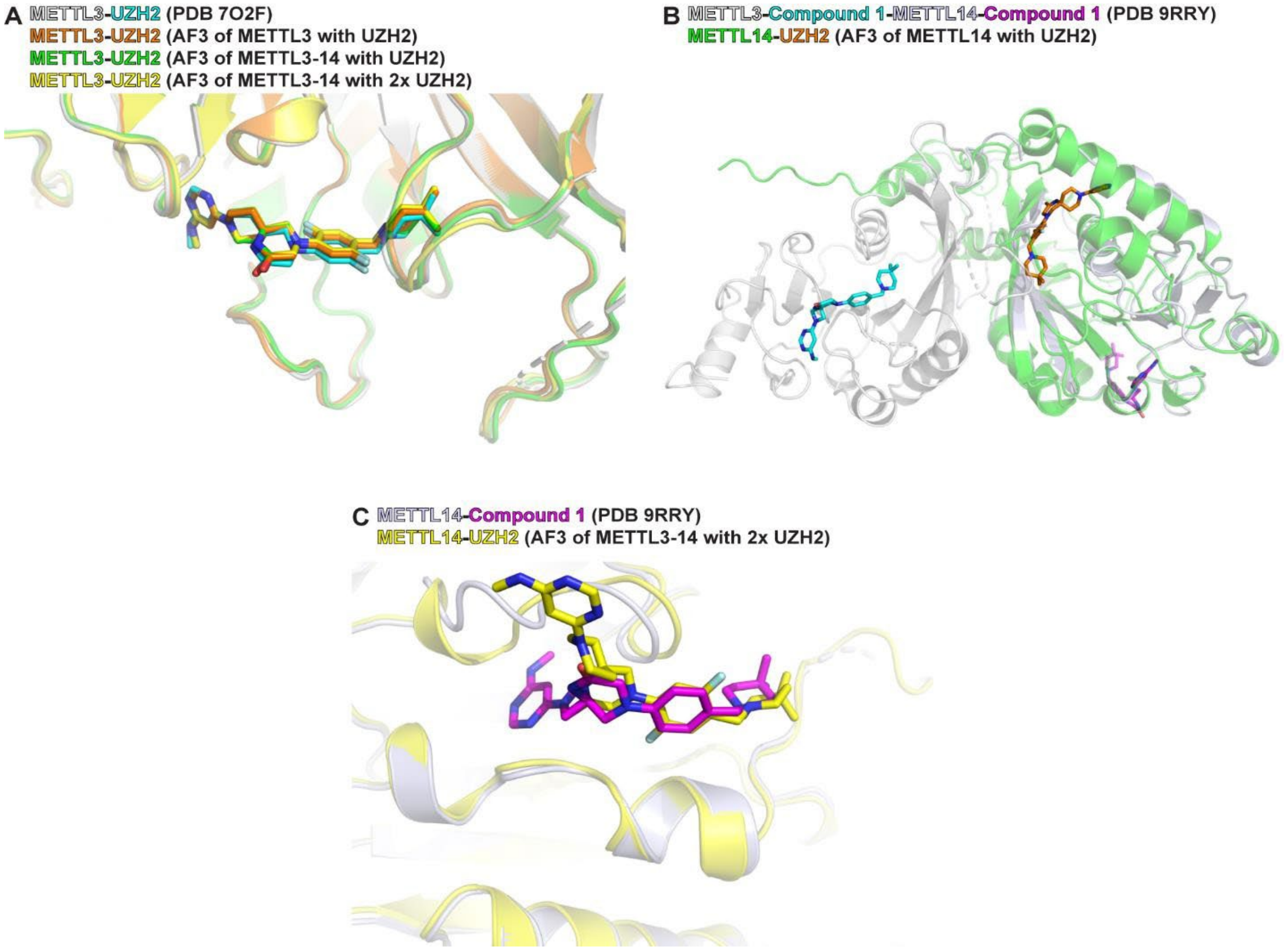

Figure S13. Alphafold3 (AF3) predictions of METTL3-14 with established METTL3 inhibitor UZH2. (A) Structural overlay of the crystal structure of METTL3 bound to UZH2 with different AF3 predictions involving METTL3 and UZH2. (B) Structural overlay of the crystal structure of METTL3- 14 bound to compound **1** with the AF3 prediction of METTL14 with UZH2. (C) Structural overlay of the crystal structure of METTL14 bound to compound **1** with METTL14 bound to UZH2 from the AF3 prediction of METTL3-14 with two copies of UZH2. METTL3-14 backbone is shown in cartoon representation, compounds are shown as sticks. Color coding is indicated at the top of each panel.

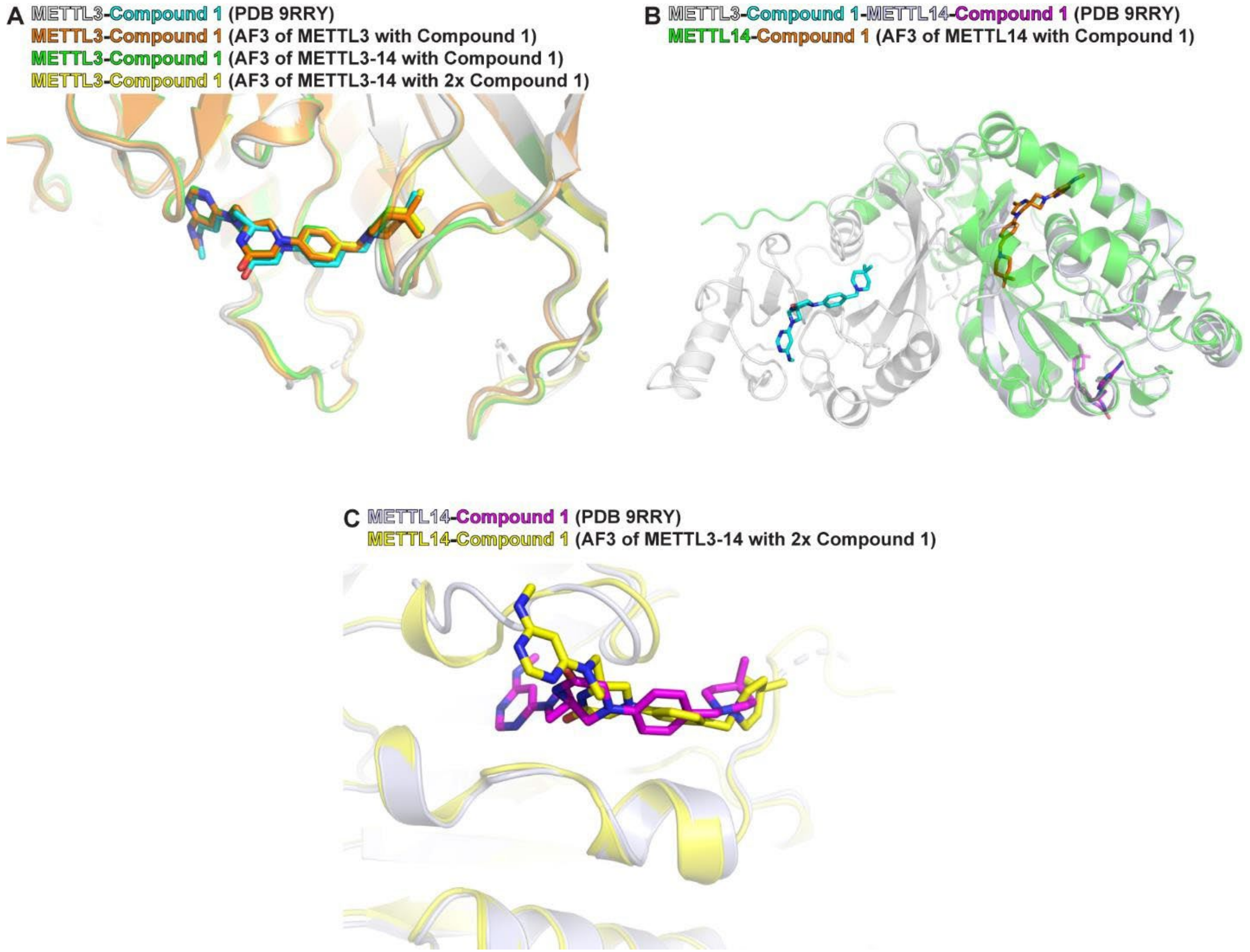

Figure S14. Alphafold3 (AF3) predictions of METTL3-14 with METTL14 exosite binder compound **1**. (A) Structural overlay of the crystal structure of METTL3 bound to compound **1** with different AF3 predictions involving METTL3 and compound **1**. (B) Structural overlay of the crystal structure of METTL3-14 bound to compound **1** with the AF3 prediction of METTL14 with compound **1**. (C) Structural overlay of the crystal structure of METTL14 bound to compound **1** with METTL14 bound to compound **1** from the AF3 prediction of METTL3-14 with two copies of compound **1**. METTL3-14 backbone is shown in cartoon representation, compounds are shown as sticks. Color coding is indicated at the top of each panel.

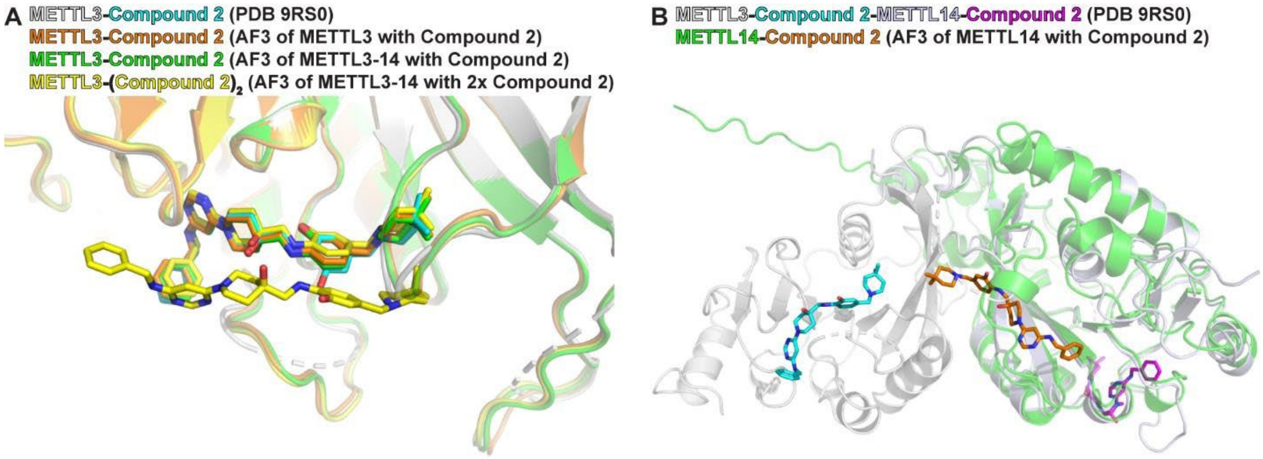

Figure S15. Alphafold3 (AF3) predictions of METTL3-14 with METTL14 exosite binder compound **2**. (A) Structural overlay of the crystal structure of METTL3 bound to compound **2** with different AF3 predictions involving METTL3 and compound **2**. (B) Structural overlay of the crystal structure of METTL3-14 bound to compound **2** with the AF3 prediction of METTL14 with compound **2**. METTL3-14 backbone is shown in cartoon representation, compounds are shown as sticks. Color coding is indicated at the top of each panel.

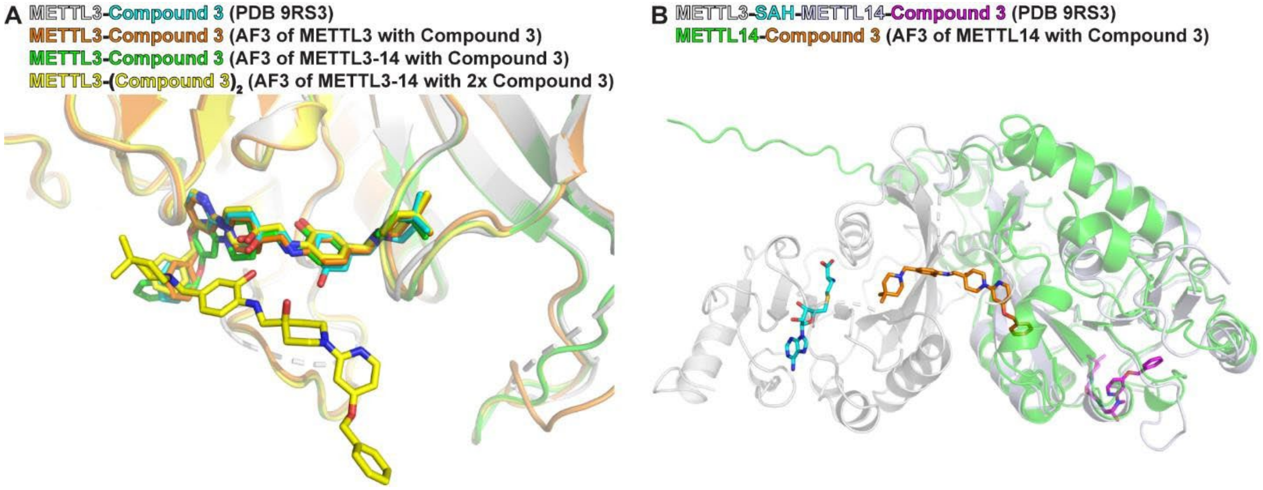

Figure S16. Alphafold3 (AF3) predictions of METTL3-14 with METTL14 exosite binder compound **3**. (A) Structural overlay of the crystal structure of METTL3 bound to compound **3** with different AF3 predictions involving METTL3 and compound **3**. (B) Structural overlay of the crystal structure of METTL3-14 bound to SAH and compound **3** in METTL3 and METTL14, respectively, with the AF3 prediction of METTL14 with compound **3**. METTL3-14 backbone is shown in cartoon representation, compounds are shown as sticks. Color coding is indicated at the top of each panel.

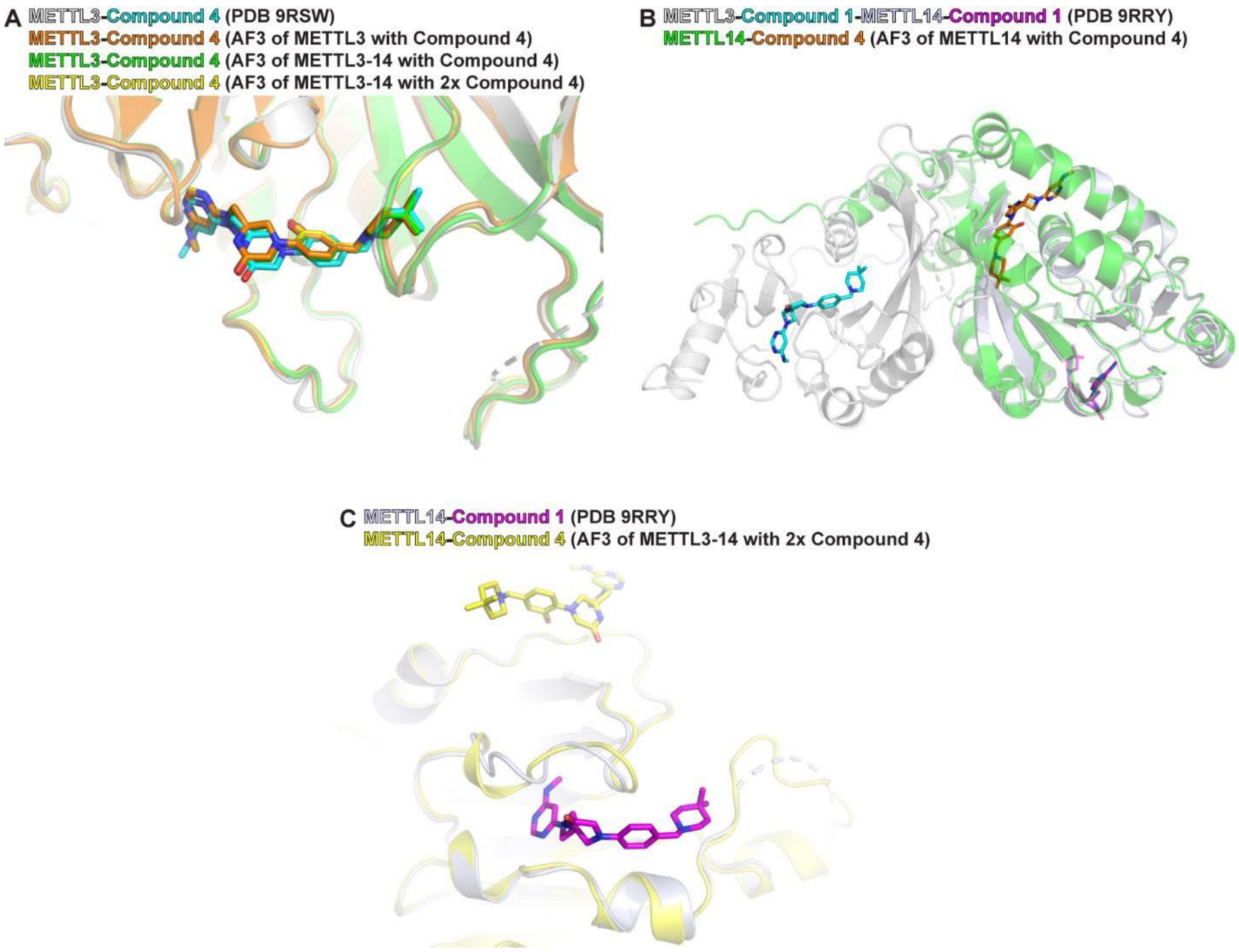

Figure S17. Alphafold3 (AF3) predictions of METTL3-14 with compound **4**. (A) Structural overlay of the crystal structure of METTL3 bound to compound **4** with different AF3 predictions involving METTL3 and compound **4**. (B) Structural overlay of the crystal structure of METTL3-14 bound to compound **1** with the AF3 prediction of METTL14 with compound **4**. (C) Structural overlay of the crystal structure of METTL14 bound to compound **1** with METTL14 bound to compound **4** from the AF3 prediction of METTL3-14 with two copies of compound **4**. METTL3-14 backbone is shown in cartoon representation, compounds are shown as sticks. Color coding is indicated at the top of each panel.

Table S1. PDB IDs of METTL3-14 complexes without exosite binders used in Supplementary Figure 1E.

PDB ID	PDB ID	PDB ID	PDB ID	PDB ID	PDB ID	PDB ID	PDB ID	PDB ID	PDB ID
5IL2 [b]	5K7U [b]	5L6D [b]	7O28 [c]	6TTX [c]	7NHJ [c]	7O08 [c]	7OED [c]	7OEK [c]	8PW9 [c]
8PW8 [c]	9G4U [c]	5L6E [b]	7O2H [c]	6TU1 [c]	7NHV [c]	7O09 [c]	7OEE [c]	7OEL [c]	8PWA [c]
5IL1 [b]	9G4S [c]	7O0M [c]	7O2X [c]	6Y4G [c]	7NI7 [c]	7O0L [c]	7OEF [c]	7OEM [c]	8PWB [c]
5K7W [b]	9G4W [c]	7O0P [c]	6TTP [c]	7ACD [c]	7NI8 [c]	7O29 [c]	7OEG [c]	7OQL [c]	
5TEY [b]	7RX6 [c]	7O0Q [c]	6TTT [c]	7NHG [c]	7NI9 [c]	7O2E [c]	7OEH [c]	7OQO [c]	
5K7M [a]	7RX7 [c]	7O0R [c]	6TTV [c]	7NHH [c]	7NIA [c]	7O2F [c]	7OEI [c]	7OQP [c]	
5IL0 [a]	7RX8 [c]	7O27 [c]	6TTW [c]	7NHI [c]	7NID [c]	7O2I [c]	7OEJ [c]	8BN8 [c]	

[a] Apo METTL3-14.
[b] Holo METTL3-14 with SAM or SAH bound in the METTL3 SAM-pocket.
[c] METTL3-14 with inhibitor bound in the METTL3 SAM-pocket.

Table S2. Summary of the compounds that were designed based on compound **2**. The inhibitory activity was measured by the reader-based HTRF assay (see Materials and Methods). The compounds were soaked into crystals of the METTL3-14 complex that had SAH bound in the METTL3 active site. Modifications of compounds with respect to their parent compound **2** are emphasized (red circles). SAH = SAH bound to METTL3; Empty = empty METTL14 exosite; NA = not available.

Compound number	2D structure	MW [Da]	IC_{50} [μM] (Hill slope)	Bound to METTL3	Bound to METTL14	PDB ID (Resol. [Å])
**5**	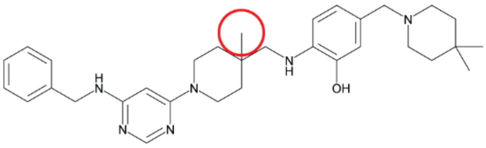	528.8	0.3 (1.6)	SAH	Empty	NA
**6**	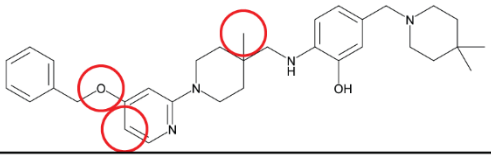	528.7	40.1 (1.5)	SAH	Empty	NA
**7**	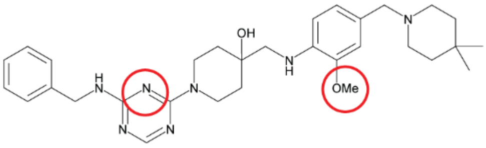	545.7	1.8 (1.7)	SAH	Empty	NA
**8**	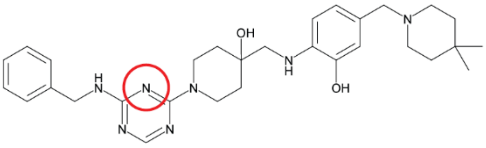	531.7	0.8 (1.1)	SAH	Empty	NA

Table S3. Summary of the compounds, IC_{50} values, and METTL3-14 crystal titration with different compound concentrations. The crystal structures were obtained by soaking the compounds into crystals of the METTL3-14 complex that had SAH bound in the METTL3 active site. Modifications of compound **3** compared to parent compound **2** are emphasized (red circles). Yes = Compound is bound; SAH = SAH bound to METTL3; Empty = empty METTL14 exosite; PED = partial electron density; WED = weaker electron density; PO = partially open; ND = not determined; NA = not available.

Compound number	2D structure	MW [Da]	IC_{50} [µM] (Hill slope)	Soaking concentration [mM]	Bound to METTL3	Bound to METTL14	METTL14 exosite	PDB ID (Resol. [Å])
1	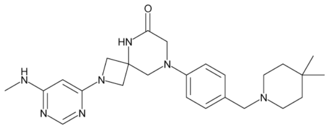	449.6	0.47 (1.7)	200	Yes	Yes	Open	9RRY (1.9)
				25	Yes	Empty	Closed	NA
				5	Yes	Empty	Closed	NA
				1	Yes	Empty	Closed	NA
2	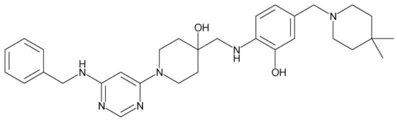	530.7	0.49 (1.3)	200	Yes	Yes	Open	9RS0 (2.0)
				50	SAH	Yes	Open	9RS1 (1.7)
				25	Yes	Yes, PED	PO	NA
				5	Yes	Yes, PED	Closed	NA
				1	Yes	Empty	Closed	NA
3	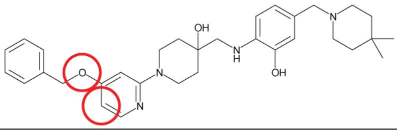	530.7	>500 (ND)	50	SAH	Yes	Open	9RS3 (2.1)
				25	SAH	Yes	Open	NA
				5	SAH	Yes	PO	NA
				1	SAH	Yes, WED	PO	NA

Table S4. Interface predicted template modelling (ipTM) scores for Alphafold3 (AF3) predictions of METTL3-14 with different small-molecule binders. The ipTM values are given for each small-molecule binder in each AF3 prediction. For the prediction with the METTL3-14 complex and one copy (1x) of the small-molecule binder, the ipTM is given for METTL3 (left) and METTL14 (right). For the prediction with the METTL3-14 complex and two copies (2x) of the small-molecule binder, the ipTM is given for copy 1 (top) and copy 2 (bottom) of the compound for METTL3 (left) and METTL14 (right).

Compound / Protein	UZH2	SAM	Compound 1	Compound 2	Compound 3	Compound 4
M3	0.96	0.98	0.96	0.94	0.81	0.97
M14	0.72	0.70	0.86	0.68	0.67	0.93
M3_M14 with 1x Compound	0.96, 0.92	0.96, 0.91	0.95, 0.91	0.94, 0.90	0.84, 0.82	0.96, 0.92
M3_M14 with 2x Compound	0.45, 0.46<br>0.54, 0.54	0.46, 0.49<br>0.69, 0.62	0.44, 0.45<br>0.58, 0.57	0.54, 0.52<br>0.35, 0.40	0.31, 0.34<br>0.46, 0.46	0.36, 0.44<br>0.66, 0.67

Table S5. Crystallography Data Collection and Refinement Statistics. Values for high-resolution shell in parenthesis.

	Compound **1** complex (PDB 9RRY)	Compound **2** complex (PDB 9RS0)	Compound **2** complex with SAH in METTL3 (PDB 9RS1)	Compound **3** complex with SAH in METTL3 (PDB 9RS3)	Compound **4** complex with empty METTL14 exosite (PDB 9RSW)
Parameter	Value	Value	Value	Value	Value
Wavelength (Å)	1	1	1	1	1
Resolution range (Å)	44.65 – 1.914 (1.96 – 1.91)	49.6 – 2.0 (2.05 – 2.0)	44.56 – 1.726 (1.76 – 1.73)	44.68 – 2.106 (2.17 – 2.11)	44.82 – 2.56 (2.72 – 2.56)
Space group	P 32 2 1	P 32 2 1	P 32 2 1	P 32 2 1	P 32 2 1
Unit cell (Å, °)	63.98 63.98 226.18 90 90 120	63.79 63.79 225.22 90 90 120	63.86 63.86 225.75 90 90 120	63.94 63.94 226.85 90 90 120	64.19 64.19 227.34 90 90 120
Total reflections	348839 (23531)	363121 (26753)	450115 (12716)	263274 (22377)	143736 (23461)
Unique reflections	79807 (5369)	69232 (5324)	107823 (4982)	59909 (5354)	33126 (5500)
Multiplicity	4.4 (4.4)	5.2 (5.0)	4.2 (2.6)	4.4 (4.2)	4.3 (4.3)
Completeness (%)	99.88 (99.64)	99.93 (99.86)	99.76 (96.33)	99.70 (98.15)	98.98 (98.11)
Mean I/σ(I)	8.88 (0.62)	13.74 (1.46)	11.97 (0.65)	10.16 (0.58)	6.81 (0.75)
Wilson B-factor	46.86	36.07	34.42	51.31	71.73
R-merge	0.08013 (2.675)	0.06732 (0.9749)	0.04932 (1.263)	0.06911 (2.062)	0.1202 (1.934)
R-meas	0.0911 (3.049)	0.07489 (1.091)	0.05622 (1.57)	0.07858 (2.356)	0.1364 (2.186)
R-pim	0.04289 (1.451)	0.03248 (0.4841)	0.02667 (0.9123)	0.03705 (1.127)	0.06326 (1.001)
CC1/2	0.998 (0.238)	0.999 (0.527)	0.999 (0.26)	0.999 (0.166)	0.995 (0.35)
CC*	1 (0.621)	1 (0.831)	1 (0.643)	1 (0.533)	0.999 (0.72)
Reflections used in refinement	42521 (2787)	36991 (2797)	57192 (2679)	32124 (2814)	18129 (2913)
Reflections used for R-free	2128 (139)	1849 (140)	2862 (134)	1605 (141)	907 (146)
R-work	0.2211 (0.4766)	0.1930 (0.2567)	0.1999 (0.3271)	0.2010 (0.3299)	0.2183 (0.3103)
R-free	0.2508 (0.5375)	0.2408 (0.2846)	0.2199 (0.3931)	0.2357 (0.3830)	0.2506 (0.3966)
No. of non-hydrogen atoms	3497	3749	3743	3589	3402
– macromolecules	3320	3417	3468	3454	3355
– ligands	70	82	43	46	39
– solvent	107	250	232	89	8
Protein residues	435	442	444	446	441
RMS(bonds) (Å)	0.008	0.007	0.009	0.010	0.009
RMS(angles) (°)	0.91	0.87	0.97	1.05	0.98

Ramachandran favored (%)	95.94	96.26	96.97	95.15	95.78
Ramachandran allowed (%)	3.82	3.27	2.80	3.70	3.75
Ramachandran outliers (%)	0.24	0.47	0.23	1.15	0.47
Rotamer outliers (%)	1.21	0.29	0.00	0.29	3.34
Clashscore	3.55	2.70	2.37	3.28	8.17
Average B-factor	51.69	39.59	40.56	57.67	78.43
– macromolecules	51.55	39.07	40.19	57.81	78.40
– ligands	60.54	47.44	44.50	52.39	82.07
– solvent	50.44	44.24	45.37	54.92	71.56

Chemistry

Compound 2

Compound **2**, chemical synthesis scheme:

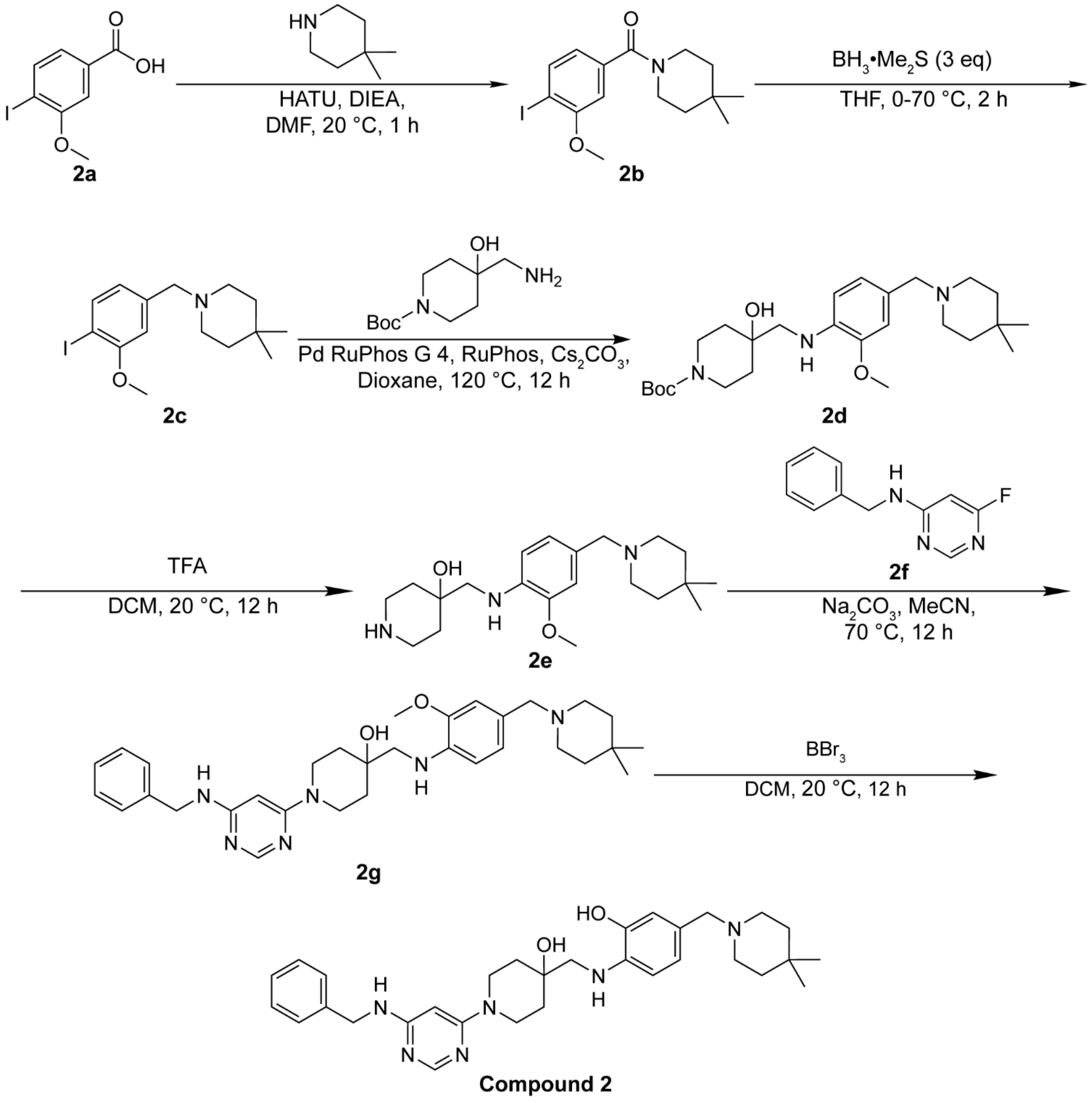

Compound **2**, chemical synthesis scheme of intermediate 2f:

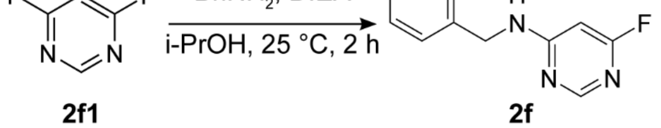

Compound **2**, NMR spectrum:

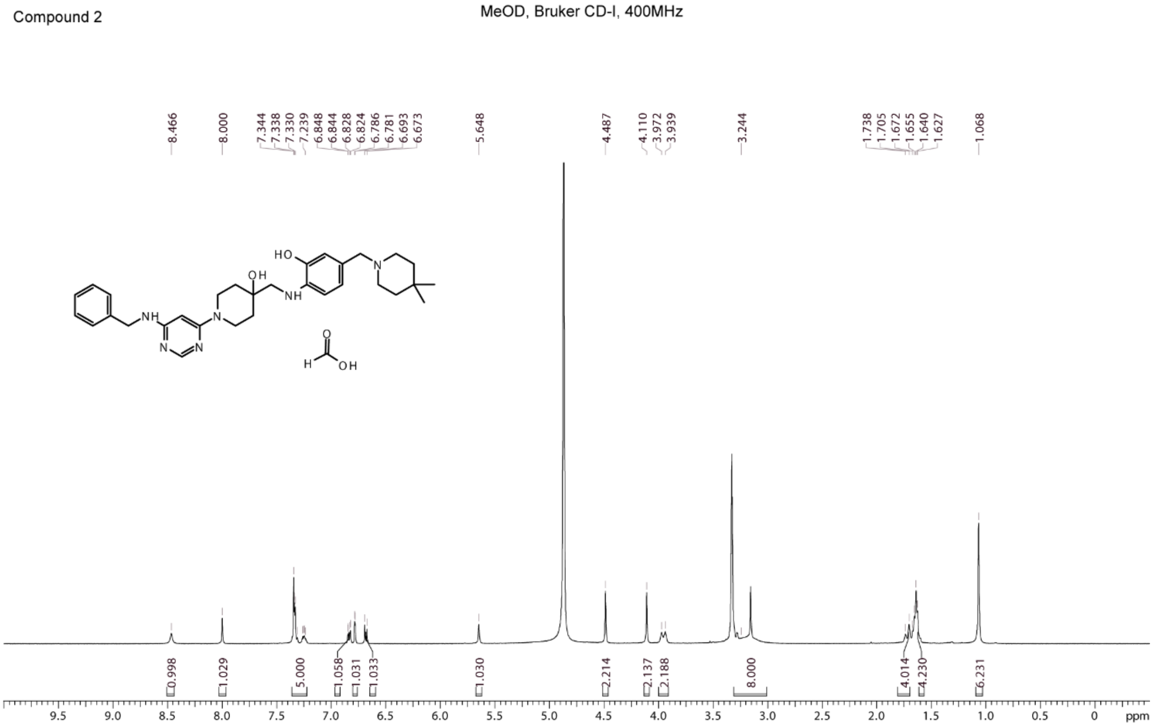

1H NMR (400 MHz, METHANOL-d4) δ = 8.47 (br s, 1H), 8.00 (s, 1H), 7.36 - 7.22 (m, 5H), 6.84 (dd, J = 1.6, 8.0 Hz, 1H), 6.78 (d, J = 1.8 Hz, 1H), 6.68 (d, J = 8.0 Hz, 1H), 5.65 (s, 1H), 4.49 (s, 2H), 4.11 (s, 2H), 3.96 (br d, J = 13.4 Hz, 2H), 3.24 (br s, 8H), 1.77 - 1.66 (m, 4H), 1.65 - 1.60 (m, 4H), 1.07 (s, 6H).

Compound **2**, LC-MS analysis:

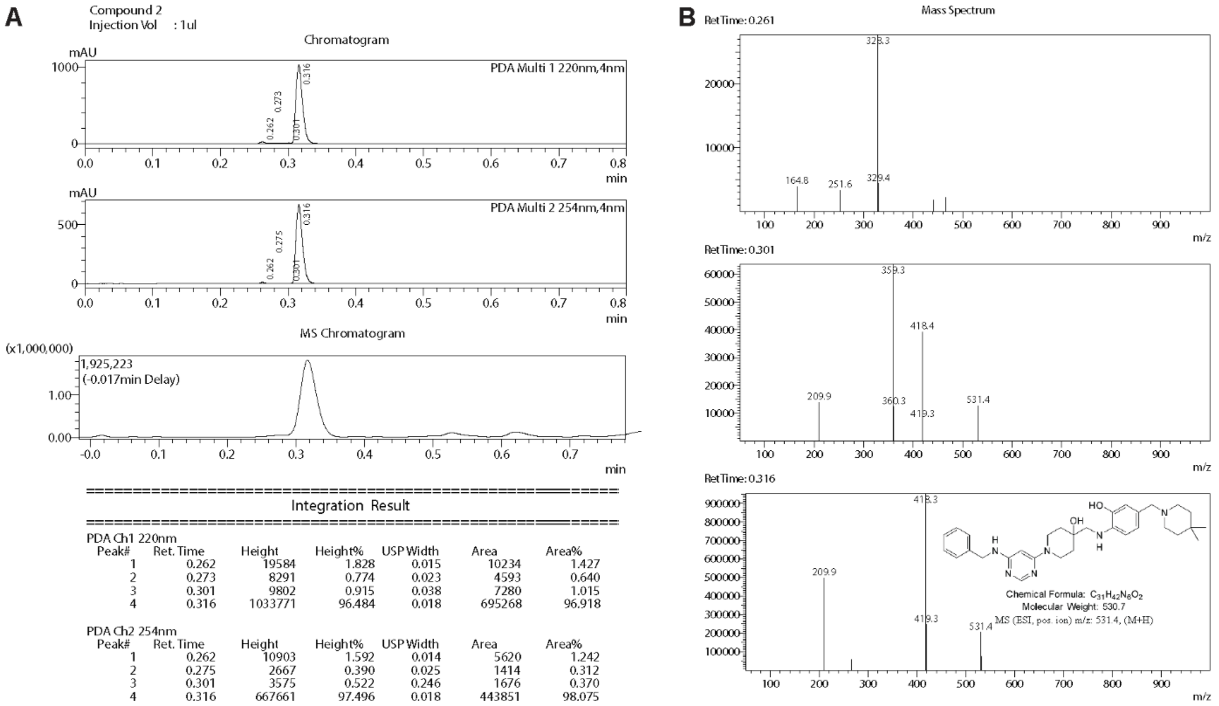

Integration Result

PDA Ch1 220nm

Peak#	Ret. Time	Height	Height%	USP Width	Area	Area%
1	0.262	19584	1.828	0.015	10234	1.427
2	0.273	8291	0.774	0.023	4593	0.640
3	0.301	9802	0.915	0.038	7280	1.015
4	0.316	1033771	96.484	0.018	695268	96.918

PDA Ch2 254nm

Peak#	Ret. Time	Height	Height%	USP Width	Area	Area%
1	0.262	10903	1.592	0.014	5620	1.242
2	0.275	2667	0.390	0.025	1414	0.312
3	0.301	3575	0.522	0.246	1676	0.370
4	0.316	667661	97.496	0.018	443851	98.075

Compound 3

Compound **3**, chemical synthesis scheme:

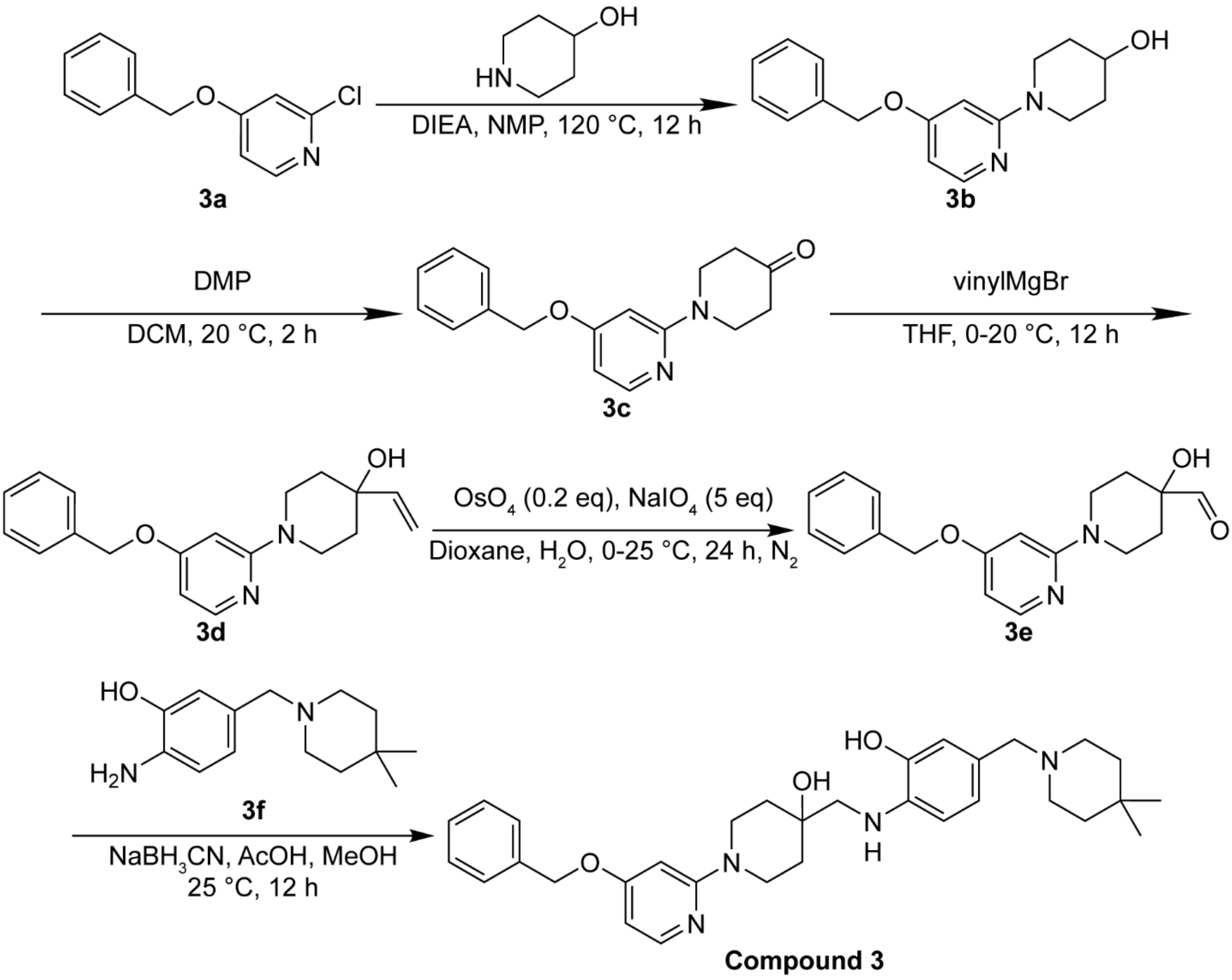

Compound **3**, chemical synthesis scheme of intermediate 3f:

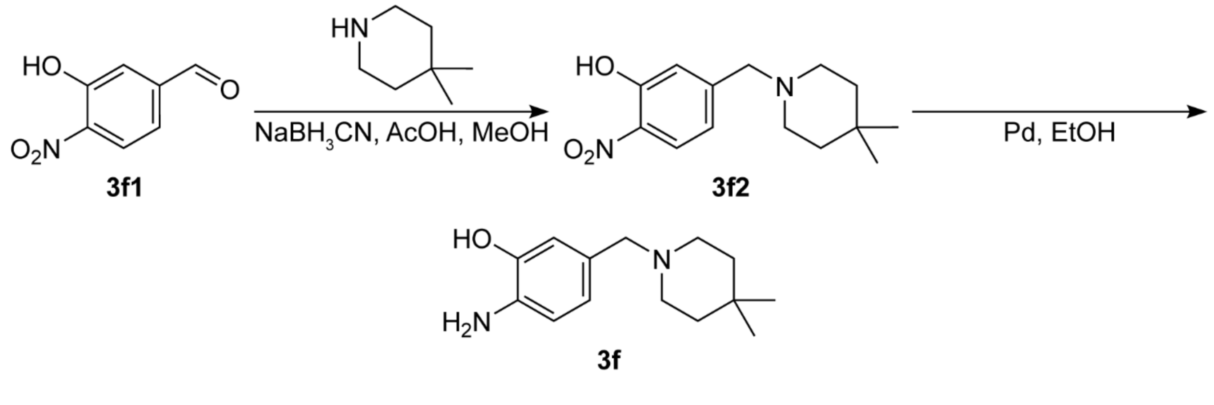

Compound **3**, NMR spectrum:

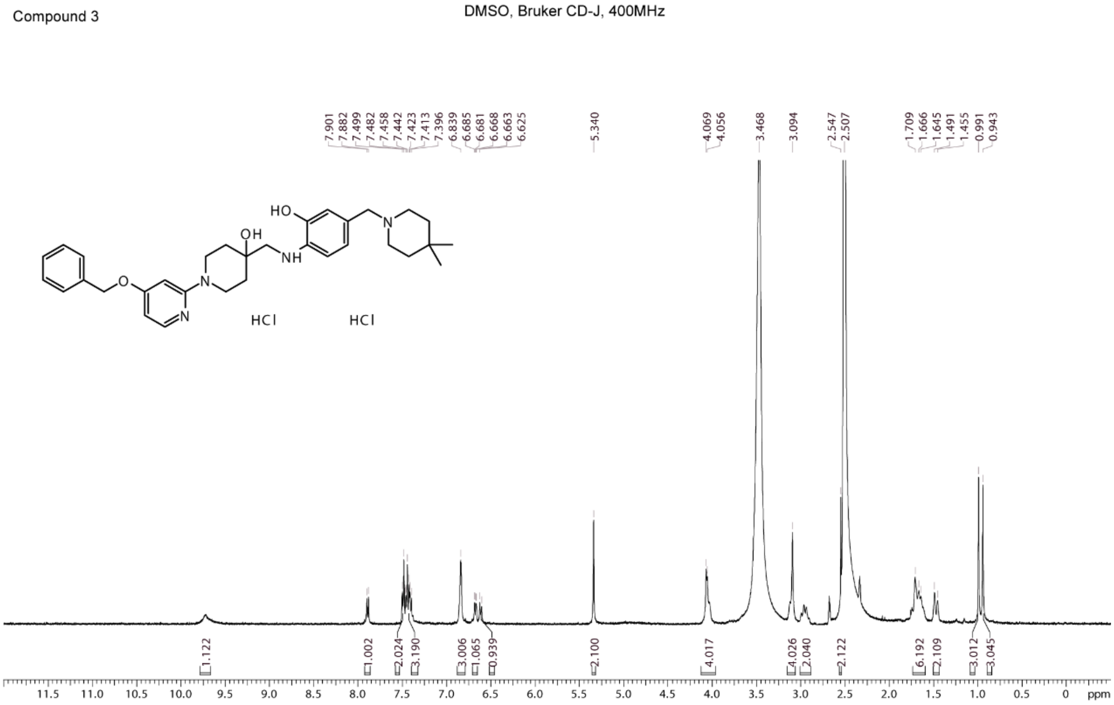

1H NMR (400 MHz, DMSO-*d6*) δ ppm 9.67 - 9.79 (m, 1 H) 7.89 (d, *J*=7.25 Hz, 1 H) 7.47 - 7.52 (m, 2 H) 7.39 - 7.46 (m, 3 H) 6.84 (br s, 3 H) 6.67 (dd, *J*=7.07, 1.69 Hz, 1 H) 6.61 (br d, *J*=8.38 Hz, 1 H) 5.34 (s, 2 H) 4.06 (br d, *J*=5.38 Hz, 4 H) 3.09 (s, 4 H) 2.89 - 3.01 (m, 2 H) 2.55 (s, 2 H) 1.59 - 1.74 (m, 6 H) 1.47 (br d, *J*=14.26 Hz, 2 H) 0.99 (s, 3 H) 0.94 (s, 3 H).

Compound **3**, LC-MS analysis:

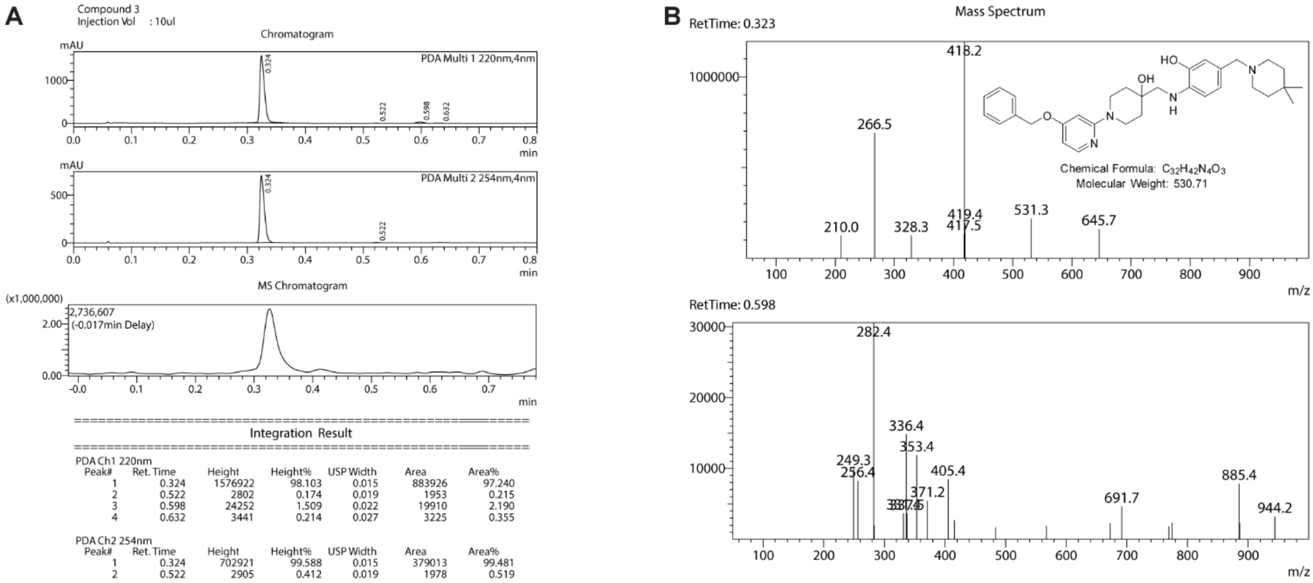

Integration Result

PDA Ch1 220nm

Peak#	Ret. Time	Height	Height%	USP Width	Area	Area%
1	0.324	1576922	98.103	0.015	883926	97.240
2	0.522	2802	0.174	0.019	1953	0.215
3	0.598	24252	1.509	0.022	19910	2.190
4	0.632	3441	0.214	0.027	3225	0.355

PDA Ch2 254nm

Peak#	Ret. Time	Height	Height%	USP Width	Area	Area%
1	0.324	702921	99.588	0.015	379013	99.481
2	0.522	2905	0.412	0.019	1978	0.519

Compound 4

Compound **4**, chemical synthesis scheme:

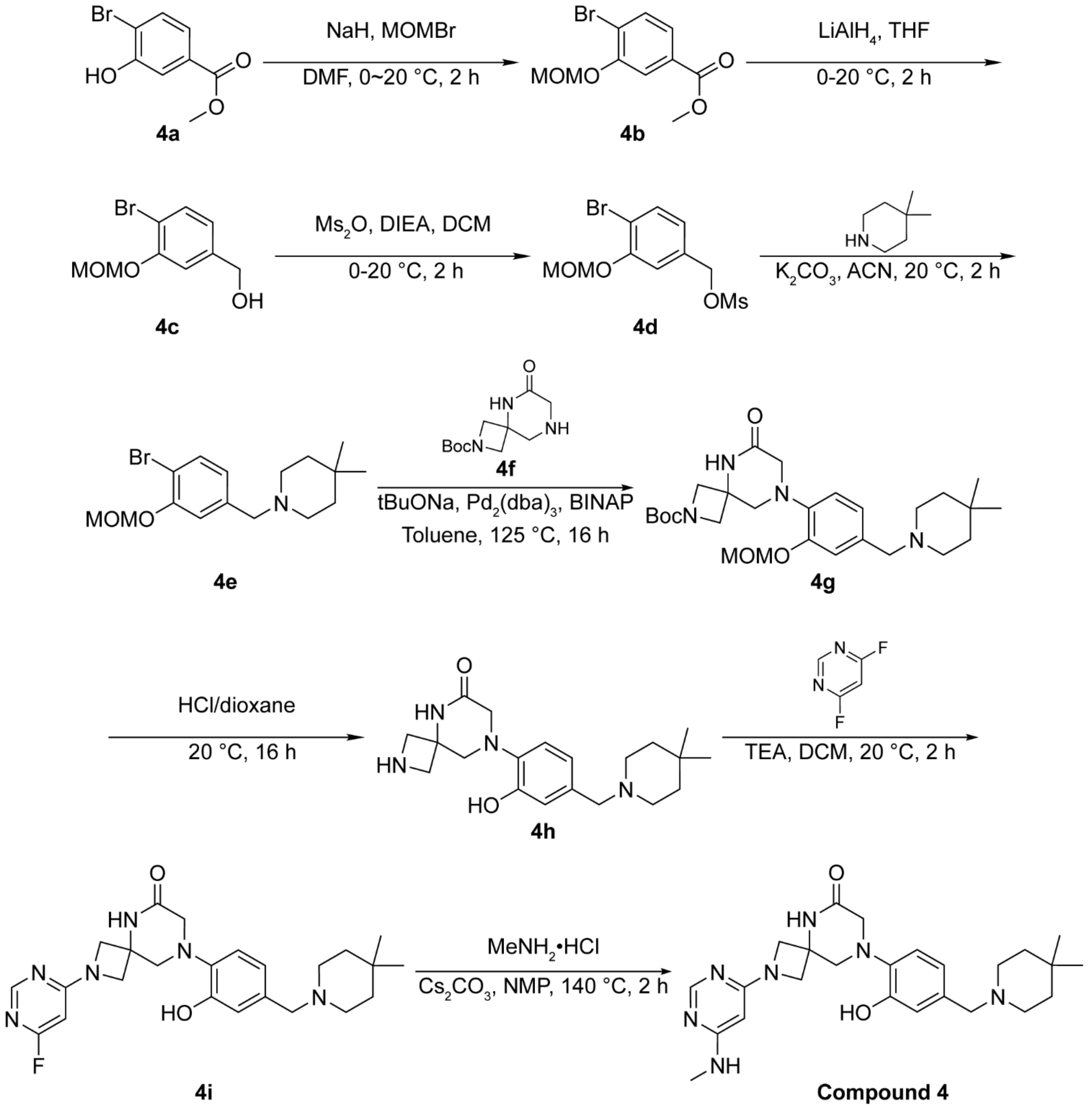

Compound **4**, chemical synthesis scheme of intermediate 4f:

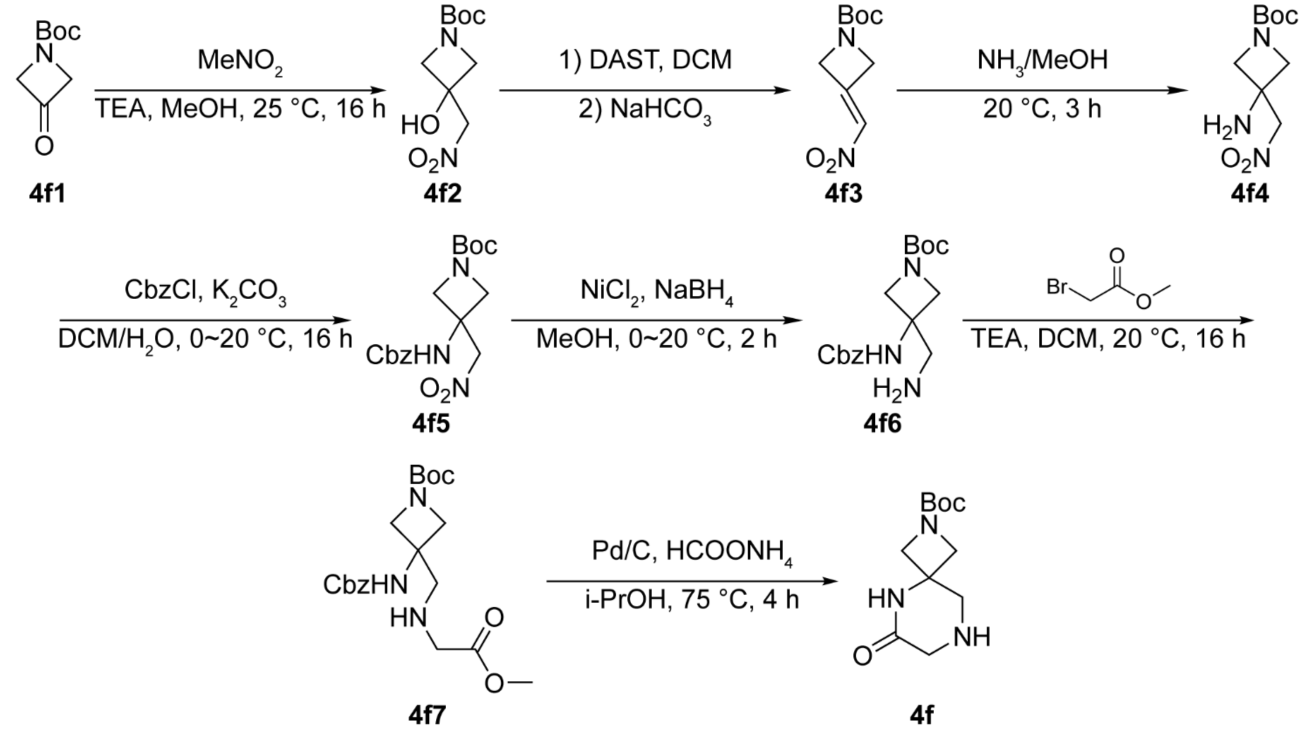

Compound **4**, NMR spectrum:

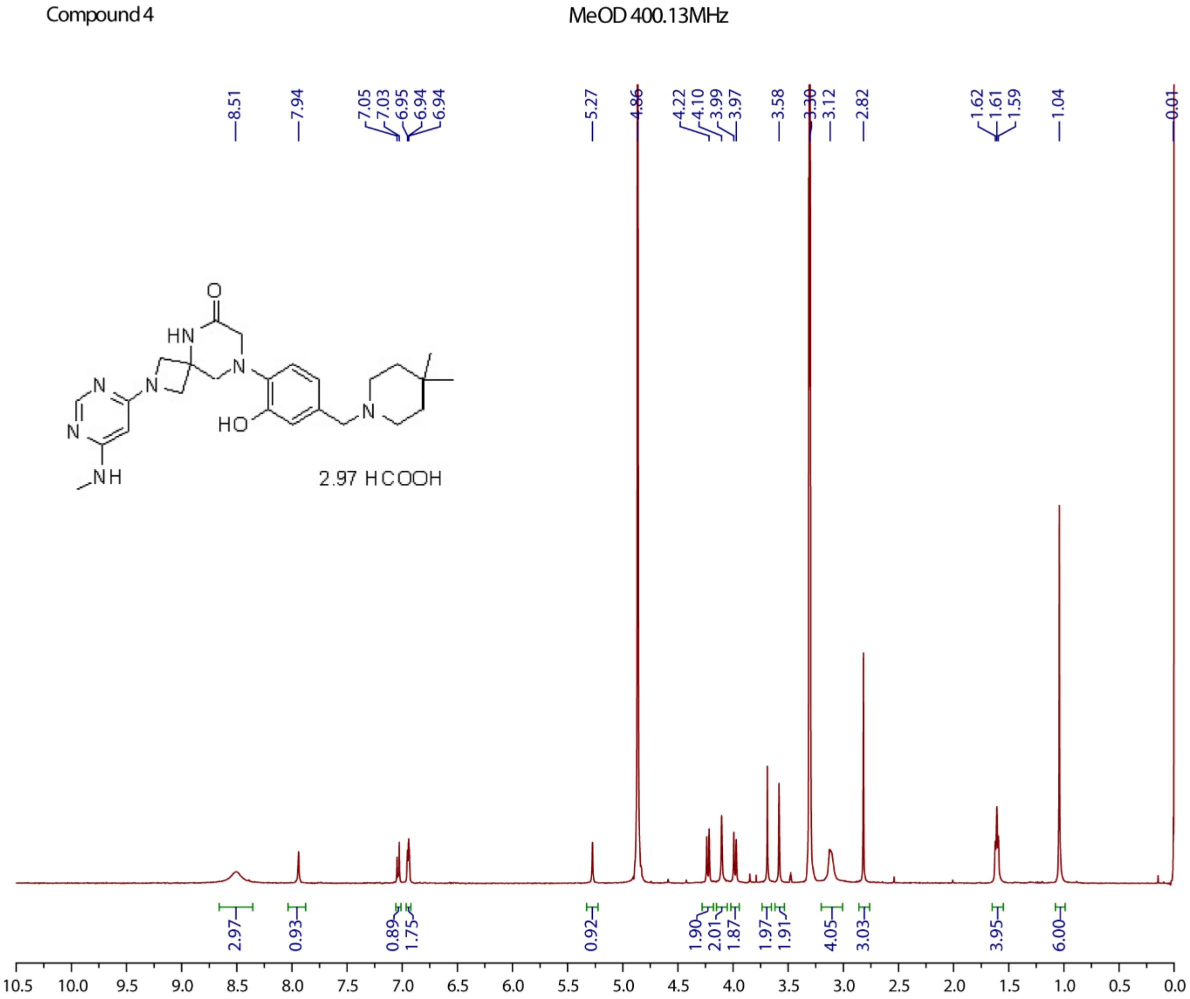

1H NMR (400 MHz, MeOD) δ7.94 (s, 1H), 7.04 (d, J = 8.6 Hz, 1H), 6.96 – 6.92 (m, 2H), 5.27 (s, 1H), 4.23 (d, J = 9.1 Hz, 2H), 4.10 (s, 2H), 3.98 (d, J = 9.0 Hz, 2H), 3.69 (s, 2H), 3.58 (s, 2H), 3.20 – 3.01 (m, 4H), 2.82 (s, 3H), 1.61 (t, J = 5.7 Hz, 4H), 1.04 (s, 6H).

Compound **4**, LC-MS analysis:

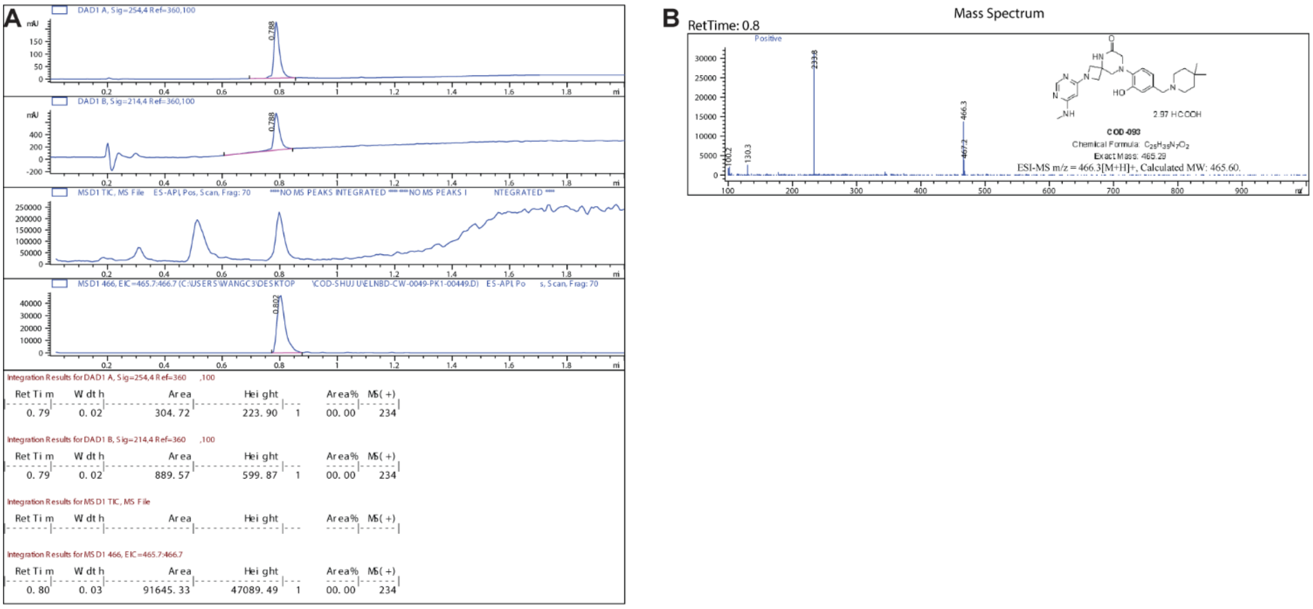

Compound 5

Compound **5**, chemical synthesis scheme:

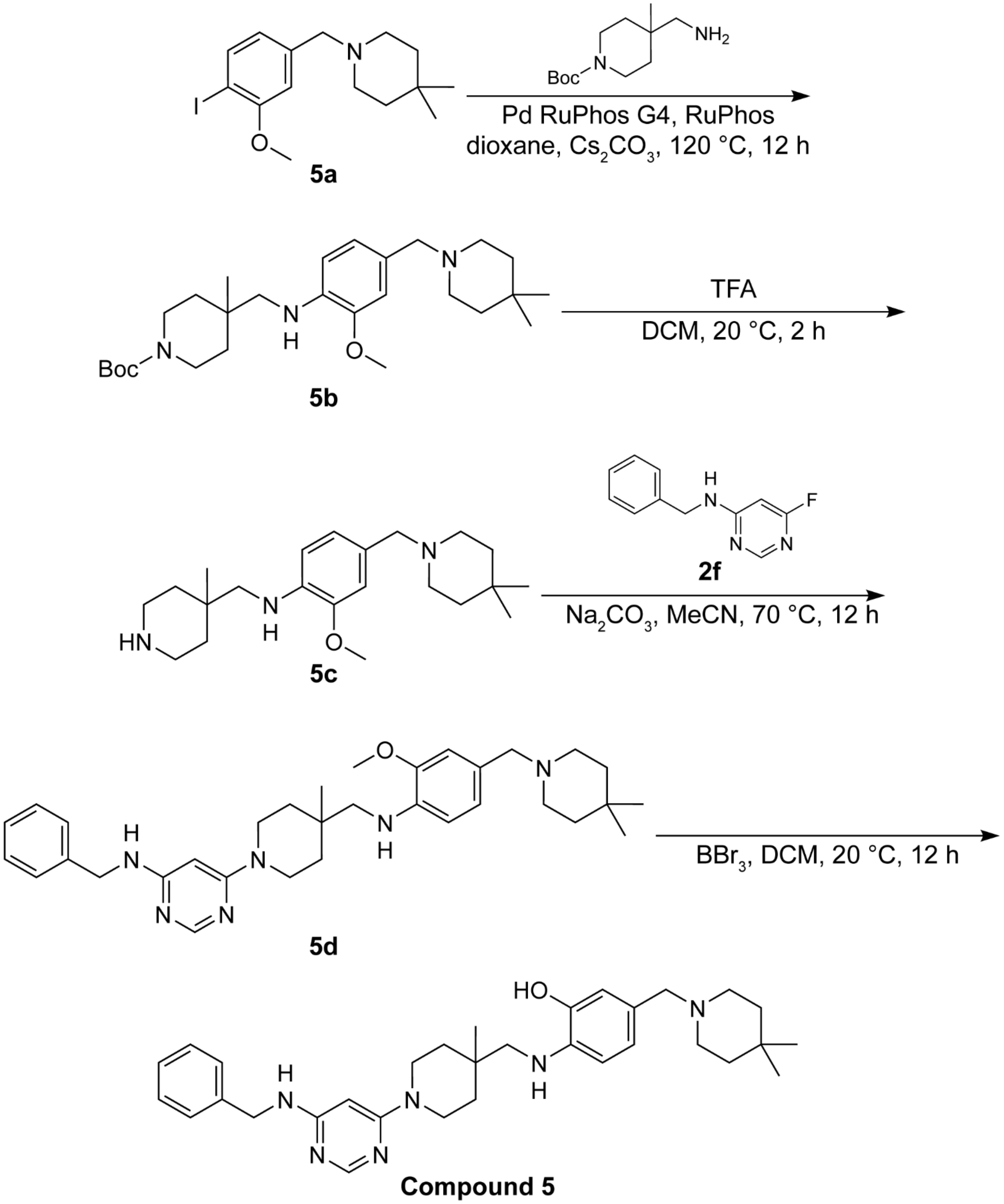

Compound **5**, NMR spectrum:

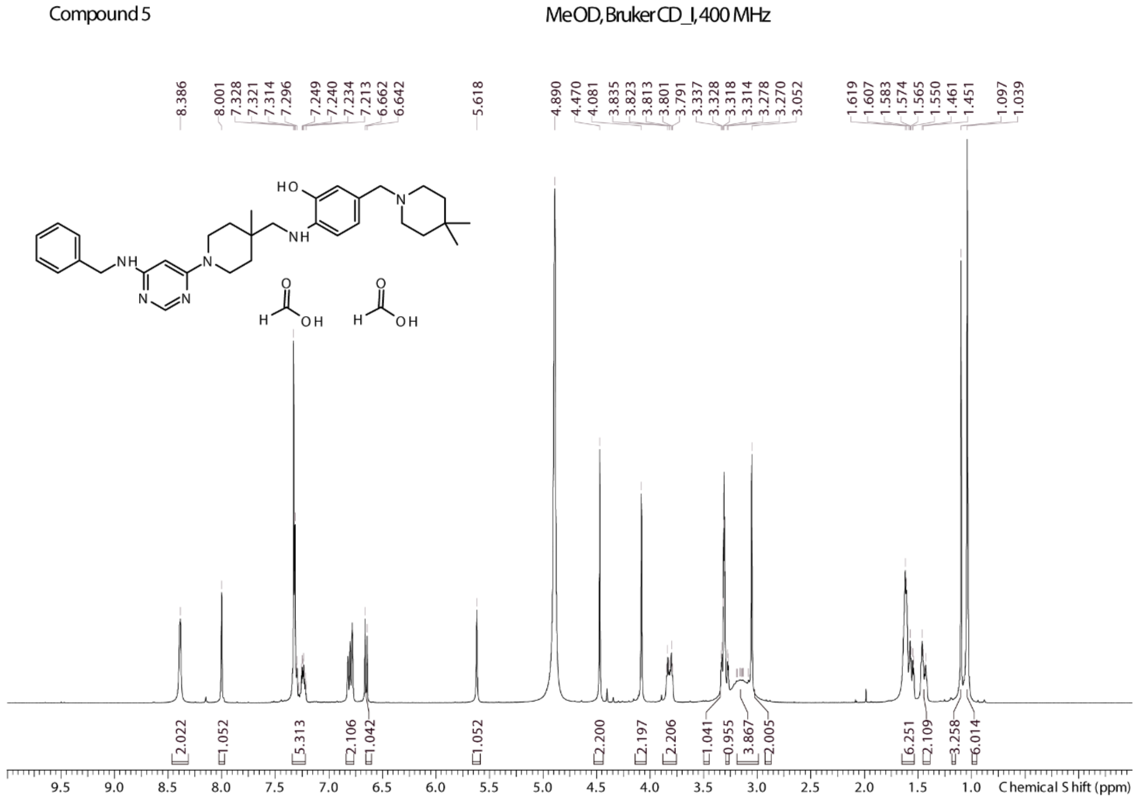

1H NMR (400 MHz, METHANOL-d4) δ = 8.39 (br s, 2H), 8.00 (s, 1H), 7.34 - 7.22 (m, 5H), 6.84 - 6.76 (m, 2H), 6.65 (d, *J* = 8.0 Hz, 1H), 5.62 (s, 1H), 4.47 (s, 2H), 4.08 (s, 2H), 3.82 (td, *J* = 4.2, 13.3 Hz, 2H), 3.33 (br d, *J* = 3.2 Hz, 1H), 3.27 (br d, *J* = 3.2 Hz, 1H), 3.26 - 3.06 (m, 4H), 3.05 (s, 2H), 1.65 - 1.53 (m, 6H), 1.48 - 1.41 (m, 2H), 1.10 (s, 3H), 1.04 (s, 6H).

Compound **5**, LC-MS analysis:

A

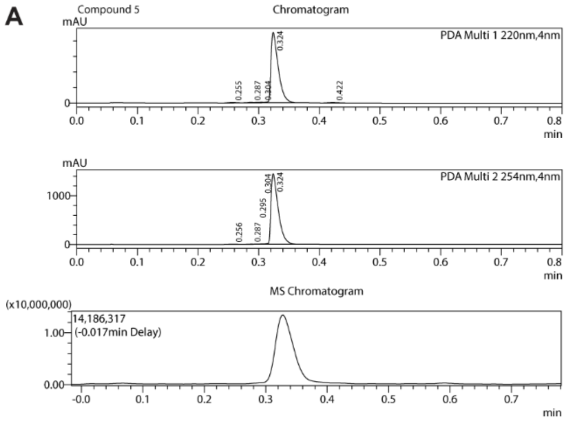

Integration Result

PDA Ch1 220nm

Peak#	Ret. Time	Height	Height%	USP Width	Area	Area%
1	0.255	13315	0.570	0.024	12981	0.671
2	0.287	15127	0.648	0.028	10138	0.524
3	0.304	17738	0.760	0.068	15434	0.798
4	0.324	2272247	97.355	0.022	1878199	97.148
5	0.422	15555	0.666	0.027	16584	0.858

PDA Ch2 254nm

Peak#	Ret. Time	Height	Height%	USP Width	Area	Area%
1	0.256	3738	0.254	0.023	3363	0.279
2	0.287	5598	0.380	0.026	3026	0.251
3	0.295	6069	0.413	0.000	2987	0.248
4	0.304	7800	0.530	0.234	3624	0.301
5	0.324	1448127	98.423	0.021	1191109	98.920

B

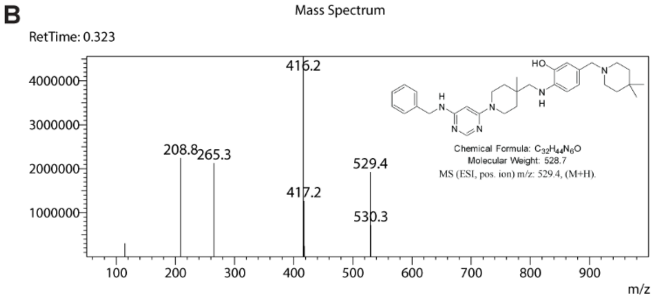

Compound 6

Compound **6**, chemical synthesis scheme:

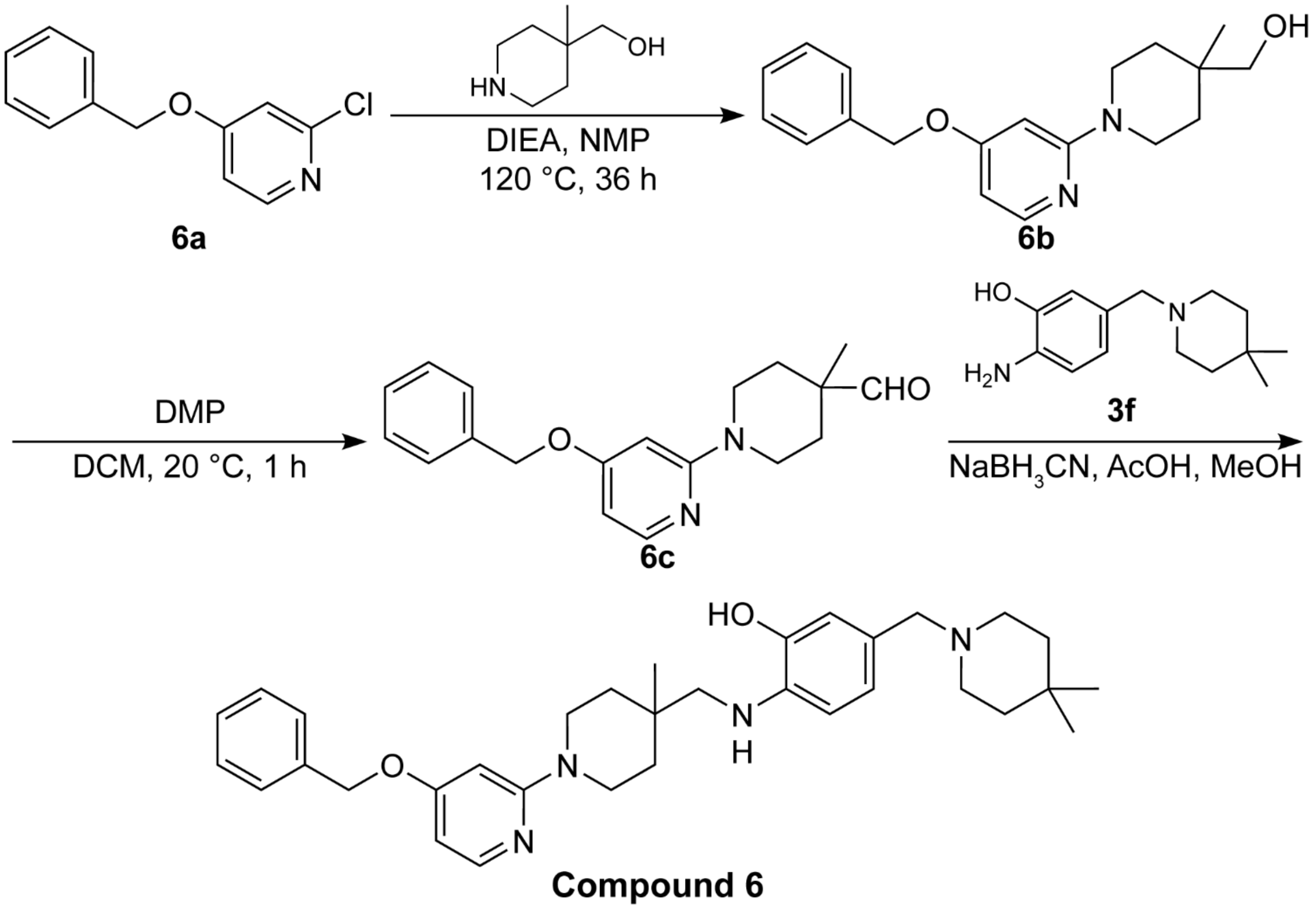

Compound **6**, NMR spectrum:

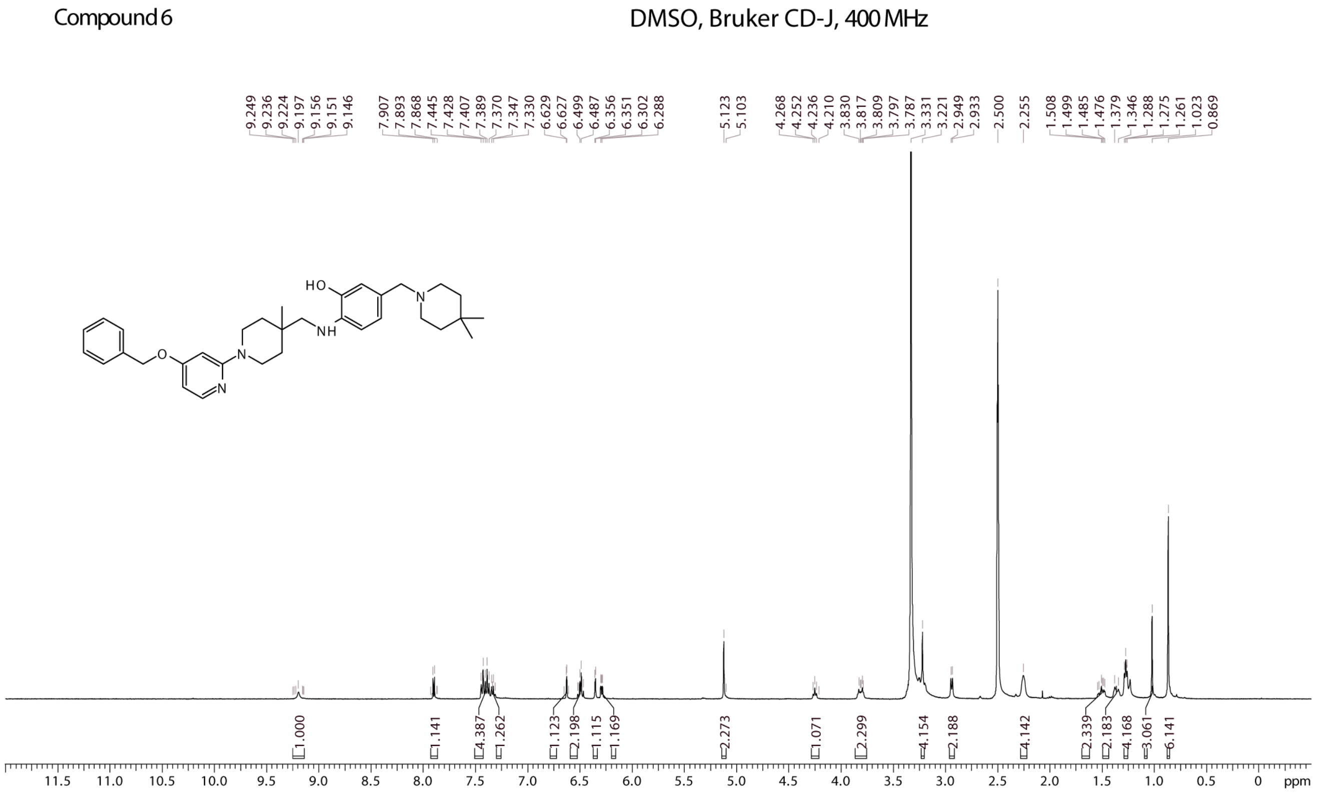

Compound **6**, LC-MS analysis:

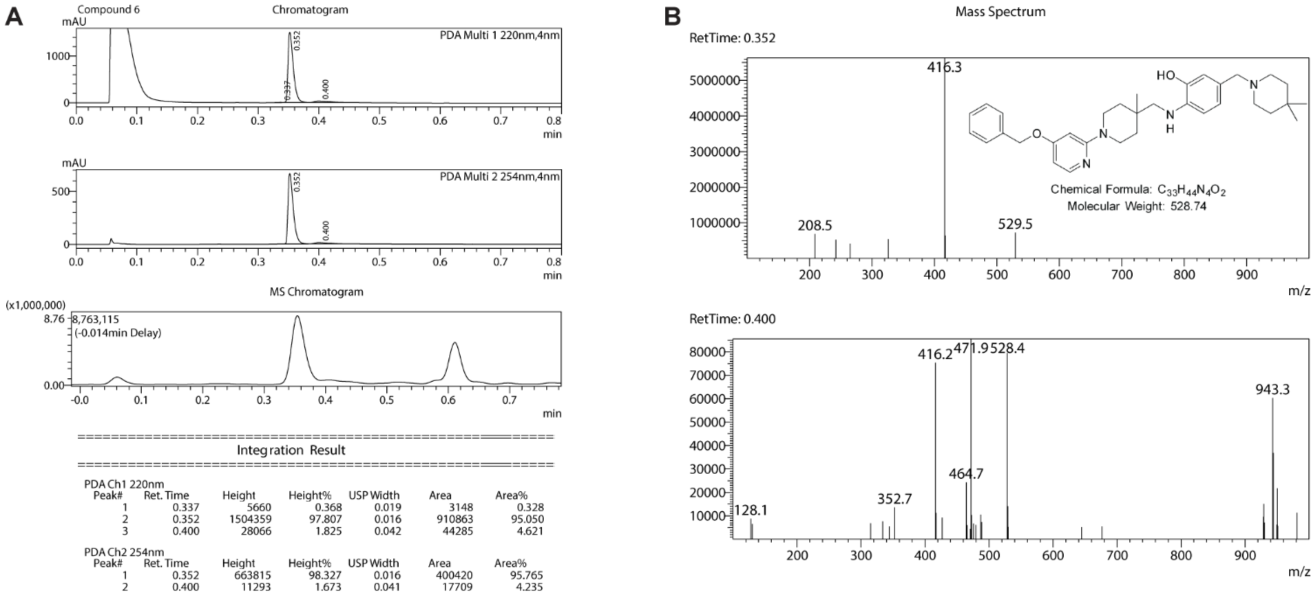

Compounds 7 and 8

Compounds **7** and **8**, chemical synthesis scheme:

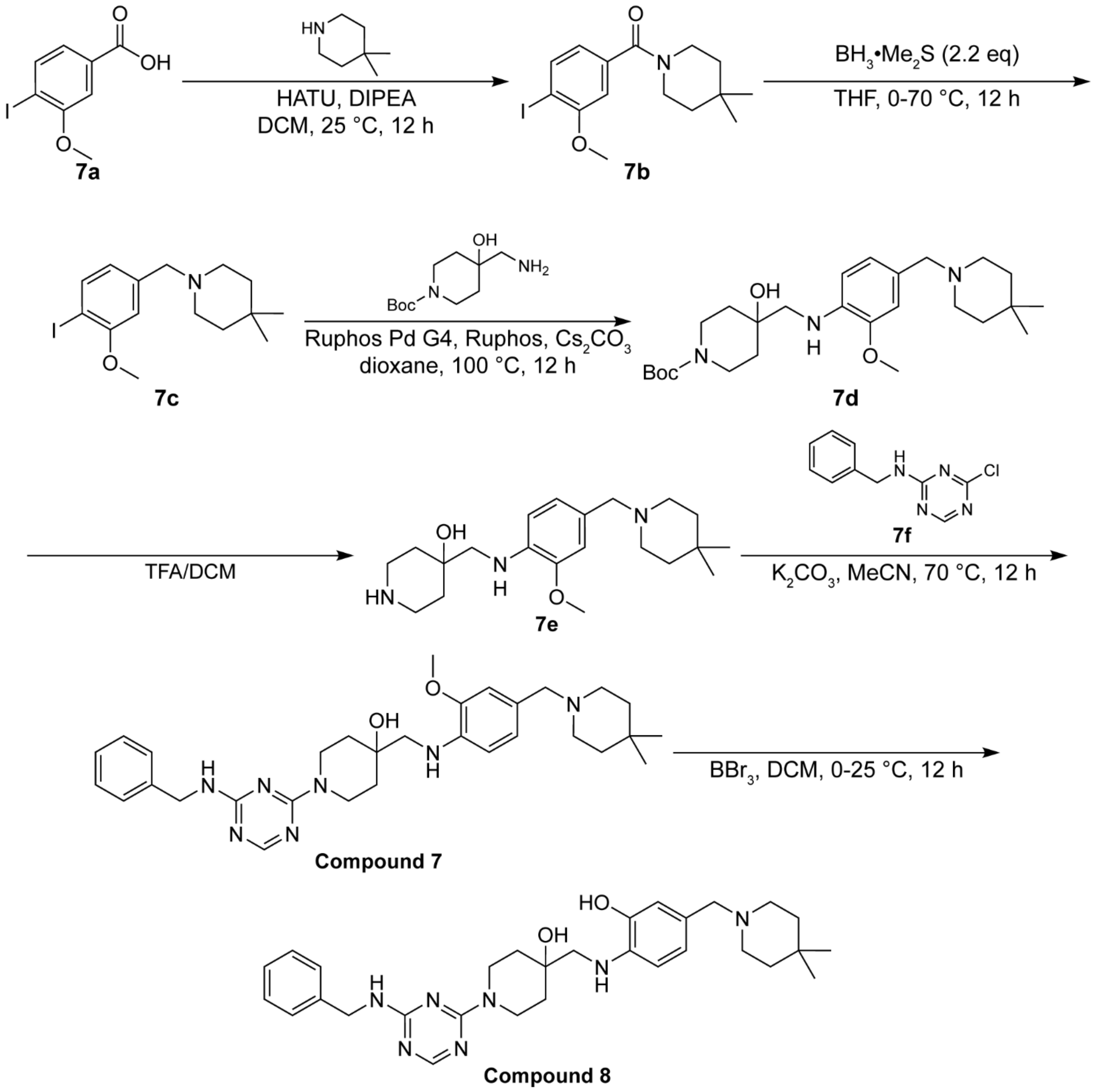

Compounds **7** and **8**, chemical synthesis scheme of intermediate 7f:

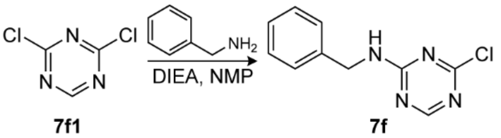

Compound **7**, NMR spectrum:

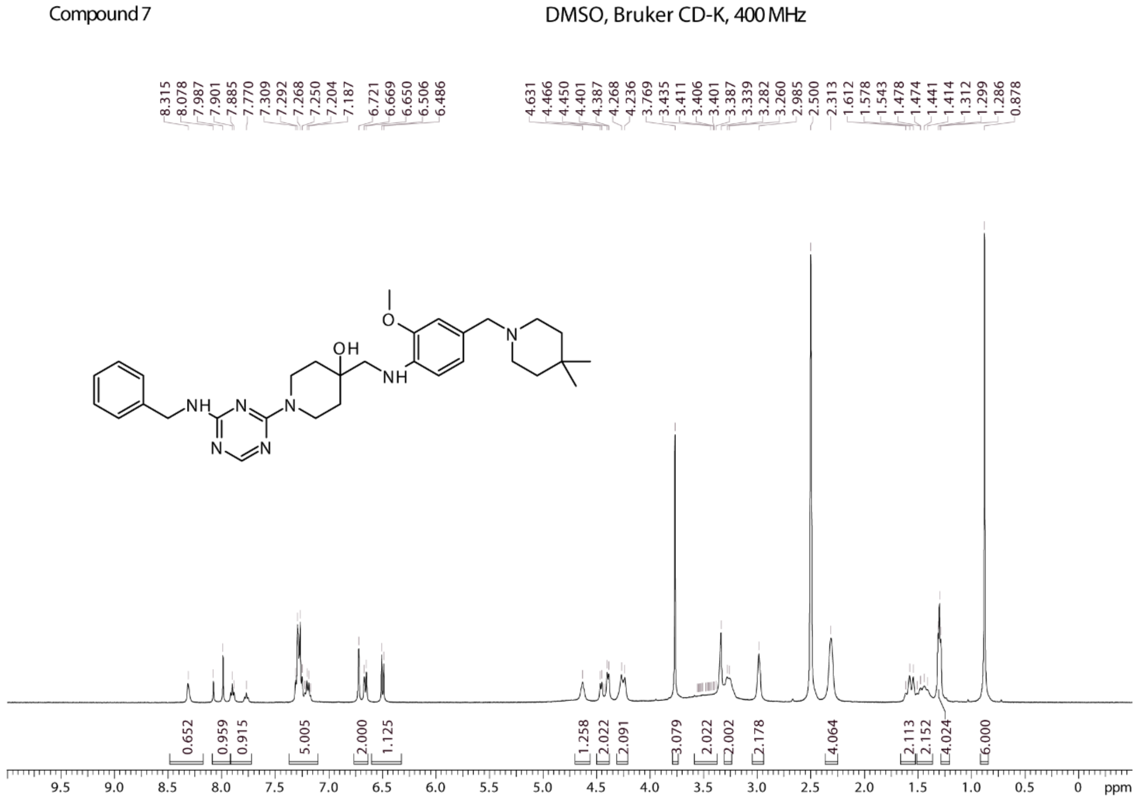

1H NMR (400 MHz, DMSO-d6) δ = 8.31 (br s, 1H), 8.09 - 7.92 (m, 1H), 7.92 - 7.72 (m, 1H), 7.37 - 7.10 (m, 5H), 6.76 - 6.63 (m, 2H), 6.50 (d, *J* = 8.0 Hz, 1H), 4.63 (br s, 1H), 4.50 - 4.38 (m, 2H), 4.25 (br d, *J* = 12.8 Hz, 2H), 3.77 (s, 3H), 3.59 - 3.38 (m, 2H), 3.27 (br d, *J* = 8.8 Hz, 2H), 2.99 (br s, 2H), 2.31 (br s, 4H), 1.66 - 1.52 (m, 2H), 1.51 - 1.36 (m, 2H), 1.30 (br t, *J* = 5.2 Hz, 4H), 0.88 (s, 6H).

Compound **7**, LC-MS analysis:

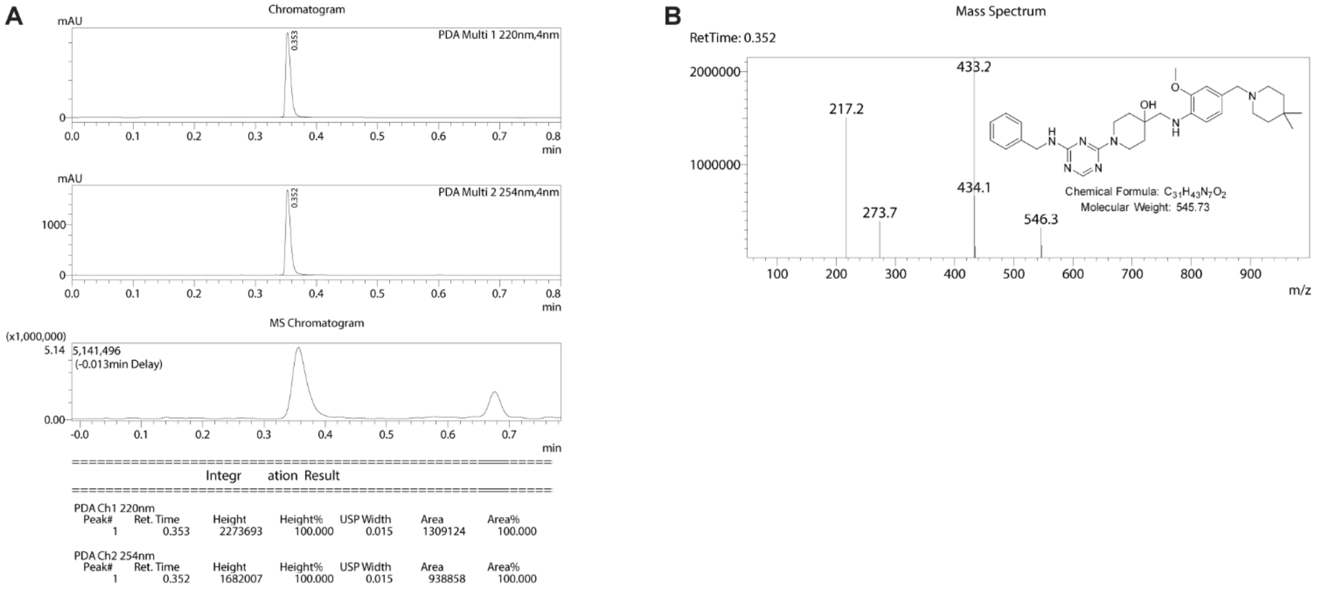

Compound **8**, NMR spectrum:

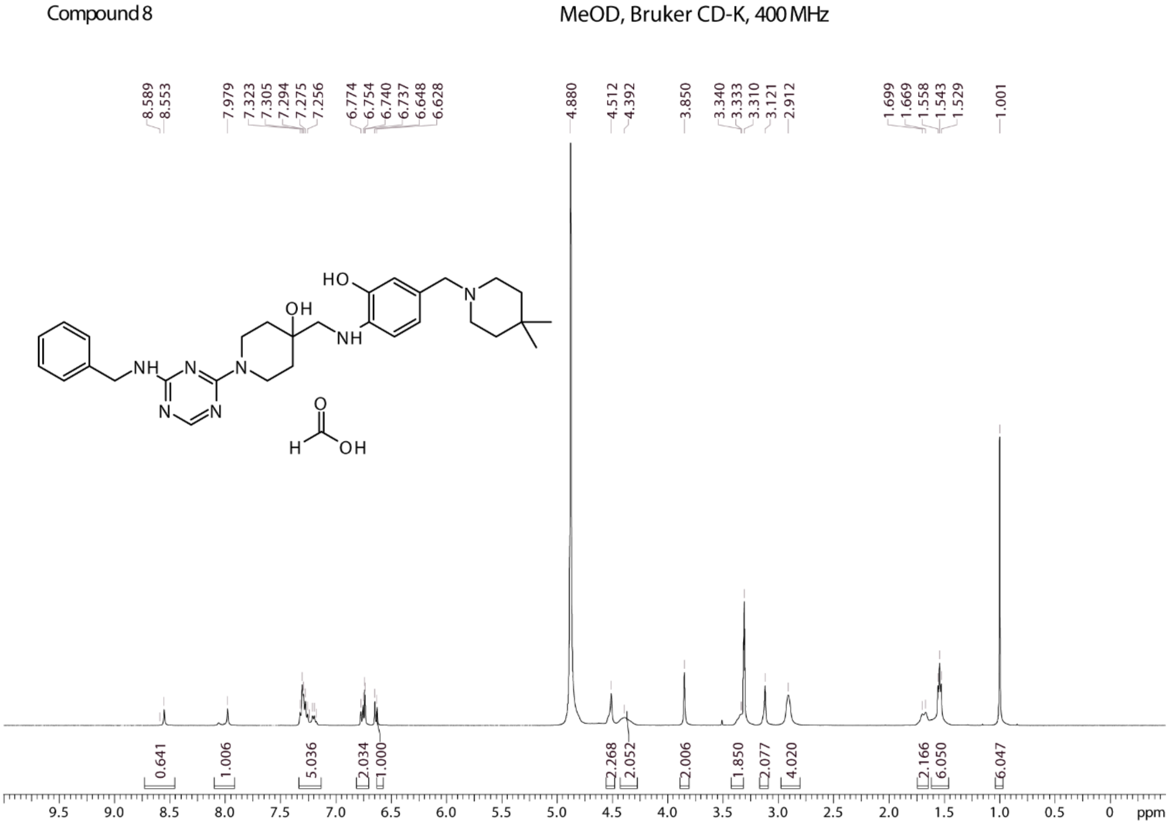

1H NMR (400 MHz, METHANOL-d4) δ = 8.55 (s, 1H), 7.98 (s, 1H), 7.33 - 7.14 (m, 5H), 6.81 - 6.71 (m, 2H), 6.64 (d, *J* = 8.2 Hz, 1H), 4.51 (br s, 2H), 4.39 (br s, 2H), 3.85 (s, 2H), 3.34 (br d, *J* = 2.8 Hz, 2H), 3.12 (s, 2H), 2.91 (br s, 4H), 1.68 (br d, *J* = 11.8 Hz, 2H), 1.54 (br t, *J* = 5.6 Hz, 6H), 1.00 (s, 6H).

Compound **8**, LC-MS analysis:

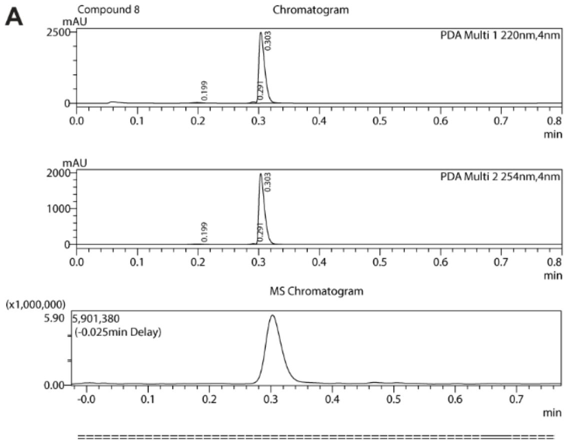

Integration Result

PDA Ch1 220nm

Peak#	Ret. Time	Height	Height%	USP Width	Area	Area%
1	0.199	18664	0.731	0.028	19687	1.164
2	0.291	45368	1.776	0.017	21929	1.296
3	0.303	2490073	97.493	0.018	1649957	97.540

PDA Ch2 254nm

Peak#	Ret. Time	Height	Height%	USP Width	Area	Area%
1	0.199	11334	0.565	0.027	11689	0.898
2	0.291	22083	1.101	0.017	10418	0.800
3	0.303	1972555	98.334	0.017	1279474	98.302

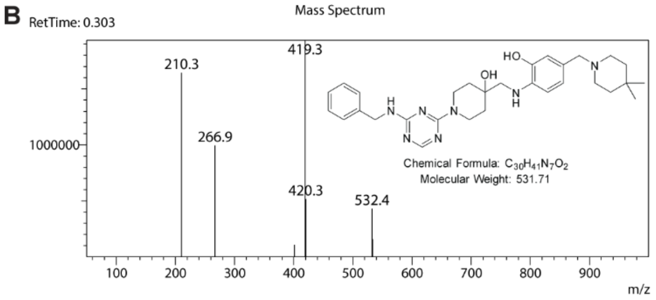

Supplement: Supplementary file 1 [file bg5c00184_si_001.pdf]
